# Interleukin-1 Receptor Modulation Using β-Substituted α-Amino-γ-Lactam Peptides From Solid-Phase Synthesis and Diversification

**DOI:** 10.3389/fchem.2020.610431

**Published:** 2020-12-21

**Authors:** Azade Geranurimi, Colin W. H. Cheng, Christiane Quiniou, France Côté, Xin Hou, Isabelle Lahaie, Amarilys Boudreault, Sylvain Chemtob, William D. Lubell

**Affiliations:** ^1^Département de Chimie, Université de Montréal, Montréal, QC, Canada; ^2^Department of Pharmacology & Therapeutics, McGill University, Montréal, QC, Canada; ^3^Hôpital Sainte-Justine Research Centre, Montréal, QC, Canada; ^4^Hôpital Maisonneuve-Rosemont Research Centre, Montréal, QC, Canada; ^5^Departments of Pediatrics, Pharmacology and Physiology, and Ophthalmology, Université de Montréal, Montréal, QC, Canada

**Keywords:** interleukin-1, solid-phase peptide synthesis, biased signaling, allosteric modulation, preterm (birth), retinopathy, β-turn, lactam

## Abstract

As a key cytokine mediator of inflammation, interleukin-1β (IL-1β) binds to the IL-1 receptor (IL-1R) and activates various downstream signaling mediators, including NF-κB, which is required for immune vigilance and cellular protection. Toward the development of IL-1-targeting therapeutics which exhibit functional selectivity, the all-D-amino acid peptide **1** (101.10, H-D-Arg-D-Tyr-D-Thr-D-Val-D-Glu-D-Leu-D-Ala-NH_2_) was conceived as an allosteric IL-1R modulator that conserves NF-κB signaling while inhibiting other IL-1-activated pathways. Employing β-hydroxy-α-amino-γ-lactam (Hgl) stereoisomers to study the conformation about the Thr^3^ residue in **1**, [(3*R*,4*S*)-Hgl^3^]-**1** (**2b**), among all possible diastereomers, was found to exhibit identical *in vitro* and *in vivo* activity as the parent peptide and superior activity to the α-amino-γ-lactam (Agl) counterpart. Noting the relevance of the β-hydroxyl substituent and configuration for the activity of (3*R*,4*S*)-**2b**, fifteen different β-substituted-Agl^3^ analogs of **1** (e.g., **2c-q**) have now been synthesized by a combination of solution- and solid-phase methods employing *N*-Fmoc-β-substituted-Agl^3^-Val-OH dipeptide building blocks. Introduction of a β-azido-Agl^3^ residue into the resin bound peptide and subsequent reduction and CuAAC chemistry gave access to a series of amine and triazole derivatives (e.g., **2h-q**). β-Substituted-[Agl^3^]-**1** analogs **2c-q** exhibited generally similar circular dichroism (CD) spectra as that of Hgl analog **2b** in water, presenting curve shapes indicative of β-turn structures. The relevance of the β-substituent was indicated in rodent models of preterm labor and retinopathy of prematurity (ROP), in which certain analogs inhibited preterm birth and vaso-obliteration, respectively, with activity similar to **1** and **2b**. The β-substituted-[Agl^3^]-**1** analogs exhibited functional selectivity on IL-1-induced signaling pathways. The described solid-phase method has provided discerning probes for exploring peptide structure-activity relationships and valuable leads for developing prototypes to treat inflammatory events leading to prematurity and retinopathy of prematurity, which are leading causes of infant morbidity and blindness respectively.

## Introduction

Inflammatory factor expression is induced primarily through signaling pathways triggered by interleukin-1β (IL-1β) (Gabay et al., 2010). This major pro-inflammatory cytokine stimulates various physiological effects leading ultimately to hyperthermia, hypotension, tissue destruction, and inflammation (Dinarello, 1997). The activity of IL-1β is critical for inflammatory responses to treat damaged tissue and to ward off invading pathogens. Uncontrolled IL-1β activity is, however, a pathogenic characteristic of many chronic conditions.

In reproductive tissue during pregnancy, toll-like receptors (TLRs) can recognize and discriminate bacterial pathogen-associated molecular patterns (PAMPs) (Elovitz et al., 2003; Ilievski et al., 2007). The recognition of PAMPs during intra-amniotic infection (e.g., chorioamnionitis) leads to up-regulation of TLRs (Kim et al., 2004) and release of pro-inflammatory IL-1β from immune cells. The latter plays a key role in the induction of both term and preterm labor (Romero et al., 1989). Engagement of TLRs also leads to the activation of nuclear factor kappa-light-chain-enhancer of activated B cells (NF-κB), a transcription factor that is involved in the expression of cytokines, such as IL-1β, chemokines, and antimicrobial defensin peptides (Choi et al., 2004). Activation of NF-κB is essential for maintaining immune vigilance against invading pathogens.

Regulation of TLR expression plays a key role in ischemic diseases of the retina, such as retinopathy of prematurity (ROP) (Xu and Wang, 2016). Multiple cell types in the retina express TLRs, including glia, retinal pigment epithelium (RPE), photoreceptor, and endothelial cells. In the pathogenesis of retinal ischemic diseases, activation of TLRs initiates signal transduction, leading to production of pro-inflammatory cytokines, such as IL-1β (Rivera et al., 2010, 2017; Xu and Wang, 2016; Beaudry-Richard et al., 2018). In response to hypoxia, retinal microglia cells produce IL-1β and trigger an inflammatory cascade involving IL-6 and IL-8 (Tosato and Jones, 1990). Endothelial cytotoxicity results from IL-1β-dependent retinal and sub-retinal injury in models of oxygen-induced retinopathy (OIR) (Tremblay et al., 2013; Zhou et al., 2016). Moreover, IL-1β has been linked to oxidative stress and is associated with retinal microvascular degeneration mediated by the pro-apoptotic guidance cues of semaphorin 3A (Sema3A) (Rivera et al., 2013).

The IL-1 receptor (IL-1R) complex is composed of the IL-1 receptor I (IL-1RI) and accessory protein (IL-1RAcP, occasionally referred to as IL-1R3) subunits, which activate multiple signaling pathways upon binding to IL-1β (Krumm et al., 2014). For example, the NF-κB protein complex is typically activated by IL-1β signaling which prompts cellular responses responsible for immune vigilance to counter bacterial and viral invasion. Other IL-1β-triggered signaling pathways involve kinases, such as c-Jun *N*-terminal kinases (JNK) (Roy et al., 2008) and Rho-associated kinase-2 (ROCK2), (Amano et al., 2010) which regulate the inflammation cascade, including the synthesis of pro-inflammatory cytokines, and the maturation, migration, and activity of T cells.

Two natural inhibitors of IL-1β signaling are the intrinsic IL-1 receptor antagonist (IL-1Ra) and the decoy receptor, IL-1 receptor type II (IL-1RII) (Krumm et al., 2014). IL-1Ra competes with IL-1 for the IL-1R binding site and prevents recruitment of IL-1RAcP. IL-1RII sequesters IL-1 but cannot form a signaling complex (Gabay et al., 2010). Current therapeutic strategies to counter pathological IL-1 signaling have been based on the mechanisms of these natural proteins. The three FDA-approved peptide-based therapies comprise: (1) the recombinant IL-1 receptor antagonist, Kineret, (2) the IL-1 Trap composed of a dimeric fusion of the ligand-binding domains of the extracellular portions of IL-1R1 and IL-1RAcP proteins, Rilonacept, and (3), a human monoclonal antibody targeting IL-1β, Canakinumab (Kaneko et al., 2019). These relatively large proteins have presented undesirable secondary effects in clinical settings including immunosuppression, which increases the risk for opportunistic infections, and pain at the site of injection (Opal et al., 1997; Roerink et al., 2017). Their failures in clinical trials may be due in part to drawbacks related to acting directly on the native orthosteric ligand and indiscriminately interfering with all signals triggered by IL-1β (Opal et al., 1997; Roerink et al., 2017). Selective agents are desired to differentially target IL-1β signaling pathways leading to immune vigilance and inflammation. For example, allosteric ligands which bind remotely from the orthosteric ligand binding site of IL-1RI have been pursued to modulate IL-1β activity by inducing biased signaling. Such allosteric modulators may be smaller molecules exhibiting improved bioavailability, potential for oral administration, protease resistance, and lower risks of toxicity.

The all D-amino acid heptapeptide rytvela (also named 101.10, **1**, D-Arg-D-Tyr-D-Thr-D-Val-D-Glu-D-Leu-D-Ala-NH_2_) was identified from a library of peptide sequences derived from the IL-1RAcP loop and juxtamembranous regions (Quiniou et al., 2008). Peptide **1** has exhibited potent, selective, and reversible non-competitive inhibition of IL-1β activity. For example, peptide **1** blocked IL-1β-induced human thymocyte cell proliferation *in vitro* (Jamieson et al., 2009) and demonstrated robust *in vivo* effects in models of hyperthermia and inflammatory bowel disease (Quiniou et al., 2008). Efforts to understand the biologically active conformation of **1** through covalent constraint have employed α-amino-γ-lactam (Agl) residues, (Jamieson et al., 2009; Ronga et al., 2010) so-called Freidinger-Veber lactams, (Freidinger et al., 1980; Freidinger, 2003) as well as their β-hydroxyl-α-amino-γ-lactam (Hgl) counterparts as rigid threonine mimics (St-Cyr et al., 2010, 2017; Geranurimi and Lubell, 2018a,b). Among the restricted analogs, [(3*R*,4*S*)-Hgl^3^]-**1** exhibited identical signaling behavior as the parent peptide *in vitro* (Geranurimi et al., 2019). Like **1**, [(3*R*,4*S*)-Hgl^3^]-**1** did not inhibit NF-κB signaling but exhibited strong inhibitory potency on IL-1β-induced phosphorylation of kinases (e.g., JNK and ROCK2) and expression of cytokines (e.g., IL-1 and cyclooxygenase-2 [COX-2]) in mouse macrophage cell lines. Moreover, [(3*R*,4*S*)-Hgl^3^]-**1** behaved like **1** in *in vivo* rodent models of preterm birth (PTB) and retinopathy of prematurity (ROP), respectively, delaying IL-1β-induced labor and curbing hyperoxia-induced vaso-obliteration.

In contrast to **1**, which exhibits a random coil circular dichroism (CD) spectrum in water, [(3*R*,4*S*)-Hgl^3^]-**1** displayed a CD curve shape indicative of a β-turn geometry. The corresponding [(3*R*)-Agl^3^]-**1** (**2a**) exhibited a similar CD curve shape as [(3*R*,4*S*)-Hgl^3^]-**1** (**2b**), however, Agl analog **2a** failed to exhibit inhibitory potency on p38 mitogen-activated protein kinases (p38 MAPK) and ROCK2. Moreover, Agl analog **2a** was inactive in the ROP model indicating the importance of the β-hydroxyl group for blocking signaling and activity in the pathways leading to hyperoxia-induced vaso-obliteration.

**Scheme 1:**
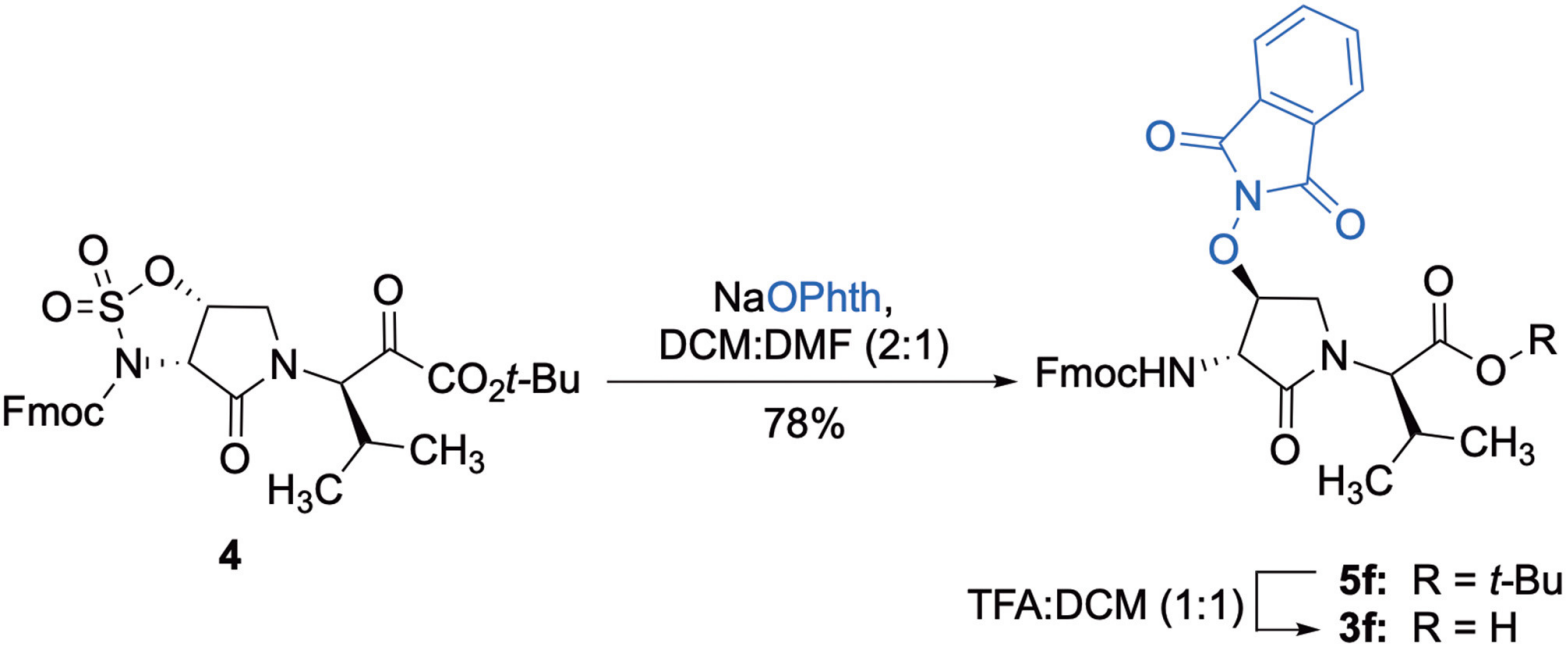
Synthesis of dipeptide **3f** by ring opening of cyclic sulfamidate **4** and ester cleavage.

The influence of the β-substituent of [(3*R*,4*S*)-Hgl^3^]-**1** (**2b**) on activity has now been studied further through the stereoselective synthesis of a series of β-substituted-Agl analogs. Routes have been developed to synthesize β-substituted-Agl dipeptides **3** by sequences featuring nucleophilic ring opening of cyclic sulfamidate **4**, which is derived from Fmoc-(3*R*,4*R*)-Hgl-Val-O*t*-Bu (Scheme 1). After ester removal, Fmoc-(3*R*,4*S*)-β-substituted-Agl-Val-OH analogs were obtained possessing β-azido **3c**, thiocyano **3d**, and methylthio **3e** substituents (Gulea et al., 2003; Geranurimi and Lubell, 2018a,b). Moreover, ring opening of sulfamidate **4** with hydroxyphthalimide gave access to protected aminoxy analog **3f**. Four novel lactam analogs of **1** (e.g., **2c**-**f**) were synthesized by employing β-substituted-Agl dipeptides **3c**-**f** as building blocks in standard Fmoc-based solid phase synthesis protocols (Lubell et al., 2005). Furthermore, modification of the azide and hydroxyphthalimide moieties of the corresponding protected peptides **8c** and **8f** on resin gave β-amino and β-aminoxy-Agl^3^ analogs **2h** and **2g**, accordingly. Modification of β-amino-Agl^3^ resin **8h** by acylation, carbamylation and guanidinylation yielded lactam analogs **2i-k**. Finally, copper-catalyzed azide alkyne cycloaddition (CuAAC) on β-azido-Agl^3^ resin **8c** furnished 1,2,3-triazoles which upon resin cleavage and removal of protection gave lactams **2l-q**.

Fifteen new (3*R*,4*S*)-β-substituted-Agl^3^ analogs of **1** (e.g., **2c**-**q**) were synthesized using the combination of the solution- and solid-phase synthesis protocols. The hydroxyl group of **2b** has been replaced by a variety of functional groups with potential to contribute to hydrogen bonds as donors and acceptors, to salt bridges as protonated ammonium ions, and to π-cation interactions as aromatic donors. Furthermore, the novel β-substituents exhibited limited effect on conformation in comparative analyses of the curve shapes of (3*R*,4*S*)-Hgl analog **2b** with those of the other (3*R*,4*S*)-β-substituted-Agl^3^ analogs (**2c**-**q**) using circular dichroism spectroscopy.

The β-substituent on lactams **2c**-**q** exhibited notable effects on inflammatory gene transcription (IL-1β and COX-2) and kinase phosphorylation *in vitro* in RAW 264.7 macrophages. In a reporter gene assay, the β-substituted-Agl analogs **2c**-**q** behaved like peptide **1** and Hgl analog **2b** and did not exhibit effects on IL-1β-induced NF-κB signaling. Based on preliminary *in vitro* analyses, certain analogs were selected for examination in *in vivo* rodent models of preterm labor (PTL) and oxygen-induced retinopathy (OIR). These investigations have identified a novel set of (3*R*,4*S*)-β-substituted-Agl^3^ analogs that exhibited *in vivo* effects comparable to the (3*R*,4*S*)-Hgl counterpart **2b**. In summary, this study provides valuable methods for studying structure-activity relationships of biologically active peptides and a deeper understanding of the relevance of lactam β-substituents on IL-1R modulator activity.

## Materials and Methods

### General Chemistry Methods

Unless otherwise specified, all non-aqueous reactions were performed under an inert argon atmosphere. All glassware was dried with a flame and a flushing stream of argon gas or stored in the oven and let cool under an inert atmosphere prior to use. Anhydrous solvents (THF, DCM, MeCN, MeOH, and DMF) were obtained by passage through solvent filtration systems (Glass Contour, Irvine, CA). Anhydrous solvents were transferred by syringe. Reaction mixture solutions (after aqueous workup) were dried over anhydrous MgSO_4_ or Na_2_SO_4_, filtered, and rotary-evaporated under reduced pressure. Column chromatography was performed on 230–400 mesh silica gel, and thin-layer chromatography was performed on alumina plates coated with silica gel (Merck 60 F_254_ plates). Visualization of the developed chromatogram was performed by UV absorbance or staining with iodine or potassium permanganate solutions. Specific rotations, [α]^D^ values, were calculated from optical rotations measured at 25°C in CHCl_3_ at the specified concentrations (*c* in g/100 mL) in a 0.5 dm cell length (l) on a Anton Paar Polarimeter, MCP 200 at 589 nm, using the general formula: ${\left[\alpha \right]}_{25}^{\text{D}}$ = (100 × α)/(l × *c*). Nuclear magnetic resonance spectra (^1^H NMR, ^13^C NMR) were recorded on a Bruker 300 MHz spectrometer. ^1^H NMR and decoupled ^13^C[^1^H] NMR spectra were measured in and referenced to CDCl_3_ (7.26 ppm, 77.16 ppm) as specified below. Coupling constant *J* values were measured in Hertz (Hz) and chemical shift values in parts per million (ppm). High resolution mass spectrometry (HRMS) data were obtained in electrospray ionization (ESI-TOF) mode by the Centre Régional de Spectrométrie de Masse de l'Université de Montréal. Protonated molecular ions [M + H]^+^, and sodium adducts [M + Na]^+^ were used for empirical formula confirmation. Analytical LCMS and HPLC analyses were performed on either a CSH-C18, 4.6 X100 mm, 5 μm column with a flow rate of 0.8 mL/min or CE-C18 3 X 50 mm, 2.7 μm column with a flow rate of 0.4 mL/min using appropriate gradients from X-Y% of MeOH [0.1% formic acid (FA)] or MeCN (0.1% FA) in H_2_O (0.1% FA) over 10 min: a) 10–90%, b) 50-90%, c) 30–60%, d) 5–60%, e) 30–90%, f) 20–40%.

All final peptides were purified using the respective conditions below on a Waters™ preparative HPLC instrument with UV detection at 214, 254, and 280 nm and one of the following systems: a reverse-phase Gemini™ C18 column (21.2 × 250 mm, 5 μm) using a flow rate of 10 mL/min over 40 min; a C18 Atlantis column (19 × 100 mm, 5 um) using a flow rate of 24 mL/min over 15 min; a RP-Polar column (19 × 100 mm, 4 μm) using a flow rate of 24 mL/min over 15 min. The appropriate gradients from X-Y% of MeOH (or MeCN) containing 0.1% FA in H_2_O (0.1% FA) over time were used on the following columns: A) 10–90%/30 min MeOH (0.1% FA) in H_2_O (0.1% FA), Gemini™ C18 column; B) 10–90%/10.0 min MeOH (0.1% FA) in H_2_O (0.1% FA), Atlantis C18 column; C) 30–90%/10 min MeOH (0.1% FA) in H_2_O (0.1% FA), Atlantis C18 column; D) 40-90%/10 min MeCN (0.1% FA) in H_2_O (0.1% FA), RP-Polar column; E) 30–90%/10 min MeCN (0.1% FA) in H_2_O (0.1% FA), C18 Atlantis column; F) 0–50%/ 9 min MeOH (0.1% FA) in H_2_O (0.1% FA), C18 Atlantis column.

### Chemical Reagents

Unless specified otherwise, commercially available reagents were purchased from Aldrich, A & C American Chemicals Ltd., Fluka and Advanced Chemtech™ and used without further purification: copper(I)iodide, phenylacetylene, 4-ethynyltoluene, 3-ethynylaniline, 1,1-dimethylpropargylamine (90% remainder H_2_O), propargylamine, propargylalcohol, acetic acid, tris(2-carboxyethyl)phosphine hydrochloride, acetic anhydride, potassium cyanate, 1,3-bis(*tert*-butoxycarbonyl)-2-methyl-2-thiopseudourea, triethylamine, mercuric chloride, piperidine, DIEA, TFA, TES, TEA, HBTU, polystyrene Rink amide resin (75–100 mesh, 1%, DVB with a 0.5 mmol/g loading). All commercially available amino acids [e.g., Fmoc-D-Ala-OH, Fmoc-D-Leu-OH, Fmoc-D-Glu(*t*-Bu)-OH, Fmoc-D-Tyr(*t*-Bu)-OH, Boc-D-Arg(Pmc)-OH] were purchased from GL Biochem™ and used as received. Solvents were obtained from VWR international. Human rIL-1β (200-01B) was from PeproTech, lipopolysaccharide (LPS) *Escherichia coli* endotoxin (L2630) from Sigma-Aldrich, H-rytvela-NH_2_ (peptide **1**) from Elim Biopharmaceuticals, Hayward, CA, and Kineret (Anakinra) from Sobi, Biovitrum Stockholm, Sweden.

#### [(3*R*, 4*S*)-4-(N_3_)Agl^3^]-1 (2c)


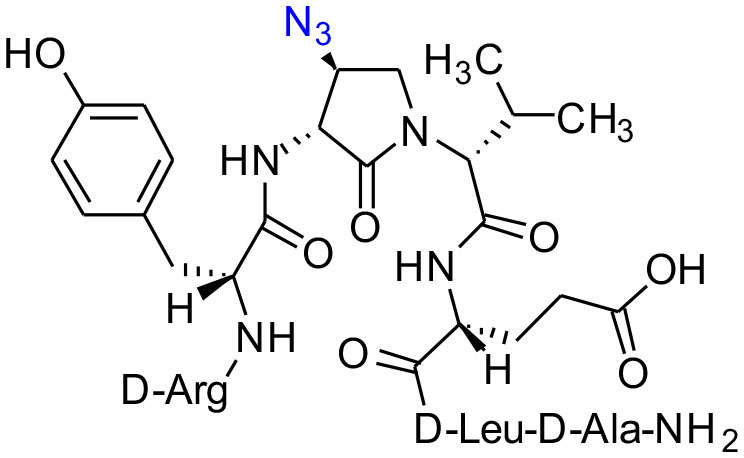
 A 10-mL plastic filtration tube equipped with a polyethylene filter and stopcock was charged with polystyrene Rink amide resin (75–100 mesh, 1%, DVB with a 0.5 mmol/g loading, 100 mg, 50.0 μmol), followed by DCM (7 mL). The tube was sealed, shaken for 30 min to induce swelling and the liquid phase was removed by filtration. The Fmoc group was cleaved from the resin by treatment with a freshly prepared 20% piperidine in DMF solution (5 mL), shaking for 30 min, and removal of the liquid phase by filtration. The resin was washed repeatedly (3 × per solvent) with DMF and DCM (10 mL per wash for 6 min), and the liquid phase was removed by filtration. The presence of the free amine resin was confirmed by a positive Kaiser test. Peptide elongation was conducted by treating the DMF-swollen free amine resin with a freshly prepared acylation solution composed of Fmoc-D-Ala-OH (3 eq), HBTU (3 eq), and DIEA (6 eq) in DMF (4–7 mL). After agitating for 3–5 h, at rt, the resin was filtered, the Fmoc group was cleaved as described above and peptide coupling was performed using the following sequence of acids: Fmoc-D-Leu-OH, Fmoc-D-Glu(*t*-Bu)-OH, *N*-Fmoc-(3*R*,4*S*)-4-(N_3_)Agl-*R*-Val-OH (**3c**), Fmoc-D-Tyr(*t*-Bu)-OH, and Boc-D-Arg(Pmc)-OH. For the coupling of *N*-Fmoc-(3*R*,4*S*)-β-N_3_-Agl-*R*-Val-OH (**3c**), only a stoichiometric quantity of dipeptide acid was used; for Fmoc-D-Tyr(*t*-Bu)-OH, coupling was repeated twice at higher reaction concentration. Synthetic progress was monitored using a combination of the Kaiser test and LC-MS analyses on TFA-cleaved resin aliquots, which were concentrated and dissolved in mixtures of water and MeCN. The completed peptide sequence **8c** with 80% crude purity was cleaved from the resin by treatment with TFA/H_2_O/TES (3 mL, 95/2.5/2.5, v/v/v) with shaking for 3 h. The liquid phase was removed by filtration and collected. The resin was washed twice with TFA and the combined liquid phases were concentrated in vacuo. The residue was dissolved in a minimal volume of acetonitrile, precipitated with ice-cold diethyl ether, and centrifuged at 7,000 rpm. The supernatant was removed by decantation and the precipitate was collected. The precipitation and collection processes were repeated on the supernatant. The combined white solid precipitate was dissolved in water (5 mL), freeze-dried to give a white powder (80% crude purity), and purified using method A with UV detection at 214 nm. Fractions containing pure peptide were combined and lyophilized to afford peptide [(3*R*,4*S*)-β-N_3_-Agl^3^]-**1** (**2c**, 9 mg, 22% yield of >95% purity): LCMS, 10–90%/10 min MeOH (0.1% FA) in water (0.1% FA), RT 8.3 min, and 10–90%/10 min MeCN (0.1% FA) in water (0.1% FA), RT 5.8 min; HRMS (ESI^+^) calcd m/z for C_38_H_60_N_14_O_10_ [M+H]^+^, 873.4690 found 873.4680.

#### [(3*S*, 4*S*)-4-(NCS)Agl^3^]-1 (2d)


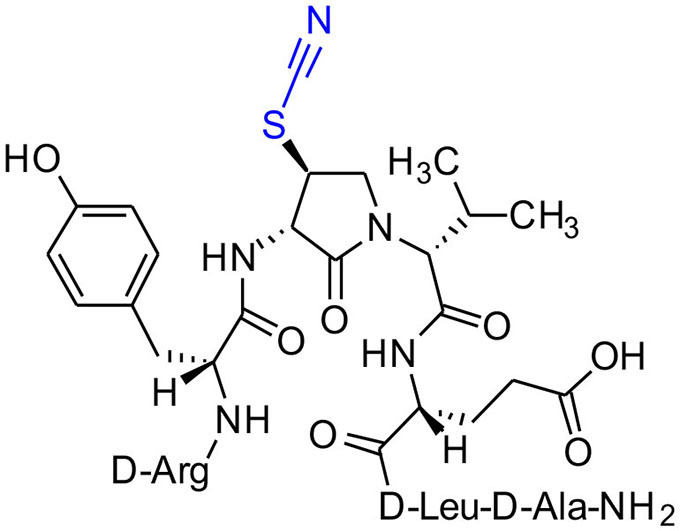
 Employing *N*-Fmoc-(3*R*,4*S*)-4-(NCS)Agl-*R*-Val-OH (**3d**) in the representative procedure described for peptide **2c**, [(3*S*,4*S*)-4-(NCS)Agl^3^]-**1** (**2d**) was synthesized and purified using method A with UV detection at 214 nm (3 mg, 3% yield of >95% purity); LCMS, 10–90%/10 min MeOH (0.1% FA) in water (0.1% FA), RT 8.3 min, and 10–90%/10 min MeCN (0.1% FA) in water (0.1% FA), RT 5.8 min; HRMS (ESI^+^) calcd m/z for C_39_H_60_N_12_O_10_S [M+H]^+^, 889.4349 found 889.4342.

#### [(3*S*, 4*S*)-4-(MeS)Agl^3^]-1 (2e)


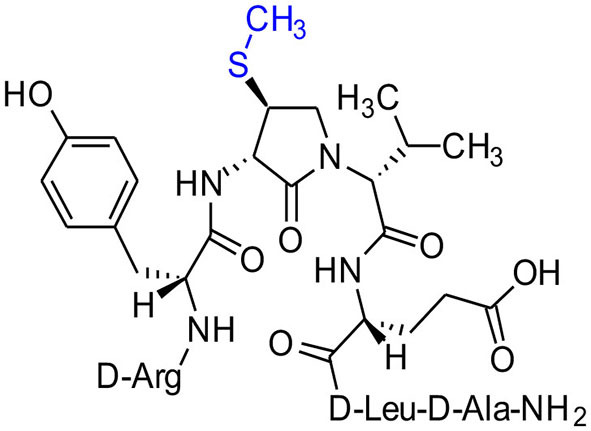
 Employing *N*-Fmoc-(3*S*,4*S*)-4-(MeS)Agl-*R*-Val-OH (**3e**) in the representative procedure described for peptide **2c**, [(3*S*,4*S*)-4-(MeS)Agl^3^]-**1** (**2e**) was synthesized and purified using method A with UV detection at 214 nm (4.5 mg, 10% yield of >95% purity); LCMS 10–90%/10 min MeOH (0.1% FA) in water (0.1% FA), RT 8.7 min, and 10–90%/10 min MeCN (0.1% FA) in water (0.1% FA), RT 5.9 min; HRMS (ESI^+^) calcd m/z for C_39_H_63_N_11_O_10_S [M+H]^+^, 878.4553 found 878.4559.

#### [(3*R*,4*S*)-4-(PhthO)Agl^3^]-1 (2f)


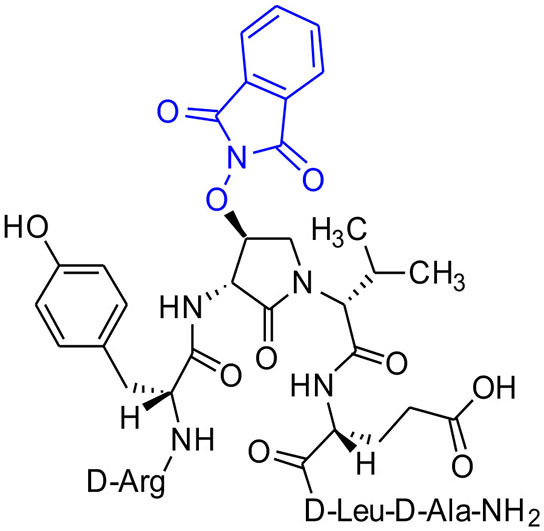
 Employing *N*-Fmoc-(3*R*,4*S*)-4-(PhthO)Agl-*R*-Val-OH (**3f**) in the representative procedure described for peptide **2c**, [(3*R*,4*S*)-4-(PhthO)Agl^3^]-**1** (**2f**) was synthesized and purified using method A with UV detection at 214 nm (5.5 mg, 11% yield of >95% purity); LCMS 30–60%/10 min MeOH (0.1% FA) in water (0.1% FA), RT 8.8 min, and 5–60%/10 min MeCN (0.1% FA) in water (0.1% FA), RT 7.3 min; HRMS (ESI^+^) calcd m/z for C_46_H_64_N_12_O_13_ [M+H]^+^, 993.4789 found 993.4786.

#### [(3*R*,4*S*)-4-(H_2_NO)Agl^3^]-1 (2g)


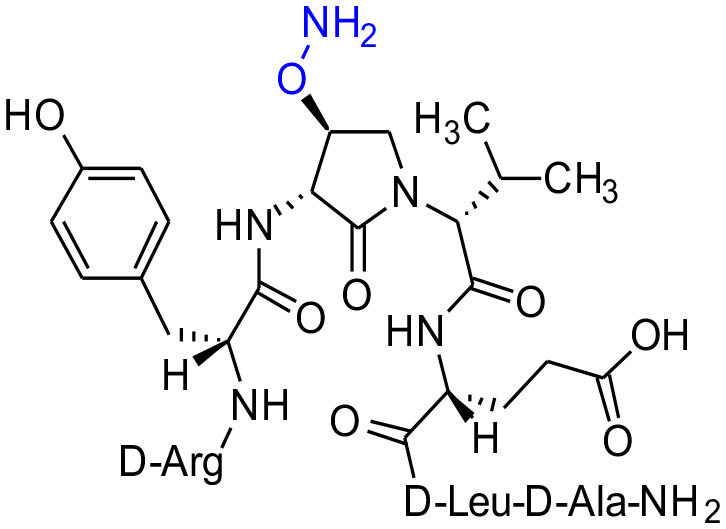
 In a 10-mL plastic filtration tube equipped with a polyethylene filter, Boc-D-Arg(Pmc)-D-Tyr(*t*-Bu)-(3*R*,4*S*)-4-(PhthO)Agl-D-Val-D-Glu(*t*-Bu)-D-Leu-D-Ala-Rink amide resin **8f** (100 mg, 50.0 μmol) was swollen in MeOH/DCM (1/1, v/v, 4 mL), treated with hydrazine monohydrate (73 μL, 1.50 μmol), and agitated for 5 h at rt using an automated shaker (Villadsen et al., 2017). The resin was filtered, washed with DMF (3 × 10 mL) and DCM (3 × 10 mL), dried under vacuum, and stored in the fridge. Resin **8g** was cleaved and the crude peptide was recovered as described for peptide **2c**; LCMS analysis indicated 38% crude purity. Purification using method A with UV detection at 280 nm and collection of the pure fractions afforded [(3*R*,4*S*)-4-(H_2_NO)Agl^3^]-**1** (**2g**, 2.2 mg, 5% yield of >95% purity); LCMS 10–90%/10 min MeOH (0.1% FA) in water (0.1% FA), RT 6.6 min, and 10–90%/10 min MeCN (0.1% FA) in water (0.1% FA), RT 5.2 min; HRMS (ESI^+^) calcd m/z for C_38_H_62_N_12_O_11_ [M+H]^+^, 863.4661 found 863.4687.

#### [(3*R*,4*S*)-4-(H_2_N)Agl^3^]-1 (2h)


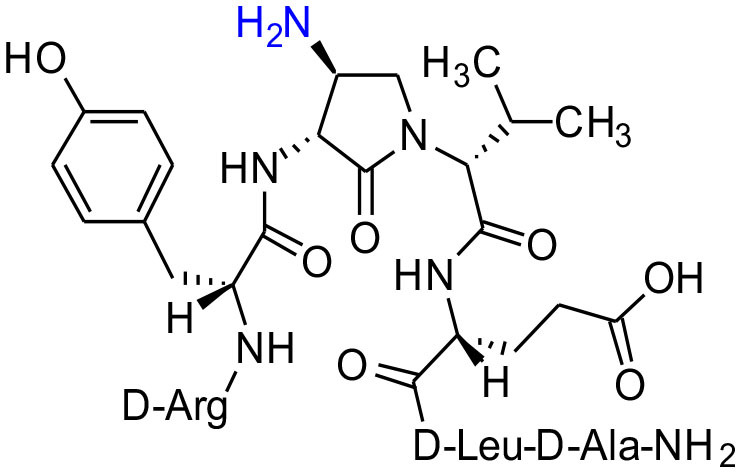
 In a 10-mL plastic filtration tube equipped with a polyethylene filter, Boc-D-Arg(Pmc)-D-Tyr(*t*-Bu)-(3*R*,4*S*)-4-(N_3_)Agl-D-Val-D-Glu(*t*-Bu)-D-Leu-D-Ala-Rink amide resin **8c** (100 mg, 50.0 μmol) was swollen in THF/H_2_O (9/1, v/v, 4 mL), treated with tris(2-carboxyethyl)phosphine hydrochloride (40 μL, 150 μmol), and agitated for 4 h at rt on an automated shaker (Pandey et al., 2013). The resin was filtered, washed with DMF (3 × 10 mL), MeOH (3 × 10 mL), THF (3 × 10 mL), and DCM (3 × 10 mL), dried under vacuum, and stored in the fridge. Resin **8h** was cleaved and the crude peptide was recovered as described for peptide **2c**; LCMS analysis indicated 70% crude purity. Purification using method A with UV detection at 280 nm, and collection and freeze-drying of the pure fractions afforded [(3*R*,4*S*)-4-(H_2_N)Agl^3^]-**1** (**2h**, 7.6 mg, 18% yield of >95% purity): LCMS 10–90%/10 min MeOH (0.1% FA) in water (0.1% FA), RT 5.8 min, and 50-90%/10 min MeCN (0.1% FA) in water (0.1% FA), RT 1.0 min; HRMS (ESI^+^) calcd m/z for C_38_H_62_N_12_O_10_ [M+H]^+^, 847.4785 found 847.4780.

#### [(3*R*,4*S*)-4-(AcHN)Agl^3^]-1 (2i)


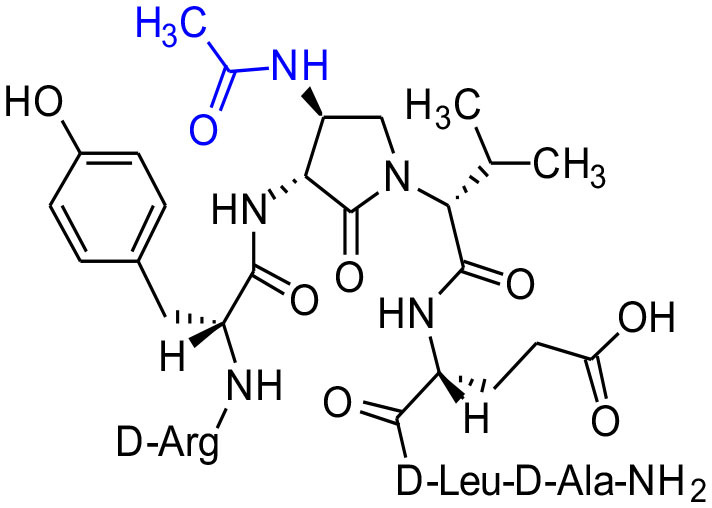
 A 10-mL plastic filtration tube equipped with a polyethylene filter was charged with Boc-D-Arg(Pmc)-D-Tyr(*t*-Bu)-(3*R*,4*S*)-4-(H_2_N)Agl-D-Val-D-Glu(*t*-Bu)-D-Leu-D-Ala-Rink amide resin **8h** (100 mg, 50.0 μmol), which was swollen in anhydrous DMF (2.00 mL) at rt, treated with acetic anhydride (14 μL, 150 μmol) followed by DIEA (52 μL, 300 μmol), and agitated at rt for 3 h. Water (0.5 mL) was added to the tube, which was agitated for 30 min. The resin was filtered, washed with DMF (3 × 10 mL) and DCM (3 × 10 mL), dried under vacuum, and stored in the fridge. Resin **9i** was cleaved and the crude peptide was recovered as described for peptide **2c**; LCMS analysis indicated 41% crude purity. Purification using method A with UV detection at 214 nm, and collection and freeze-drying of the pure fractions afforded [(3*R*,4*S*)-4-(AcHN)Agl^3^]-**1** (**2i**, 4 mg, 9% yield of >95% purity); LCMS 10–90%/10 min MeOH (0.1% FA) in water (0.1% FA), RT 7.5 min, and 10–90%/10 min MeCN (0.1% FA) in water (0.1% FA), RT 5.9 min; HRMS (ESI^+^) calcd m/z for C_40_H_64_N_12_O_11_ [M+H]^+^, 889.4890 found 889.4895.

#### [(3*R*,4*S*)-4-(H_2_N(C=O)HN)Agl^3^]-1 (2j)


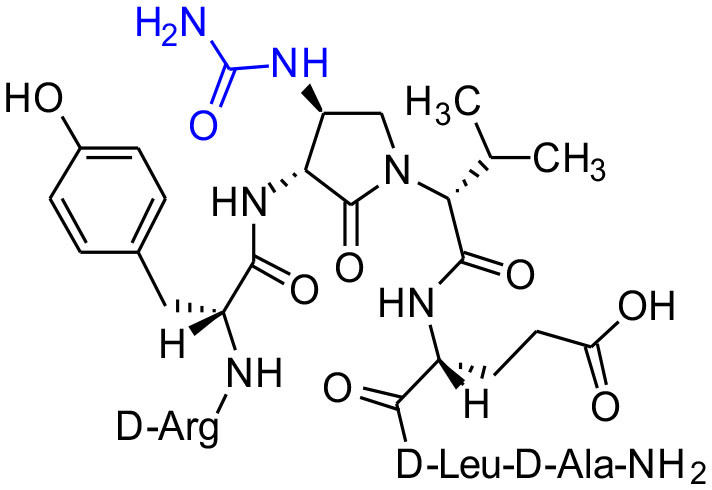
 A 10-mL plastic filtration tube equipped with a polyethylene filter was charged with Boc-D-Arg(Pmc)-D-Tyr(*t*-Bu)-(3*R*,4*S*)-4-(H_2_N)Agl-D-Val-D-Glu(*t*-Bu)-D-Leu-D-Ala-Rink amide resin **8h** (100 mg, 50.0 μmol), which was swollen in THF (2.00 mL) at rt, treated with potassium cyanate (10 μg, 150 μmol) followed by AcOH (8 μL,150 μmol) and H_2_O (0.1 mL), and agitated at rt for 4 h on an automated shaker. The resin was filtered, washed with DMF (3 × 10 mL) and DCM (3 × 10 mL), dried under vacuum, and stored in the fridge. Resin **9j** was cleaved and the crude peptide was recovered as described for peptide **2c**; LCMS analysis indicated 57% purity. Purification using method A with UV detection at 214 nm, and collection and freeze-drying of the pure fractions afforded [(3*R*,4*S*)-4-(H_2_N(C=O)HN)Agl^3^]-**1** (**2j**, 4 mg, 8% yield of >95% purity); LCMS 10-90%/10 min MeOH (0.1% FA) in water (0.1% FA), RT 7.3 min, and 10–90%/10 min MeCN (0.1% FA) in water (0.1% FA), RT 5.1 min; HRMS (ESI^+^) calcd m/z for C_39_H_63_N_13_O_11_ [M+H]^+^, 890.4843 found 890.4841.

#### [(3*R*,4*S*)-4-(H_2_N(C=NH)HN)Agl^3^]-1 (2k)


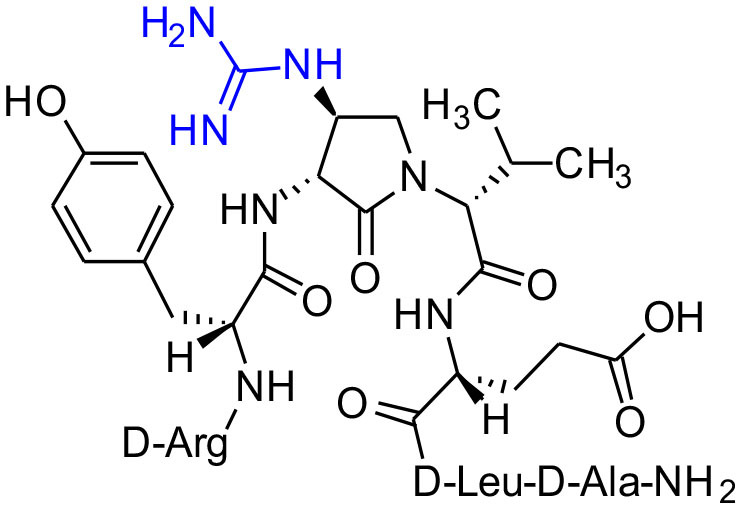
 A 10-mL plastic filtration tube equipped with a polyethylene filter was charged with Boc-D-Arg(Pmc)-D-Tyr(*t*-Bu)-(3*R*,4*S*)-4-(H_2_N)Agl-D-Val-D-Glu(*t*-Bu)-D-Leu-D-Ala-Rink amide resin **8h** (100 mg, 50.0 μmol), and washed with DMF (× 6), DCM (× 6), dry DCM (× 3) and dry DMF (× 6). The resin was swollen in dry DMF (4 mL), treated with 1,3-bis(*tert*-butoxycarbonyl)-2-methyl-2-thiopseudourea (22 μL, 75 μmol) and triethylamine (63 μL, 450 μmol), stirred for 5 min, treated with HgCl_2_ (40 μg, 150 μmol) in dry DMF (0.5 mL), and agitated for 2 h, at rt. The resin was washed with DMF (× 6), and DCM (× 6), dried under vacuum, and stored in the fridge. Resin **9k** was cleaved and the crude peptide was recovered as described for peptide **2c**; LCMS analysis indicated 62% purity. Purification using method A with UV detection at 280 nm, and collection and freeze-drying of the pure fractions afforded [(3*R*,4*S*)-4-(H_2_N(C=NH)HN)Agl^3^]-**1** (**2k**, 4 mg, 9% yield of >95% purity); LCMS 10–90%/10 min MeOH (0.1% FA) in water (0.1% FA), RT 5.9 min, and 5–60%/10 min MeCN (0.1% FA) in water (0.1% FA), RT 4.6 min; HRMS (ESI^+^) calcd m/z for C_39_H_64_N_14_O_10_ [M+H]^+^, 889.5003 found 889.5006.

#### [(3*R*,4*S*)-4-(4′-Phenyltriazolyl)Agl^3^]-1 (2l)


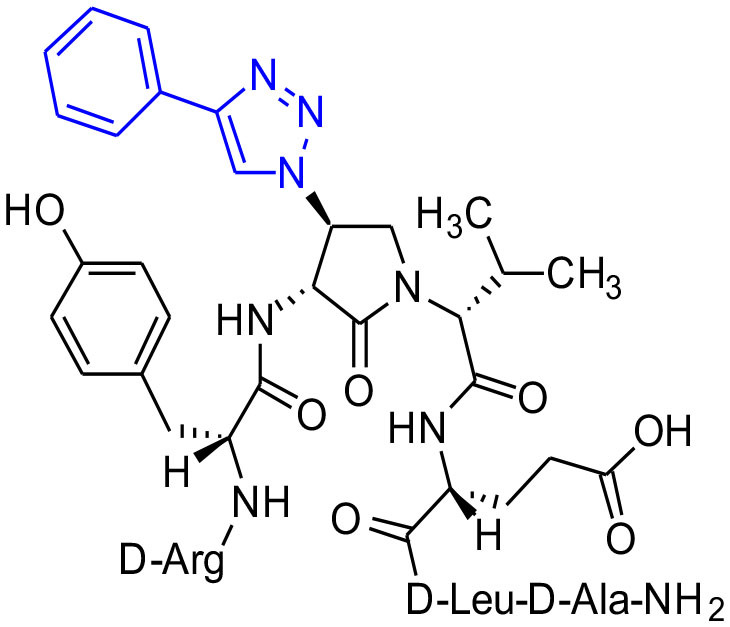
 In a 10-mL plastic filtration tube equipped with a polyethylene filter, Fmoc-D-Arg(Pmc)-D-Tyr(*t*-Bu)-(3*R*,4*S*)-4-(N_3_)Agl-D-Val-D-Glu(*t*-Bu)-D-Leu-D-Ala-Rink amide resin **8c** (100 mg, 50.0 μmol) was swollen in anhydrous DCM (2 mL), treated with copper(I)iodide (14 mg, 75.0 μmol) and DIEA (26 μL, 150 μmol), followed by phenylacetylene (20 μL, 180 μmol) and acetic acid (9 μL, 150 μmol), and shaken at rt for 18 h (Shao et al., 2011), filtered and washed with DMF (× 3) and DCM (× 3). Resin **10l** was cleaved and the crude peptide was recovered as described for peptide **2c**; LCMS analysis indicated 74% purity. Purification using method A with UV detection at 254 nm, and collection and freeze-drying of the pure fractions afforded [(3*R*,4*S*)-4-(4'-phenyltriazolyl)Agl^3^]-**1** (**2l**, 8 mg, 17% yield of >95% purity): LCMS 10–90%/10 min MeOH (0.1% FA) in water (0.1% FA), RT 9.6 min, and 10–90%/10 min MeCN (0.1% FA) water (0.1% FA), RT 6.5 min; HRMS (ESI^+^) calcd m/z for C_46_H_66_N_14_O_10_ [M+H]^+^, 975.5159 found 975.5147.

#### [(3*R*,4*S*)-4-(4′-p-Methylphenyltriazolyl)Agl^3^]-1 (2m)


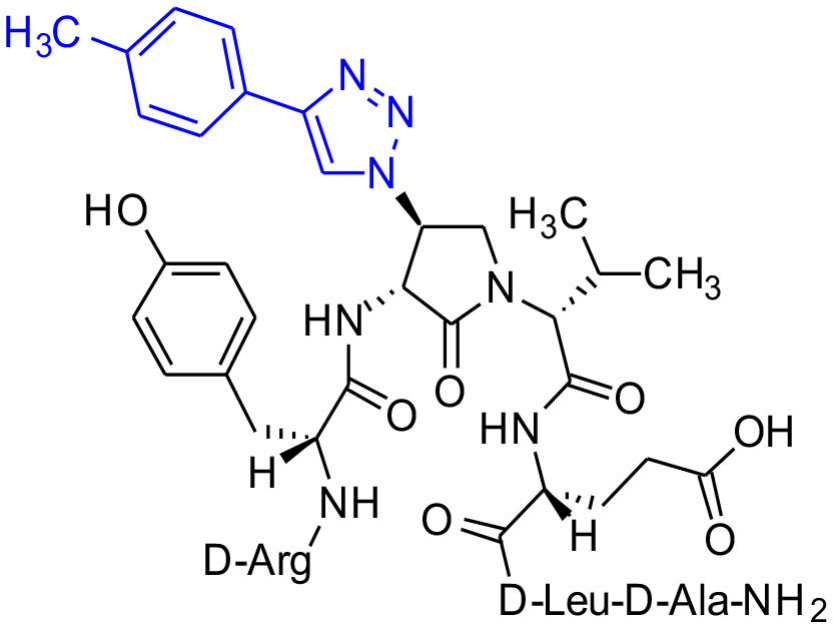
 Employing the representative procedures described for the synthesis of peptide **2l**, using resin **8c** (100 mg, 50.0 μmol) and 4-ethynyltoluene (22.8 μL, 180 μmol), peptide **2m**, was synthesized and indicated to be of 71% purity by LCMS analysis. Purification using method C with UV detection at 254 nm, and collection and freeze-drying of the pure fractions afforded [(3*R*,4*S*)-4-(4'-*p*-methylphenyltriazolyl)Agl^3^]-**1** (**2m**, 7 mg, 16% yield of >95% purity); LCMS 30-90%/10 min MeOH (0.1% FA) in water (0.1% FA), RT 9.5 min, and 20–40%/10 min MeCN (0.1% FA) in water (0.1% FA), RT 6.5 min; HRMS (ESI^+^) calcd m/z for C_47_H_68_N_14_O_10_ [M+H]^+^, 989.5316 found 989.5307.

#### [(3*R*,4*S*)-4-(4′-m-Aminophenyltriazolyl)Agl^3^]-1 (2n)


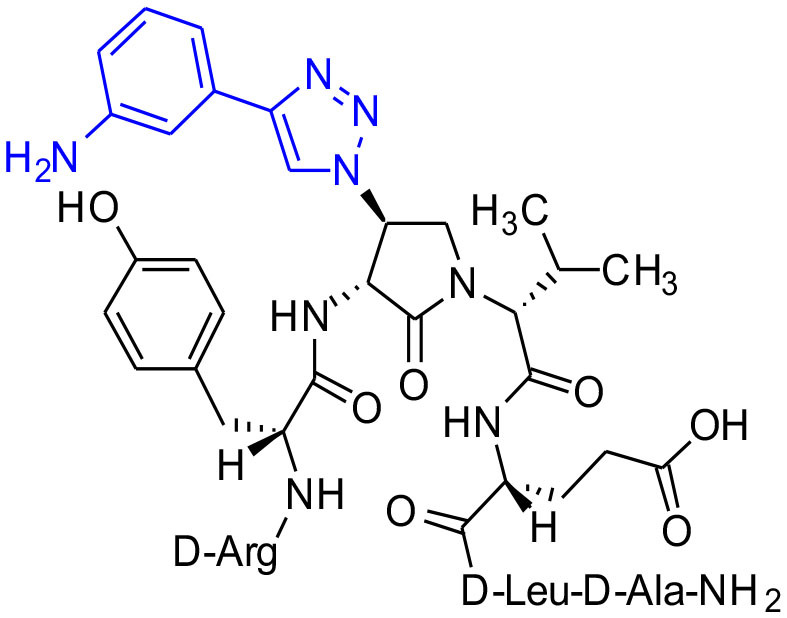
 Employing the representative procedures described for the synthesis of peptide **2l**, using resin **8c** (100 mg, 50.0 μmol) and 3-ethynylaniline (20.3 μL, 180 μmol), peptide **2n**, was synthesized and indicated to be of 69% purity by LCMS analysis. Purification using method D with UV detection at 254 nm, and collection and freeze-drying of the pure fractions afforded [(3*R*,4*S*)-4-(4'-*m*-aminophenyltriazolyl)Agl^3^]-**1** (**2n**, 6 mg, 12% yield of >95% purity); LCMS 5–60%/10 min MeOH (0.1% FA) in water (0.1% FA), RT 7.9 min, and 5–60%/10 min MeCN (0.1% FA) in water (0.1% FA), RT 6.1 min; HRMS (ESI^+^) calcd m/z for C_46_H_67_N_15_O_10_ [M+H]^+^, 990.5268 found 990.5259.

#### [(3*R*,4*S*)-4-(4′-(1,1-Dimethyl)aminomethyltriazolyl)Agl^3^]-1 (2o)


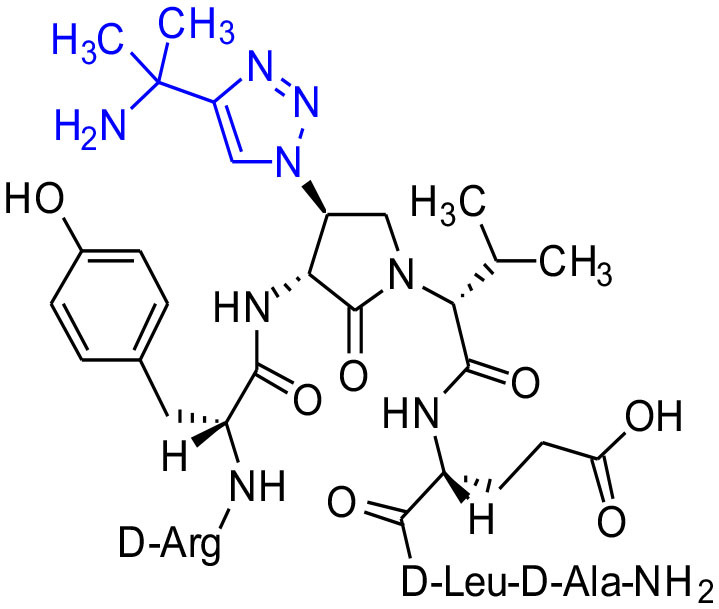
 Employing the representative procedures described for the synthesis of peptide **2l**, using resin **8c** (100 mg, 50.0 μmol) and 1,1-dimethylpropargylamine (90% remainder H_2_O, 15.0 μL, 180 μmol), peptide **2o** was synthesized and indicated to be of 73% purity by LCMS analysis. Purification using method B with UV detection at 214 nm, and collection and freeze-drying of the pure fractions afforded [(3*R*,4*S*)-4-(4'-(1,1-dimethyl)amino-methyltriazolyl)Agl^3^]-**1** (**2o**, 6 mg, 13% yield of >95% purity); LCMS 10–90%/10 min MeOH (0.1% FA) in water (0.1% FA), RT 6.3 min, and 5-60%/10 min MeCN (0.1% FA) in water (0.1% FA), RT 4.9 min; HRMS (ESI^+^) calcd m/z for C_43_H_69_N_15_O_10_ [M+H]^+^, 956.5425 found 956.5408.

#### [(3*R*,4*S*)-4-(4′-Aminomethyltriazolyl)Agl^3^]-1 (2p)


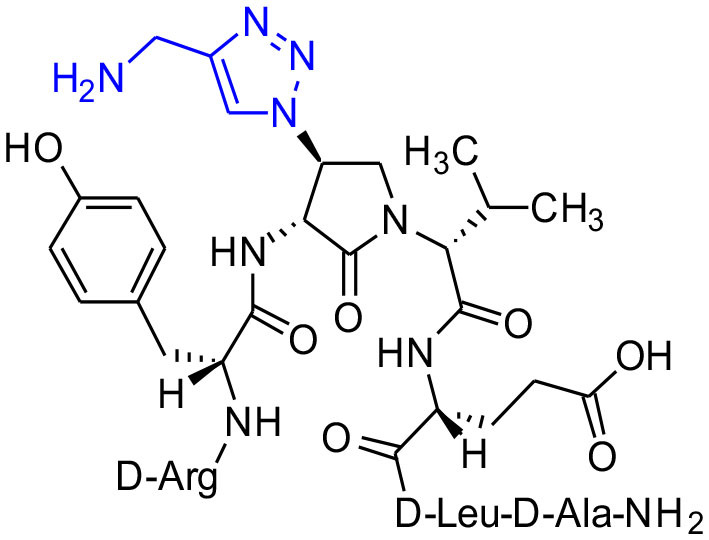
 Employing the representative procedures described for the synthesis of peptide **2l**, using resin **8c** (100 mg, 50.0 μmol) and propargylamine (12 μL, 180 μmol), peptide **2p** was synthesized and indicated to be of 53% purity by LCMS analysis. Purification using method E with UV detection at 254 nm, and collection and freeze-drying of the pure fractions afforded [(3*R*,4*S*)-4-(4'-aminomethyltriazolyl)Agl^3^]-**1** (**2p**, 4 mg, 8% yield of >95% purity); LCMS 5–60%/10 min MeOH (0.1% FA) in water (0.1% FA), RT 7.9 min, and 5–60%/10 min MeCN (0.1% FA) in water (0.1% FA), RT 6.1 min.

#### [(3*R*,4*S*)-4-(4′-Hydroxymethyltriazolyl)Agl^3^]-1 (2q)


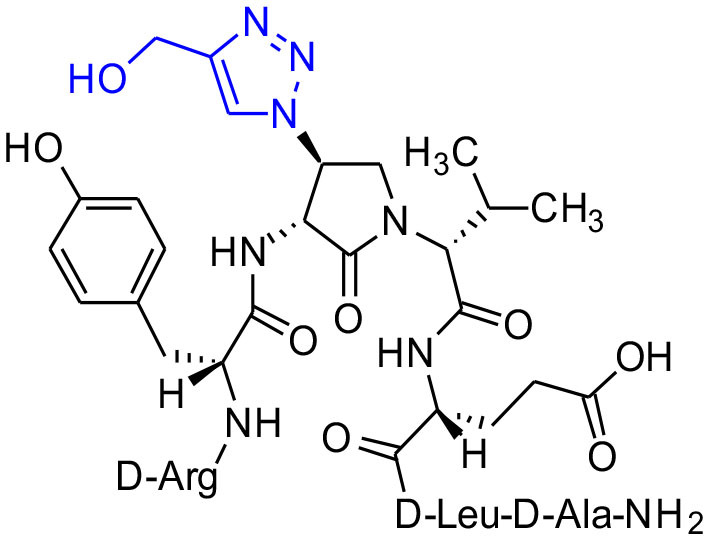
 Employing the representative procedures described for the synthesis of peptide **2l**, using resin **8c** (100 mg, 50.0 μmol) and propargyl alcohol (7 μL, 180 μmol), peptide **2q** was synthesized and indicated to be of 64% purity by LCMS analysis. Purification using method F with UV detection at 280 nm, and collection and freeze-drying of the pure fractions afforded [(3*R*,4*S*)-4-(4'-hydroxymethyltriazolyl)Agl^3^]-**1** (**2q**, 5 mg, 10% yield of >95% purity); LCMS 5–60%/10 min MeOH (0.1% FA) in water (0.1% FA), RT 7.2 min, and 5–60%/10 min MeCN (0.1% FA) in water (0.1% FA), RT 5.73 min; HRMS (ESI^+^) calcd m/z for C_41_H_64_N_14_O_11_ [M+H]^+^, 929.4930 found 929.4952.

#### Fmoc-(3*R*,4*S*)-β-azido-Agl-(*R*)-Val-OH (3c)


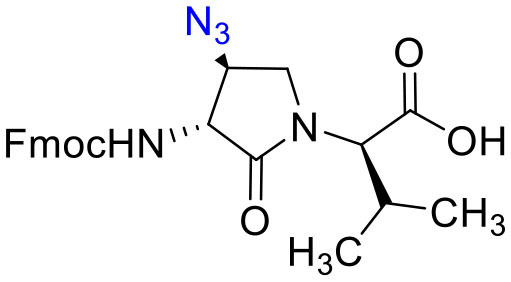
 Employing the procedure described below for the synthesis of Fmoc-(3*R*,4*S*)-β-phthalimidooxy-Agl-(*R*)-Val-OH (**3f**), Fmoc-(3*R*,4*S*)-β-azido-Agl-(*R*)-Val-O*t*-Bu (**5c**) (1 eq., 210 mg, 404 μmol, prepared according to Geranurimi and Lubell, 2018a) was converted to Fmoc-(3*R*,4*S*)-β-azido-Agl-(*R*)-Val-OH (**3c**, 155 mg, 83 %): R*f* = 0.07 (10% MeOH in DCM).

#### Fmoc-(3*S*,4*S*)-β-thiocyano-Agl-(*R*)-Val-OH (3d)


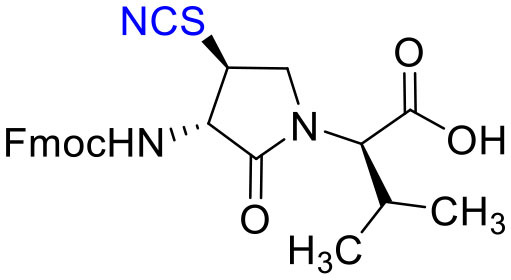
 Employing the representative procedure described below for the synthesis of Fmoc-(3*R*,4*S*)-β-phthalimidooxy-Agl-(*R*)-Val-OH (**3f**), Fmoc-(3*S*,4*S*)-β-thiocyano-Agl-(*R*)-Val-O*t*-Bu (**5d**, 1 eq., 55 mg, 103 μmol, prepared according to (Geranurimi and Lubell, 2018b)) was converted to Fmoc-(3*S*,4*S*)-β-thiocyano-Agl-(*R*)-Val-OH (**3d**, 45 mg, 91 %): R*f* = 0.1 (10% MeOH in DCM).

#### Fmoc-(3*S*,4*S*)-β-SMe-Agl-(*R*)-Val-OH (3e)


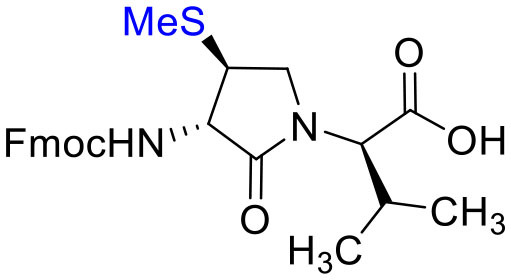
 Employing the representative procedure described below for the synthesis of Fmoc-(3*R*,4*S*)-β-phthalimidooxy-Agl-(*R*)-Val-OH (**3f**), Fmoc-(3*S*,4*S*)-β-SMe-Agl-(*R*)-Val-O*t*-Bu (**5e**, 1 eq., 27 mg, 51.4 μmol, prepared according to Geranurimi and Lubell, 2018b) was converted to Fmoc-(3*S*,4*S*)-β-SMe-Agl-(*R*)-Val-OH (**3e**, 19.0 mg, 79 %): R*f* = 0.09 (10% MeOH in DCM).

#### Fmoc-(3*R*,4*S*)-β-phthalimidooxy-Agl-(*R*)-Val-OH (3f)


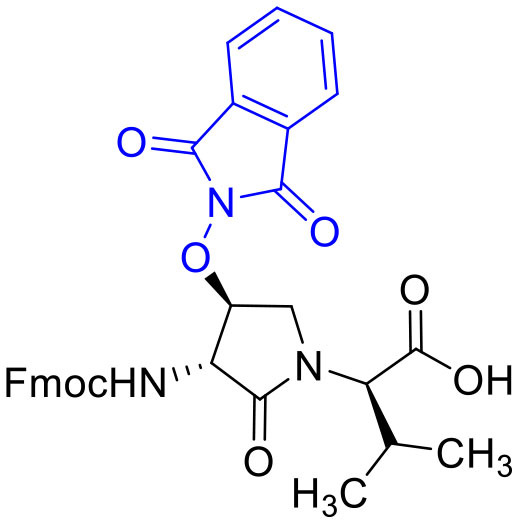
 A solution of Fmoc-(3*R*,4*S*)-β-phthalimidooxy-Agl-(*R*)-Val-O*t*-Bu (**5f**, 1eq., 32 mg, 50.0 μmol) in TFA (1 mL) and DCM (1 mL) was stirred at rt until TLC analysis revealed complete consumption of the ester. The volatiles were evaporated on a rotary evaporator. The residue was precipitated from ice-cooled diethyl ether and collected using a centrifuge to yield Fmoc-(3*R*,4*S*)-β-phthalimidooxy-Agl-(*R*)-Val-OH (**3f**, 26 mg, 89%), which was used without further purification: R*f* 0.06 (10% MeOH in DCM).

#### tert-Butyl (3*R*, 4*S*, 2'*R*)-2-[3-(Fmoc)amino-4-(1,3-dioxoisoindolin-2-yl)oxy)-2-oxopyrrolidin1-yl]-3-methylbutanoate (5f)


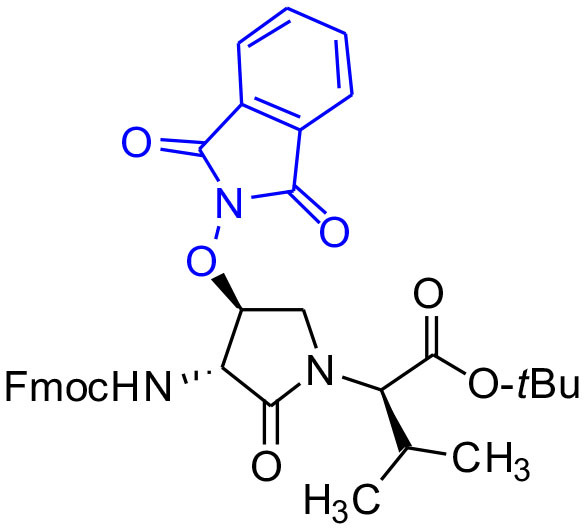
 A solution of sulfamidate **4** (1 eq., 80 mg, 144 μmol, prepared according to Geranurimi and Lubell, 2018a) in a mixture of DCM (2 mL) and DMF (1 mL) was treated with sodium *N*- hydroxyphthalimide (1 eq., 80 mg, 431 mmol), stirred at rt for 8 h, poured into 1 M NaH_2_PO_4_, and extracted with DCM. The combined organic phase was washed with brine, dried, filtered, and evaporated to a residue, that was purified by column chromatography using a step gradient of 20-30% EtOAc in hexane. Evaporation of the collected fractions provided (4*S*)-phthalimide **5f** (81 g, 78 %) as white foam: R_f_ = 0.37 (40% EtOAc in hexane); ${\left[\alpha \right]}_{\text{D}}^{25}$ 16.4° (*c* 1, CHCl_3_); ^1^H NMR (300 MHz, CDCl_3_) δ 7.88-7.65 (m, 8H), 7.41 (t, *J* = 7.1, 2H), 7.33 (t, *J* = 6.9, 2H), 6.53 (d, *J* = 8.6, 1H), 5.00 (t, *J* = 4.5, 1H), 4.77 (dd, *J* = 8.6, 5.1, 1H), 4.51-4.43 (m, 1H), 4.43-4.30 (m, 2H), 4.27-4.15 (m, 2H), 3.73 (dd, *J* = 12.5, 4.3, 1H), 2.29-2.11 (m, 1H), 1.41 (s, 9H), 1.02 (d, *J* = 6.6, 3H), 0.92 (d, *J* = 6.8, 3H); ^13^C NMR (75 MHz, CDCl_3_) δ 169.4, 168.9, 163.8, 156.7, 144.0, 143.8, 141.2, 134.7, 128.8, 127.7, 127.6, 127.1, 127.0, 125.6, 125.5, 123.8, 119.8, 82.1, 67.7, 61.0, 55.0, 47.4, 47.0, 28.7, 28.0, 21.9, 19.3; HRMS (ESI-TOF) m/z [M + H]^+^ calcd for C_36_H_38_N_3_${\text{O}}_{8}^{+}$ 640.2653, found 640.2624.

### qPCR Experiments

RAW Blue cells were purchased from InvivoGen (San Diego, CA), used at passages under 15, and cultured in DMEM growth medium supplemented with 10% serum, 50 U/mL penicillin and 200 mg/mL zeocin. Cells were grown in regular conditions (37°C, 5% CO_2_), serum starved overnight, and treated with 100 ng/mL IL-1β for 4 h. Cells were, respectively pre-incubated for 30 min with peptides **1** or **2** (10^−6^ M) or Kineret (1.0 mg/mL) to reach equilibrium prior to the experiments (*n* = 4 each treatment). Cells were harvested and incubated for 5 min in RIBOzol (AMRESCO). RNA was extracted according to manufacturer's protocol and RNA concentration and integrity were measured with a NanoDrop 1,000 spectrophotometer. A total of 500 ng of RNA was used to synthesize cDNA using iScript Reverse Transcription SuperMix (Bio-Rad, Hercules, CA). Primers (Table 1) were designed using National Center for Biotechnology Information Primer Blast. Quantitative gene expression analysis was performed using the Stratagene MXPro3000 (Stratagene) with SYBR Green Master Mix (Bio-Rad). Gene expression levels were normalized to 18S universal primer (Ambion Life Technology, Burlington ON, Canada). Genes analyzed include *IL1*β and *PTGHS2* [Prostaglandin H synthetase 2 or cyclooxygenase-2 (COX-2)]. Data are representative of 3 experiments (each with *n* = 4 per treatment group).

**Table 1:** List of primers for the human genes assessed by qPCR.

**Gene**	**Forward primer (5′ → 3′)**	**Reverse primer (5′ → 3′)**
*IL1β*	AGCTGGAGAGTGTAGATCCCAA	ACGGGCATGTTTTCTGCTTG
*PTGHS2*	ATATTGGTGACCCGTGGAGC	GTTCTCCGTACCTTCACCCC

### NF-kB QUANTI-Blue Assay

HEK-Blue IL-33/IL-1β cells (InvivoGen) were pretreated with peptides **1** or **2** (10^−6^ M), or Kineret (1.0 mg/mL) for 30 min, followed by treatment with a constant concentration of IL-1β (100 ng/mL), and incubation at 37°C for 4 h. Levels of secreted alkaline phosphatase in cell culture supernatant were determined using the QUANTI-Blue assay, according to the manufacturer's instructions (InvivoGen). Alkaline phosphatase activity was assessed by measuring optical density (OD) at 620–655 nm with an EnVision Multilabel micro plate reader (PerkinElmer, Waltham, MA). Data are representative of 3 experiments (each with *n* = 4 per treatment group).

### LPS-Induced Preterm Model in Mice

Timed-pregnant CD-1 mice at 16.5 days of gestation (G16.5) were anesthetized with 2% isoflurane and received an intraperitoneal injection of lipopolysaccharide (LPS, *n* = 4 per group, a single dose of 10 μg) (Kakinuma et al., 1997; Nadeau-Vallée et al., 2015). A dosage of 2 mg/kg/day of peptides **1** or **2** or vehicle was respectively injected subcutaneously in the neck, every 12 h until delivery. On G16.5, a dose of 1 mg/kg was injected 30 min before stimulation with LPS (to allow distribution of drugs to target tissues) and 1 mg/kg was injected 12 h after stimulation (*n* = 4 each treatment). Mice delivery was assessed every hour until term (G19–G19.5). A mouse was considered as delivering prematurely if the first pup was delivered earlier than G18.5. Data was analyzed using Prism 7 (GraphPad Software, San Diego, CA, USA) with one-way ANOVA and Dunnett's test for multiple comparisons. Outliers were detected using Grubb's test. Results were treated as significant when *p* was < 0.05 and expressed as mean ± SEM.

### Oxygen-Induced Retinopathy in Sprague Dawley Rats

Oxygen-induced retinopathy rodent model experiments were performed identically to those described in (Geranurimi et al., 2019), and described briefly below.

#### Animals

Two-day-old (P2) Sprague Dawley rat pups and their mothers were ordered from Charles River (Raleigh, SC, USA) and acclimatized for 3 days in standard conditions. All procedures and protocols involving the use of the rats were approved by the Animal Care Committee of the research center of Hôpital Maisonneuve-Rosemont and are in accordance with the Statement for the Use of Animals in Ophthalmic and Vision Research approved by the Association for Research in Vision and Ophthalmology, and guidelines established by the Canadian Council on Animal Care.

The 80% oxygen model of retinopathy was conducted as previously described (Geranurimi et al., 2019). Briefly, litters of P5 pups and their mothers were kept in a controlled 80% oxygen environment until P10. The pups were, respectively injected intraperitoneally twice daily with PBS vehicle (20 μL per injection), peptide **1**, or derivatives **2** (titrated to a daily dose of 2 mg/kg/day). Control litters were kept under normal air atmosphere and standard conditions. On P10, pups were euthanized by decapitation under 2% isoflurane anesthesia. Eyes were enucleated and fixed in 4% paraformaldehyde, then stored at 4°C in phosphate-buffered saline (PBS) until further processing.

#### Retinal Flatmount and Immunohistochemistry

The fixed eyes were dissected, and the obtained retinas were incubated with antibodies and mounted onto slides as previously described in Geranurimi et al. (2019). Briefly, the cornea and lens were removed from the eyes, and the retina gently removed from the underlying sclera-choroid-retinal pigmented epithelium (RPE) complex. Retinas were treated for 1 h with blocking solution (1% bovine serum albumin [BSA], 1% normal goat serum, 0.1% Triton X-100 and 0.05% Tween-20 in PBS), and then incubated overnight with lectin and Iba-1 primary antibody, followed by Alexa-594-conjugated secondary antibody for 2 h. Retinas were then mounted onto microscope slides under coverslips with anti-fade mounting medium.

#### Microscopy

Retinal flatmounts were imaged using the Zeiss AxioImager Z2 and the MosaiX feature of the AxioVision software as previously described (Geranurimi et al., 2019). Representative images after Iba-1 staining were taken using a laser scanning confocal microscope (Olympus IX81 with Fluoview FV1000 Scanhead) using the Fluoview Software at 30X magnification.

#### Quantification and Data Analysis

The FIJI software was used to quantify the area of vaso-obliteration in each retina, expressed as a percentage of the area of the whole retina. The number of Iba-1-positive cells was counted using the cell counter plug-in in the FIJI software, and the average of cell counts in 4 fields per retina was calculated. Data was analyzed using GraphPad Prism 7 with one-way ANOVA and the Dunnett's test for multiple comparisons. Results were treated as significant when *p* was less than 0.05 and expressed as mean ± SEM.

## Results

### Chemical Synthesis

**Figure 1:**
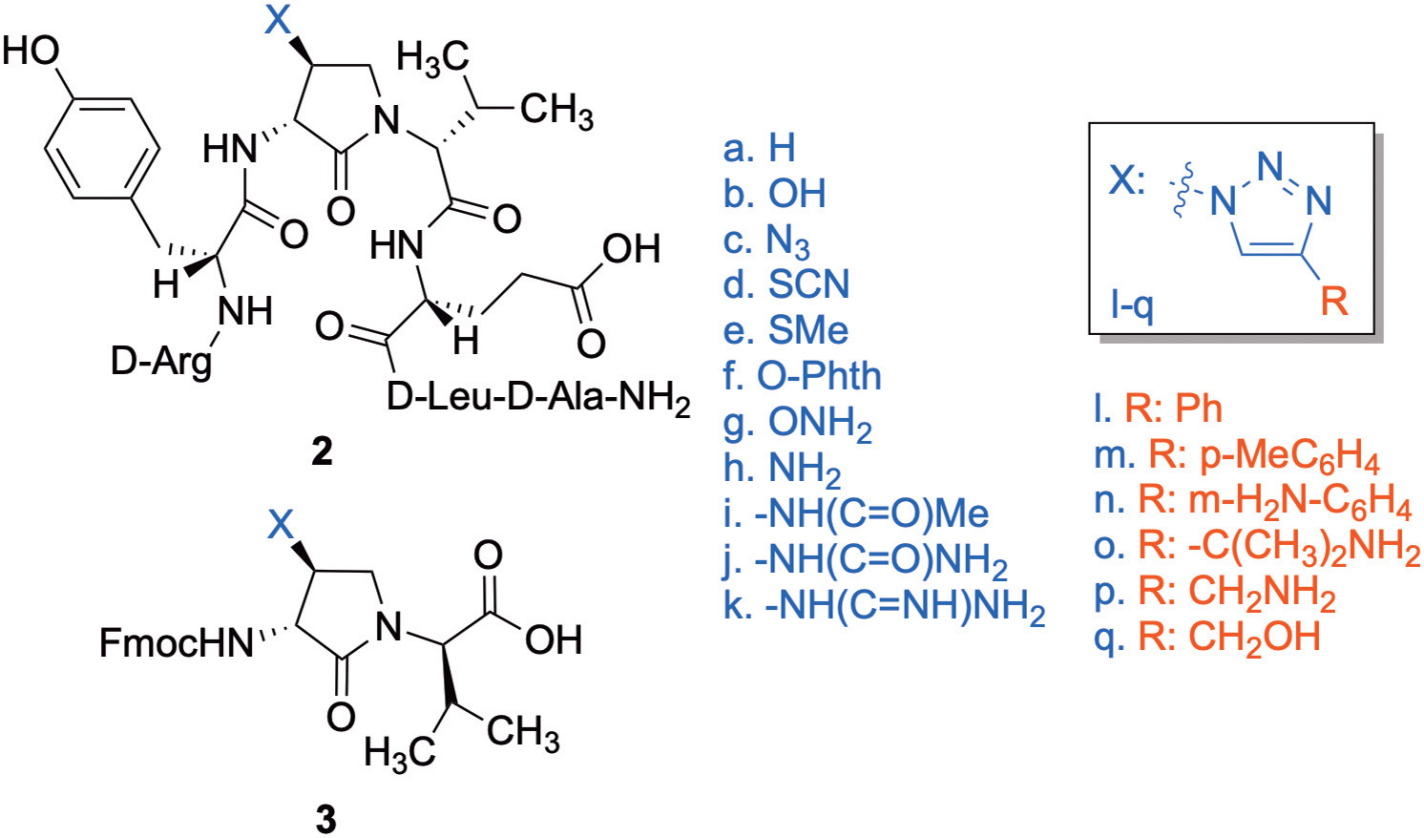
β-Substituted-Agl analogs **2** and **3**.

Cyclic sulfamidates are valuable intermediates for the synthesis of β-substituted amines (Meléndez and Lubell, 2003). Previously, sulfamidate **4** has been employed as a bis-electrophile to prepare β-substituted-Agl residues (Geranurimi and Lubell, 2018a). Nucleophilic ring opening reactions of sulfamidate **4** with sodium azide and potassium thiocyanate have served in routes to prepare Fmoc-(3*R*,4*S*)- and (3*S*,4*S*)-β-substituted-Agl-(*R*)-Val-OH analogs **3c-e** with β-azido, thiocyano and methylthio ether substituents, respectively (Figure 1) (Gulea et al., 2003; Geranurimi and Lubell, 2018a,b). The sodium salt of hydroxyphthalimide has now been used as nucleophile to open sulfamidate **4** in a 1:2 DMF/DCM mixture and provide Fmoc-(3*R*,4*S*)-β-phthalimidooxy-Agl-(*R*)-Val-O*t*-Bu (**5f**) in 78% yield (Scheme 1). *tert*-Butyl ester **5f** was converted to acid **3f** using a 1:1 trifluoroacetic acid/DCM solution.

**Scheme 2:**
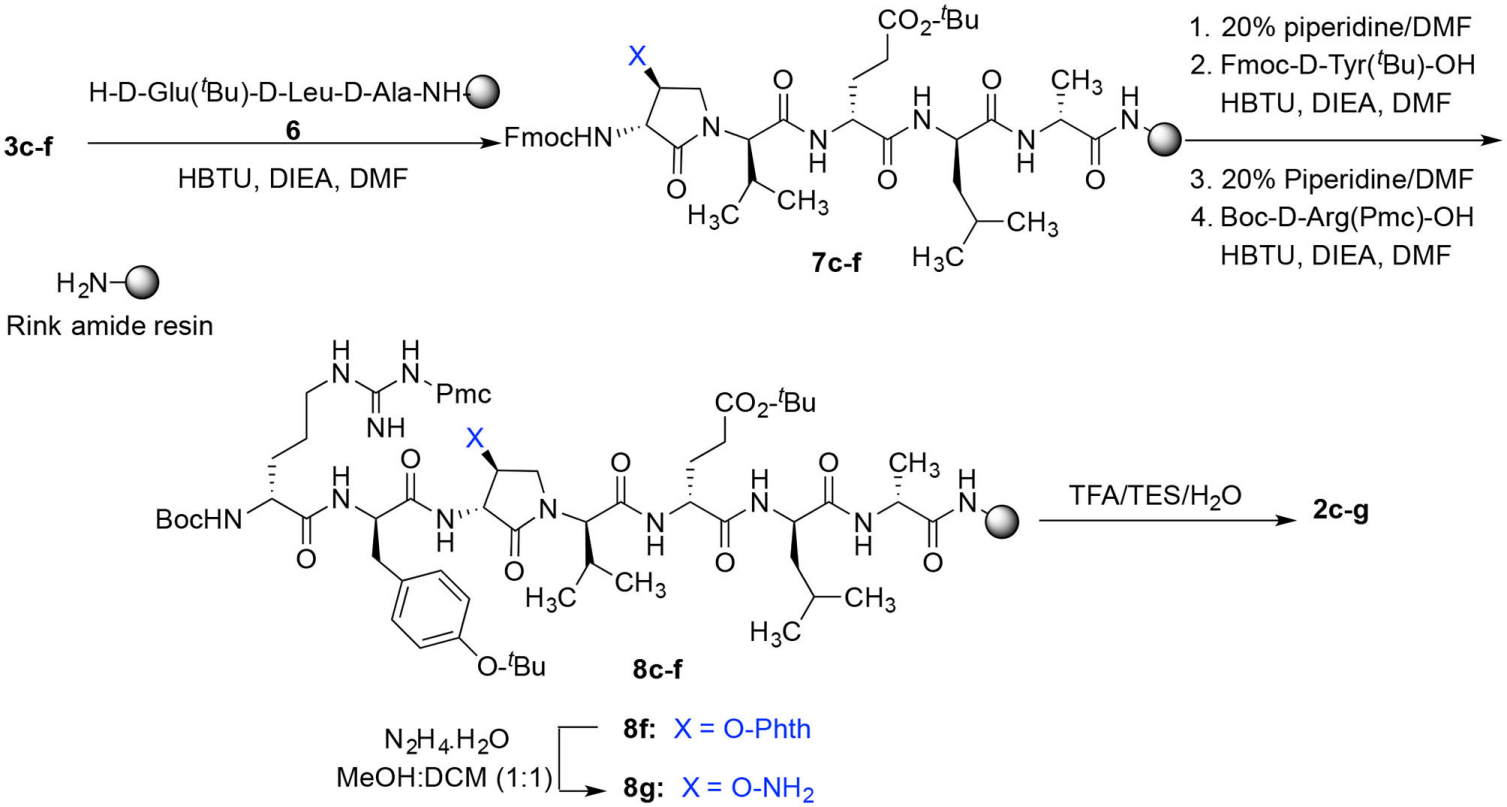
Solid-phase synthesis of peptides **2c-g**.

Dipeptide acids **3c-f** were respectively coupled to H-D-Glu(*t*-Bu)-D-Leu-D-Ala-NH-Rink amide resin **6** using *O*-(benzotriazol-1-yl)-*N*,*N*,*N*′,*N*′-tetramethyluronium hexafluorophosphate (HBTU), and *N*,*N*-diisopropylethylamine (DIEA) in DMF to provide pentapeptide resins **7c-f**. Peptide elongation by removals of Fmoc protection with 20% piperidine in DMF, and sequential couplings of Fmoc-D-Try(*t*-Bu)-OH and *N*-Boc-D-Arg(Pmc)-OH using HBTU and DIEA in DMF gave respectively protected heptapeptide resins **8c-f**. Treatment of *O*-alkyl hydroxy phthalimide resin **8f** with hydrazine monohydrate in a 1:1 MeOH/DCM mixture provided *O*-alkyl hydroxamine **8g** (Scheme 2) (Villadsen et al., 2017). Note, *O*-alkyl hydroxamine **8g** offers potential for the synthesis of oxime ligation conjugates (Guthrie and Proulx, 2018). Resin cleavage was performed using a cocktail of 95:2.5:2.5 TFA/H_2_O/TES to furnish peptides **2c-g** in 38–80% crude purities. Purification by HPLC provided peptides **2c-g** in 3–22% overall yields (Table 2).

**Table 2:** Retention times, purities, yields, and mass spectrometric data for peptides **2c-q**.

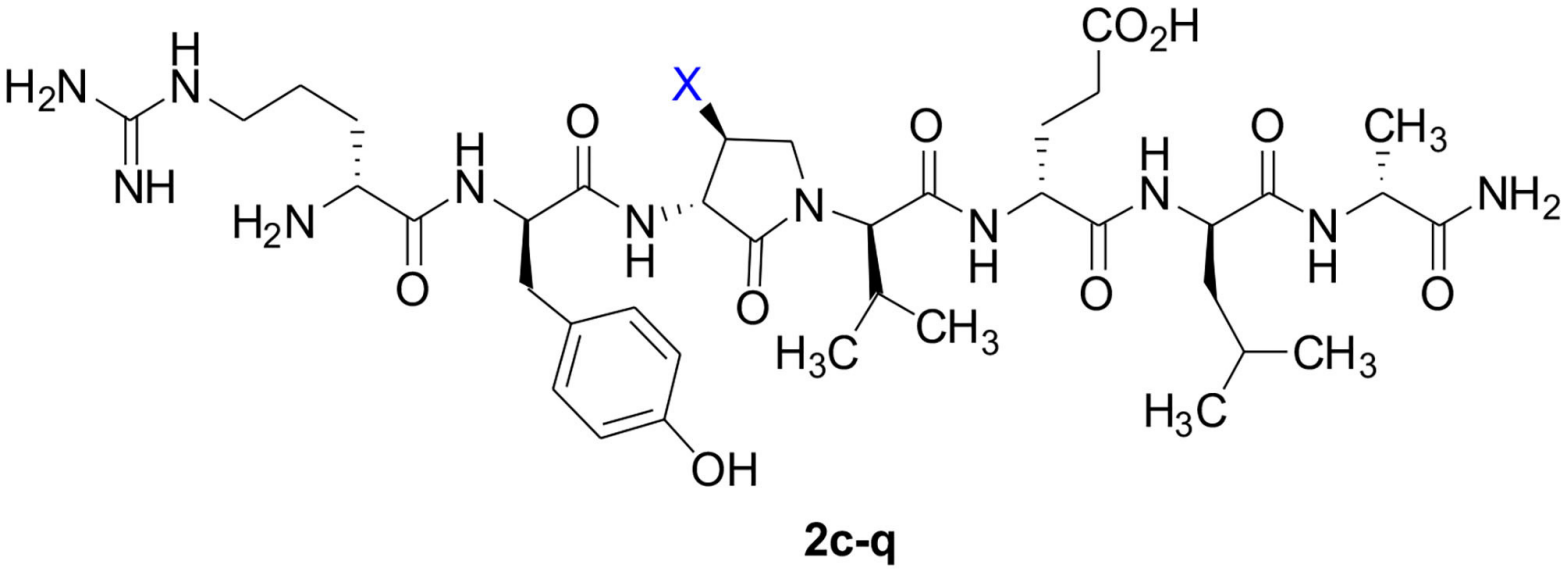								
**2**	-**X**	**RT (min)**		**Crude purity %**	**Final purity %**	**Yield % (>95 % in MeOH)**	**HRMS [M+1]**	
		**MeOH**	**MeCN**				**m/z (calcd)**	**m/z (obsd)**
**c**	-N_3_	8.3^a^	5.8^a^	80	>99	22	873.4690	873.4680
**d**	-SCN	8.3^a^	5.8^a^	38	>96	3	889.4349	889.4342
**e**	-SMe	8.7^a^	5.9^a^	46	>99	10	878.4553	878.4559
**f**	-OPhth	8.8^c^	7.3^d^	48	>96	11	993.4789	993.4786
**g**	-ONH_2_	6.6^a^	5.2^a^	38	>97	5	863.4661	863.4687
**h**	-NH_2_	5.8^a^	1.0^b^	70	>99	18	847.4785	847.4780
**i**	-NH(C=O)Me	7.5^a^	5.9^a^	41	>99	9	890.4850	890.4867
**j**	-NH(C=O)NH_2_	7.3^a^	5.1^a^	57	>99	8	890.4843	890.4841
**k**	-NH(C=N)NH_2_	5.9^a^	4.6^d^	62	>98	9	889.5003	889.5006
**l**	−4-(Ph)triazolyl	9.6^a^	6.5^a^	74	>99	17	975.5159	975.5147
**m**	−4-(*p*-MePh)triazolyl	9.5^e^	6.5^f^	71	>97	16	989.5316	989.5307
**n**	−4-(*m*-H_2_NPh)triazolyl	7.9^d^	6.1^d^	69	>96	12	990.5268	990.5259
**o**	−4-(H_2_N(Me)_2_C)triazolyl	6.3^d^	4.9^d^	73	>98	13	956.5425	956.5408
**p**	−4-(H_2_NH_2_C)triazolyl	7.9^d^	6.1^d^	53	>97	8	928.5039	928.5029
**q**	−4-(HOH_2_C)triazolyl	7.2^d^	5.7^d^	64	>97	10	929.4930	929.4952

*Isolated purity ascertained by LC-MS analysis using gradients X-Y% MeOH (0.1% FA) or MeCN (0.1% FA) in H_2_O (0.1% FA) over 10 min*.

a) *10–90%*,

b) *50–90%*,

c) *30–60%*,

d) *5–60%*,

e) *30–90%*,

f) *20–40%*.

**Scheme 3:**
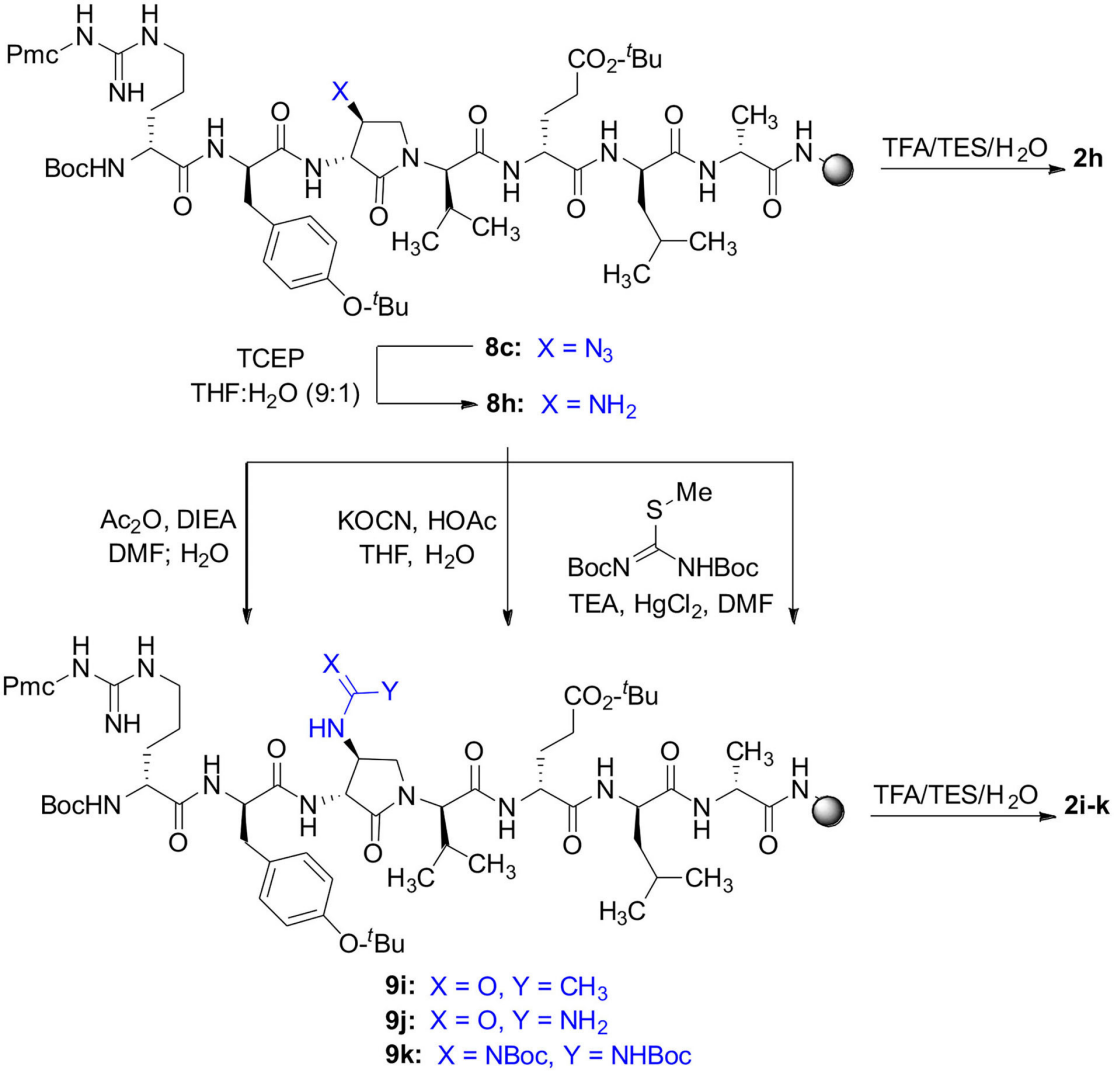
Solid-phase synthesis of peptides **2h-k**.

Amine resin **8h** was synthesized by reduction of azide **8c** using tris-(2-carboxyethyl)phosphine hydrochloride (TCEP) in a 9:1 THF:H_2_O mixture (Scheme 3). Amine **8h** was then employed in the synthesis of acetamide, urea and guanidine peptides **8i-k**. Acetamide **8i** was prepared by acylation of amine **8h** using acetic anhydride and DIEA in DMF. Urea **8j** was obtained from treating amine **8h** with a solution of potassium cyanate and acetic acid in a 20:1 THF:H_2_O mixture (Wertheim, 1931). Guanidine **9k** was prepared by reacting amine resin **8h** with 1,3-bis(*tert*-butoxycarbonyl)-2-methyl-2-thiopseudourea, triethylamine and mercuric chloride in DMF (Dianati et al., 2017). Resin cleavage and removal of the Boc and *tert*-butyl protection groups were concomitantly accomplished using a cocktail of 95:2.5:2.5 TFA/H_2_O/TES to furnish peptides **2h-k** in 41–70% crude purities. Purification by HPLC provided peptides **2h-k** in 8–18% overall yields (Table 2).

**Scheme 4:**
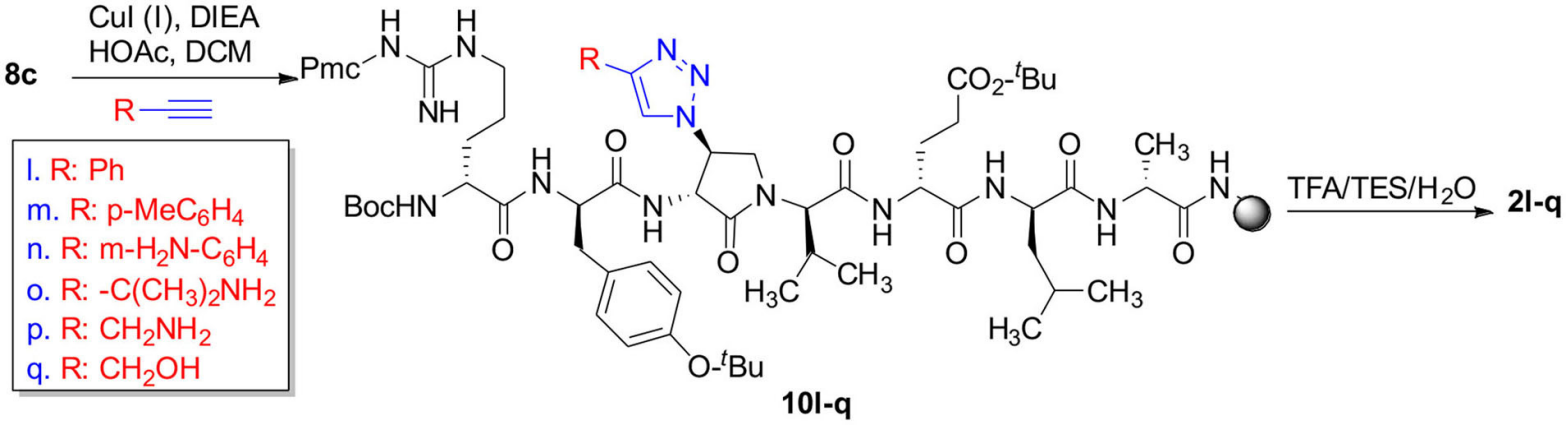
CuAAC chemistry on resin **8c** provides access to triazole derivatives **2l-q**.

Azide **8c** was also employed in CuAAC chemistry to provide 4-substituted-1,2,3-triazoles **8l-q** using a set of alkynes: phenylacetylene, 4-ethynyltoluene, 3-ethynylaniline, 1,1-dimethylpropargylamine, propargylamine and propargylalcohol (Scheme 4). Alkynes were selected to study potential for aromatic, salt bridge and hydrogen bond interactions with the receptor. In the CuAAC reaction, azide **8c** was treated with the corresponding alkyne, copper(I) iodide and DIEA in DCM to provide a single regioisomer, 4-substituted-1,2,3-triazoles **8l-q** (Shao et al., 2011). The regioselectivity of the cyclization reaction to form **8l-q** was inferred from spectral analysis of Fmoc-(3*R*,4*R*)-4-(4'-phenyltriazolyl)Agl-(*R*)-Val-O*t*-Bu, which was synthesized from azide (3*R*, 4*R*, 2'*R*)-**5c** using identical CuAAC conditions in solution, and showed a ^13^C signal at 120 ppm and no signal at 133 ppm indicative of a 4-substituted triazole. As described above, resin cleavage and removal of protection were concomitantly accomplished using a TFA/H_2_O/TES cocktail to furnish peptides **2l-q** in 53–74% crude purities. After purification by HPLC, peptides **2l-q** were isolated in 8–17% overall yields (Table 2).

### Circular Dichroism Spectra

**Figure 2:**
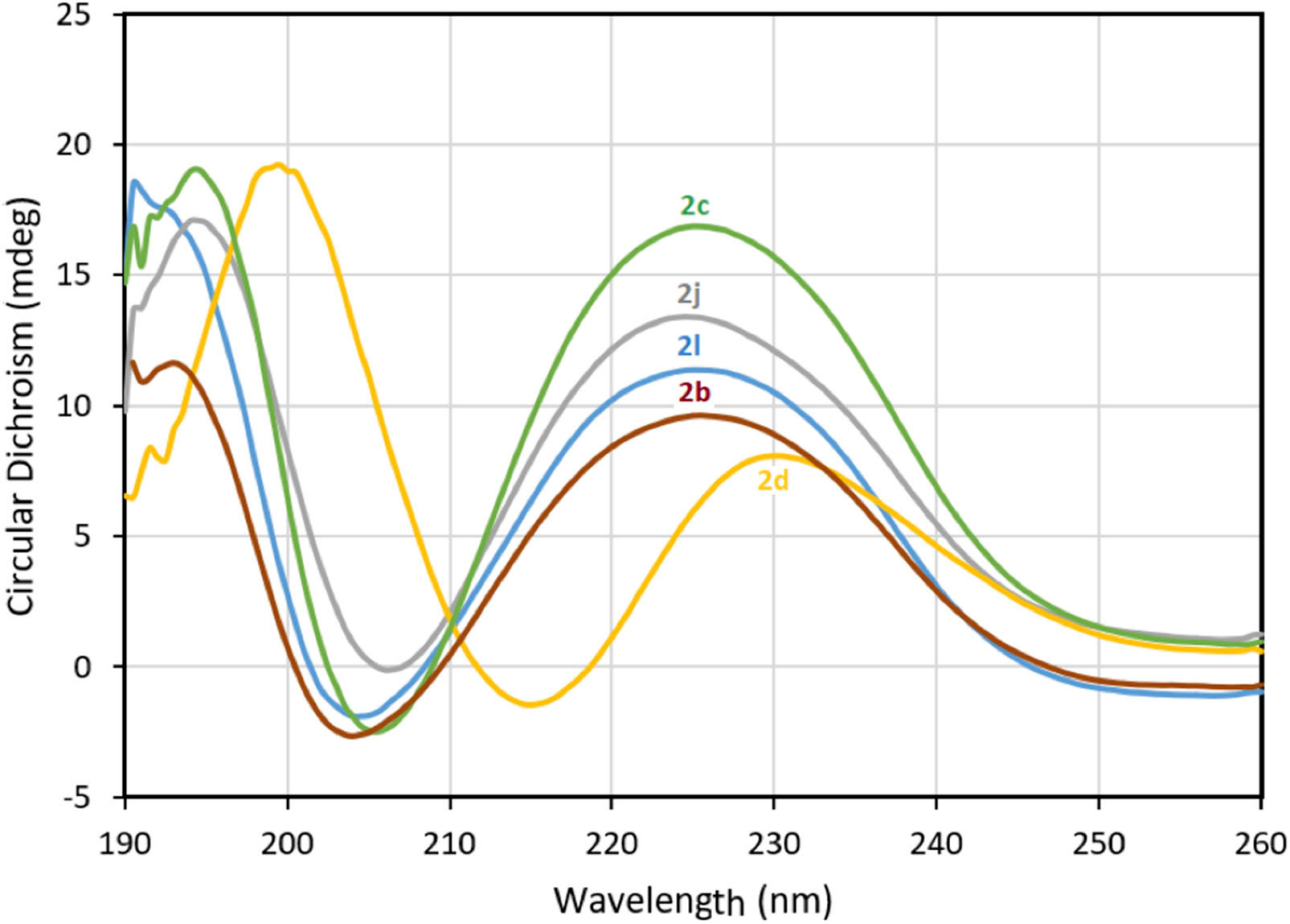
The molar ellipticity circular dichroism spectra of **2b-d**, **2j**, and **2l**.

The impact of the β-substituent on the conformation of (3*R*,4*S*)-β-substituted-Agl^3^ peptides **2c-q** was examined in water by CD spectroscopy and the curve shapes of the spectra were compared with that of [(3*R*,4*S*)-Hgl^3^]-**1** (**2b**). Previously, **2b** exhibited negative and positive maximum, that were respectively at 198–207 and 221–227 nm indicative of a β-turn conformation in water, trifluoroethanol (TFE), MeOH and hexafluoroisopropanol (HFIP), with the greatest ellipticity seen in 5% TFE in water. In general, peptides **2** exhibited curve shapes indicative of β-turn conformers with slightly different ellipticities (Figure 2 and Supporting Information). Notably, thiocyanate **2d** exhibited a similar curve shifted to higher wavelengths at 215 and 230 nm (Figure 2). On the other hand, no curve shape was obtained from measuring the CD spectrum of 4-hydroxymethyltriazolyl peptide **2q** (Supporting Information). The similar curve shapes illustrated in the spectra of peptides **2b-p** indicated that changes of the β-substituent had little influence on the peptide conformation, which was previously shown to be significantly affected by the presence and configuration of the γ-lactam ring (Geranurimi et al., 2019).

### Biology

**Figure 3:**
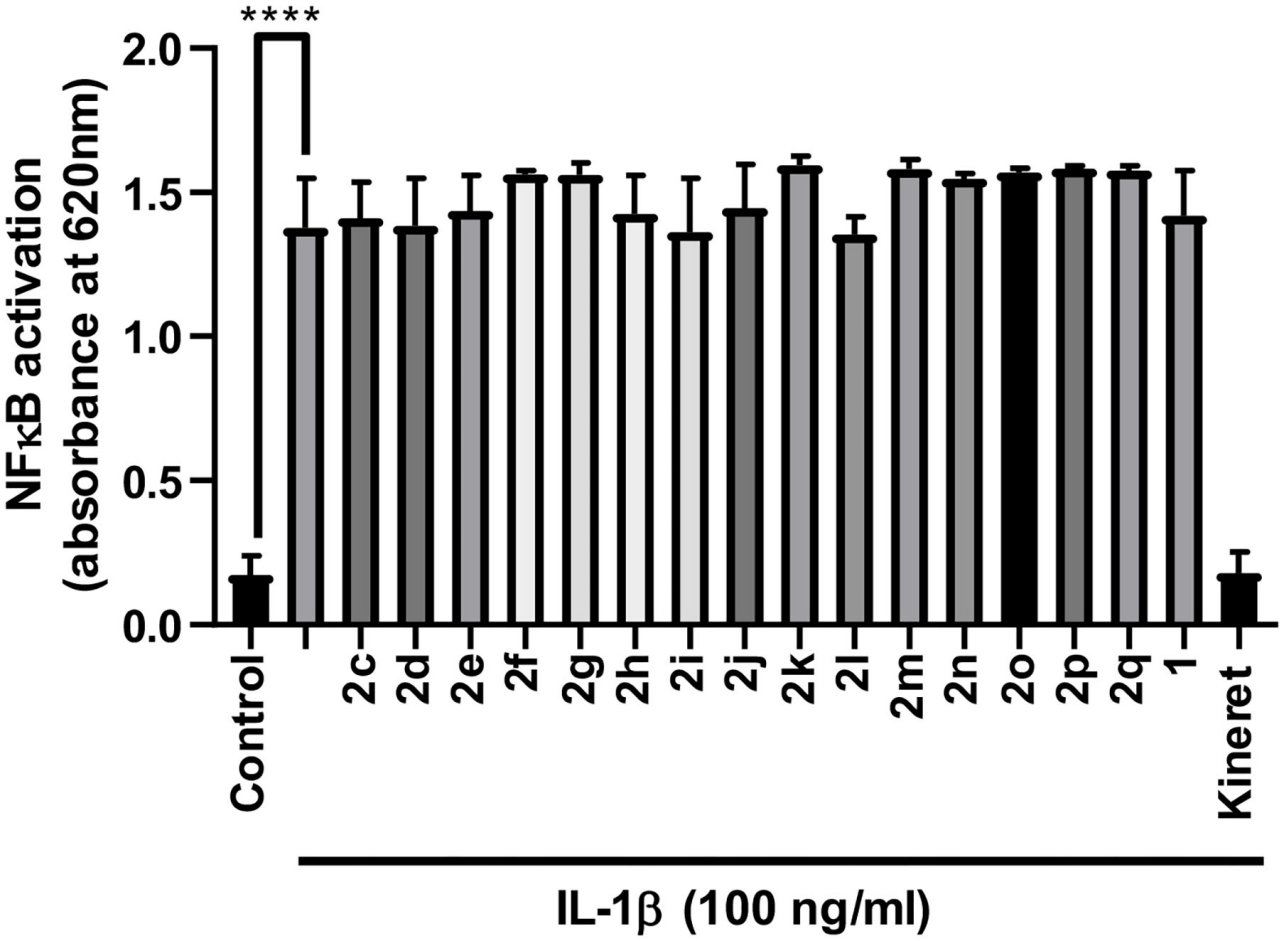
Effects of peptides **1** and **2** on IL-1β-induced NF-κB signaling in HEK-Blue cells as quantified in the QUANTI-blue assay. After pre-incubation with peptide or vehicle, HEK-Blue cells were stimulated with IL-1β for 24 h, and secreted alkaline phosphatase activity was spectroscopically detected as a reporter product from the transcription of the NF-κB gene. Data shown represents the average of 2 experiments (each with *n* = 4 per treatment group): ****p < 0.0001 compared to group treated only with IL-1β.

Anti-inflammatory agents that modulate the IL-1R but preserve NF-κB signaling are desired to avoid compromising immune vigilance against invading pathogens. Peptides **2c-q** were examined for their effects on the NF-κB pathway using a reported assay that had previously been shown to contrast the immunosuppressive activity of Kineret with that of peptide **1** (Nadeau-Vallée et al., 2015). The effects of peptides **1** and **2c-q** on the activation of NF-κB signaling by IL-1β was assessed using the QUANTI-blue assay, which quantifies the secretion of alkaline phosphatase, a reporter gene product for NF-κB. Peptides **2c-q** all behaved like **1** in the assay and exhibited no effect on IL-1β-induced NF-κB signaling (Figure 3). On the other hand, Kineret blocked NF-κB signaling in line with the activity of an orthosteric antagonist.

**Figure 4:**
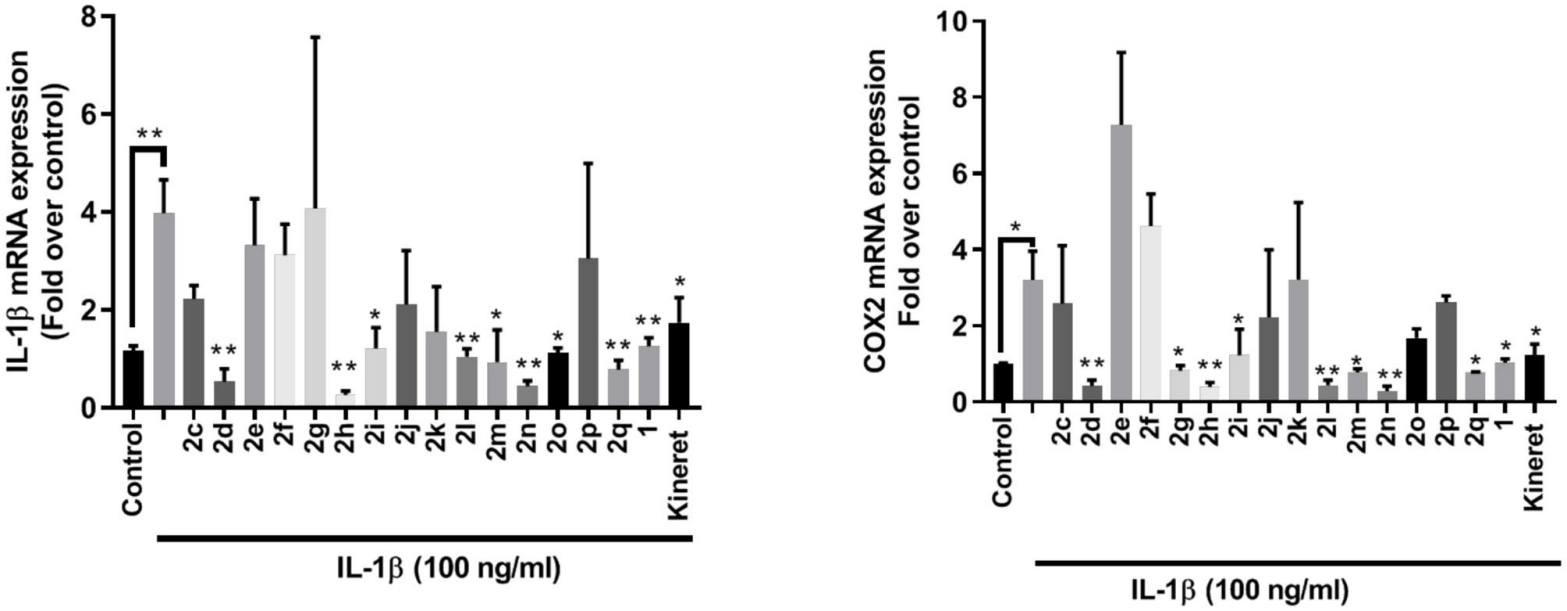
qPCR analysis of IL-1β (left) and COX-2 (right) gene expression within lysates from RAW 264.7 mouse macrophage cells after exposure to peptides **1** and **2** (10^−6^ M) or Kineret (1 mg/mL) followed by IL-1β incubation overnight. Results represent an average of 3 independent experiments (each with *n* = 4 per treatment group) and are expressed as a fold-change of the non-stimulated control: *p < 0.05, **p < 0.01 compared to group treated only with IL-1β, and 18S rRNA as internal control. Treatment groups that are not labeled with asterisks are statistically non-significant compared to group treated only with IL-1β.

The effects of peptides **1** and **2** on the expression of pro-inflammatory genes for IL-1β and COX-2 were examined *in vitro* in 264.7 RAW mouse macrophages. Four peptides exhibited statistically (^**^p < 0.01) similar or better activity in reducing pro-inflammatory gene expression than peptide **1** and Kineret: thiocyanate **2d**, amine **2h**, 4-phenyltriazole **2l** and 4-*m*-aminophenyltriazole **2n** (Figure 4). Another three (**2o**, **2m**, and **2q**) had strong inhibitory effects with lower statistical relevance (^*^p < 0.05) while **2g** and **2i** blocked expression of COX-2 (^*^p < 0.05) without effecting IL-1β expression.

**Figure 5:**
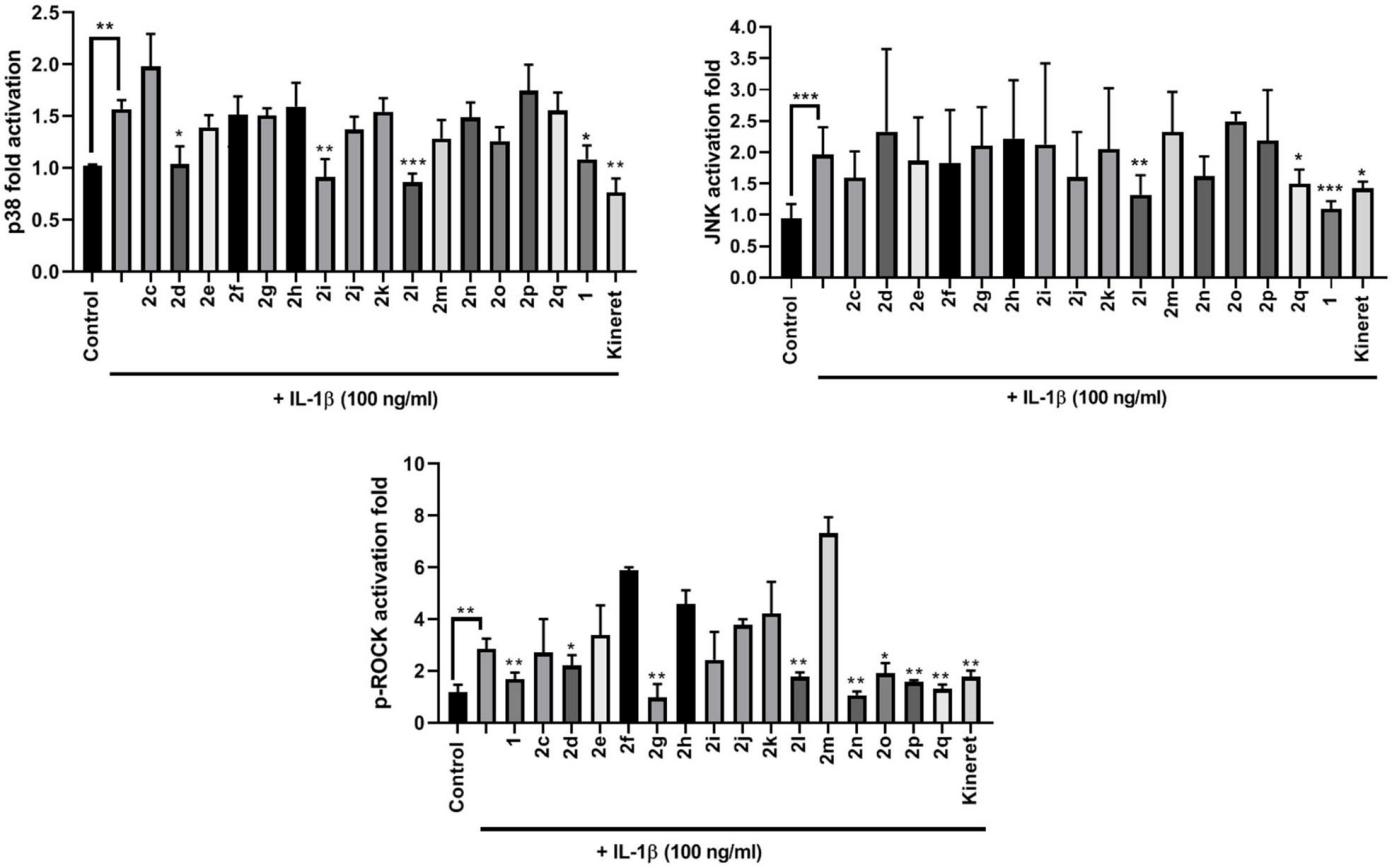
The effects of peptides **1** and **2** on IL-1β-induced phosphorylation of p38 (upper left panel), JNK (upper right panel), and ROCK2 (lower middle panel). Graphical representations of band density analysis of Western Blots are shown as fold activation compared to control. RAW Blue cells were pretreated with peptides **1** and **2** (10^−6^ M), Kineret (1 mg/mL), or vehicle for 30 min and then stimulated with IL-1β for 15 min. Images of representative Western Blots can be found in the Supplementary Figure 1. Results shown are the average of 3 independent experiments: *p < 0.05, **p < 0.01, ***p < 0.001 compared to group treated only with IL-1β. Treatment groups that are not labeled with asterisks are statistically non-significant compared to group treated only with IL-1β.

The modulatory effects of peptides **1** and **2** on IL-1β-induced kinase phosphorylation were also determined (Figure 5). Thiocyanate **2d**, amine **2i**, and phenyltriazol **2l** were the only peptides inhibiting p38 phosphorylation. Meanwhile, only **2l** and **2q** inhibited activation of JNK. Many β-substituted Agl analogs **2** exhibited inhibitory activity on ROCK2 phosphorylation (e.g., **2g** and **2l**). All triazolyl analogs **2l-q**, except for 4-(*p*-tolyl)triazole **2m**, strongly inhibited ROCK2 phosphorylation activity.

**Figure 6:**
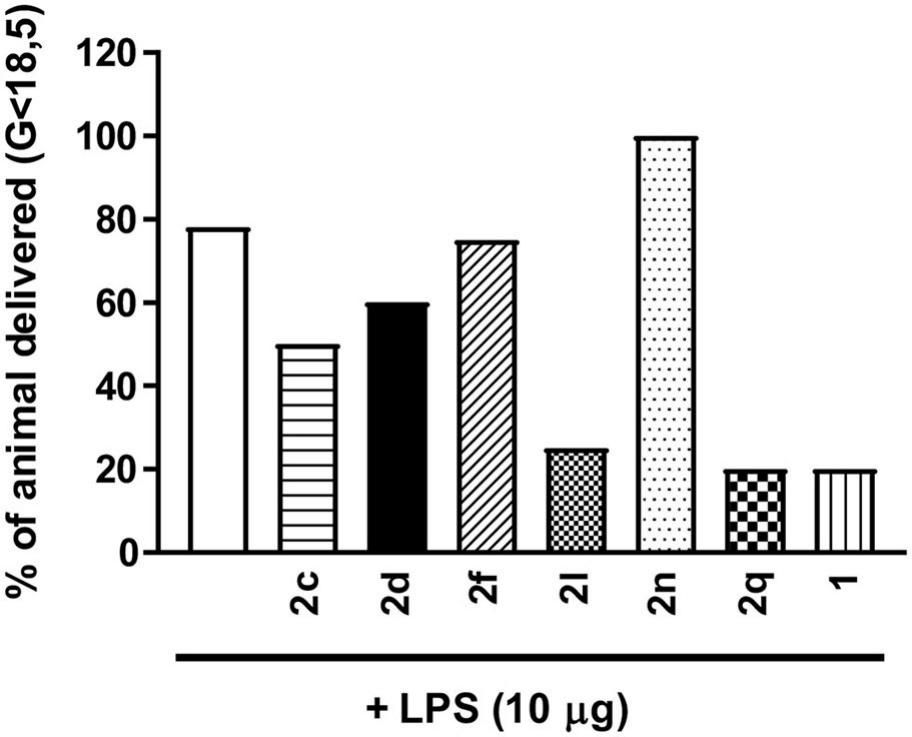
The effects of peptides **1** and **2** on prevention of PTB. In brief, pregnant dams on day 16.5 of gestation (G16.5) were subcutaneously pretreated with peptides **1** and **2** (2 mg/kg/day subcutaneous injections) or vehicle, followed by LPS (10 μg intraperitoneal injection), and observed for delivery of pups. A dam was considered as delivering preterm if at least one pup was delivered before G18.5. *n* = 4–5 dams per treatment group.

From the results of the *in vitro* screens, a subset of six (3*R*,4*S*)-β-substituted-Agl^3^ peptides (**2c**, **2d**, **2f**, **2l**, **2n**, **2q**) were selected for examination *in vivo* in a CD-1 mouse model of preterm birth (PTB), and a Sprague Dawley rat model of oxygen-induced retinopathy (OIR). In the PTB model, timed-pregnant CD-1 dams were pre-treated with peptides **1** or **2**, or PBS vehicle, and then injected with LPS on day 16.5 of gestation (G16.5). LPS, a bacterial cell wall component that contains PAMPs, is known to reliably induce labor via pro-inflammatory pathways implicating IL-1 (Hirsch and Wang, 2005). The use of LPS may better mimic more systemic endotoxemia instead of the more commonly observed intrauterine bacterial infection of spontaneous PTB in human cases. The same inflammatory responses evoked by viable Gram-negative bacteria are however elicited by LPS (Huang et al., 1999), including the upregulated expression of COX-2 observed in human placental tissues (Gross et al., 2000). The CD-1 mice have a mean gestation of 19.2 days (Goupil et al., 2010). Dams that delivered at least one pup before G18.5 were considered to have given birth prematurely. In the absence of peptide, LPS alone induced premature labor in ~80% of treated mice (Figure 6). Peptide **1** and triazoles **2l** and **2q**, all reduced the PTB rate to ~20%. Azide **2c** and thiocyanate **2d** both exhibited modest effects by reducing the PTB rate to 40–50%. Neither *N-*oxyphthalimide **2f** nor 4-*m*-aminophenyltriazole **2n** exhibited effects on PTB.

**Figure 7:**
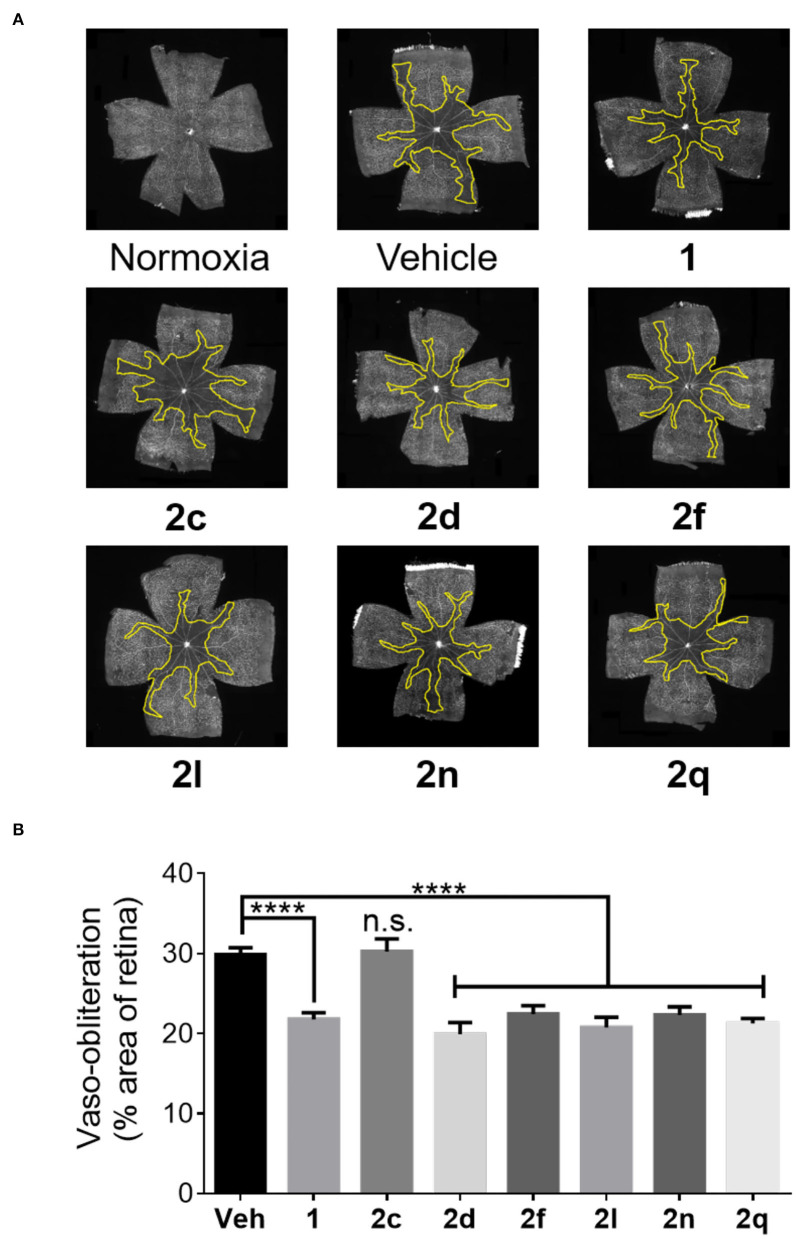
The preventive effects of peptides **1** and **2** against vaso-obliteration in an OIR model. **(A)** Representative retinal flatmounts stained with FITC-conjugated *Bandeiraea simplicifolia* lectin at 10X magnification. Yellow lines indicate the central area of vaso-obliteration extending from the optic nerve in the center of the retina. **(B)** Quantification of area of vaso-obliteration performed using ImageJ, expressed as a percentage of the total retinal area: *n* = 5–7 of peptide **2**, *n* = 10–12 for vehicle and peptide **1**; Veh vehicle; ****p < 0.0001 relative to the vehicle group, n.s. *p* > 0.05 relative to vehicle group and not statistically significant.

The efficacies of peptides **1** and **2** were compared in the well-established OIR model in Sprague Dawley rats as previously described (Geranurimi et al., 2019). After birth, rat pups were exposed to 80% oxygen from days 5 to 10, which usually resulted in ~30% vaso-obliteration of the retinal capillaries that extend radially from the optic nerve (vehicle, Figures 7A,B). As previously reported (Geranurimi et al., 2019), peptide **1** diminished the extent of vaso-obliteration from ~30% to ~20% (*p* < 0.0001). Five of the six peptides examined, thiocyanate **2d**, *N-*oxyphthalimide **2f**, 4-phenyltriazole **2l**, 4-*m*-aminophenyltriazole **2n**, and 4-hydroxymethyltriazole **2q**, exhibited efficacy in the OIR model and reduced vaso-obliteration from ~30% to ~20% (*p* < 0.0001). Although thiocyanate **2d** and 4-phenyltriazole **2l** exhibited tendencies to have a stronger protective effect than peptide **1**, there was no statistically significant difference between the three peptides. On the other hand, [(3*R*, 4*S*)-4-(N_3_)Agl^3^]-**1** (**2c**) had no protective effect against vaso-obliteration and was indistinguishable from the vehicle-treated group.

**Figure 8:**
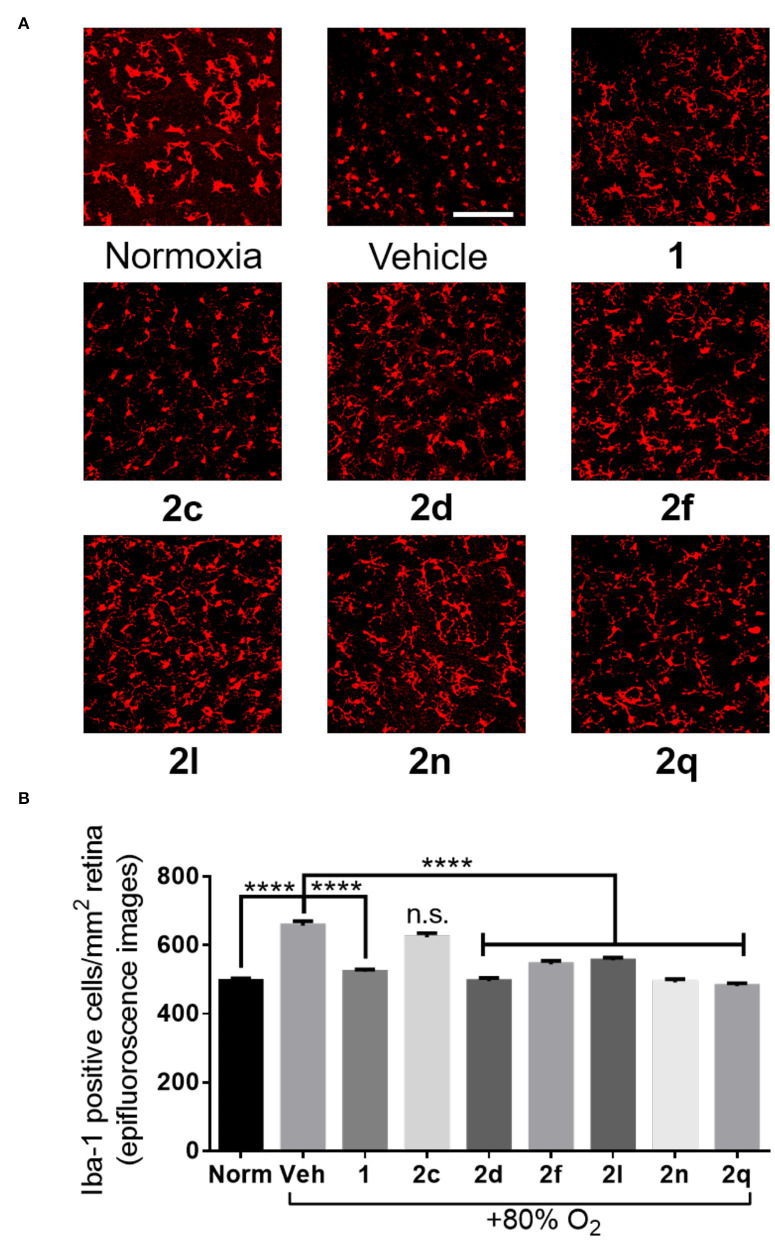
The effects of peptides **1** and **2** on retinal microglial activation and density. **(A)** Representative confocal images of retinal microglia at 30X magnification: scale bar 100 μm. **(B)** Epifluorescence microscopy images at 20X magnification of retinal microglial density quantified using ImageJ: 4 images per retina were taken at a distance halfway between the optic nerve and the edge of the retina; *n* = 5–7 for peptides **1** and **2**; *n* = 10–12 for normoxia and vehicle; Norm normoxia, Veh vehicle; ****p < 0.0001 relative to the vehicle group, n.s. *p* > 0.05 relative to vehicle group and not statistically significant.

In the context of OIR, microglia have been previously shown to be mediators of vaso-obliteration (Rivera et al., 2013). The ramified and branched morphology of inactive microglia has also been observed to change to an amoeboid state with retracted limbs upon microglial activation (Donat et al., 2017). Microglial activation and density were thus ascertained by histochemical staining for the Iba-1 marker. Microglia in the active amoeboid state were observed in the retina of animals kept under hyperoxia and treated with vehicle or azide **2c** (Figures 8A,B). Conversely, pups raised in normoxia exhibited ramified and branched microglia in their retina. Furthermore, pups presented retina with similarly ramified and branched microglia after treatment with peptides **1** and **2** (e.g., **2d**, **2f**, **2l**, **2n**, and **2q**) under hyperoxia, which exhibited diminishing effects on vaso-obliteration. In summary, five of the six tested (3*R*,4*S*)-β-substituted-Agl^3^ peptides acted like peptide **1** and exhibited protection against vaso-obliteration in the hyperoxic phase of OIR, due in part to attenuation of microglial activation.

## Discussion

The relevance of the β-substituent for activity are revealed in a comparison of the results of the *in vitro* and *in vivo* experiments on (3*R*,4*S*)-β-substituted-Agl^3^ peptides **2c-q** and parent peptide **1** (Table 3). Comparison of the curve shapes of the CD spectra of the (3*R*,4*S*)-β-substituted-Agl^3^ peptides **2c-q** indicated that the β-turn conformation exhibited in [(3*R*,4*S*)-Hgl^3^]-**1** (**2b**) was maintained in most of the analogs. Moreover, peptides **1** and **2**, all were typically pathway selective and showed no effects on NF-κB signaling, signifying retained immune vigilance. Inhibition of at least one of the three kinase pathways has been usually found to be necessary for exhibiting *in vivo* effects in the PTB and ROP models (Geranurimi et al., 2019). However, *N-*oxyphthalimide **2f** did not inhibit IL-1 induced expression of COX2 and IL-1 nor kinase phosphorylation, but prevented vaso-obliteration and microglial activation in the OIR model, which indicates the likelihood of another pathway among the pleiotropic effects of IL-1 being implicated in the pathogenesis of OIR and ROP (Mantovani et al., 2019).

**Table 3:**
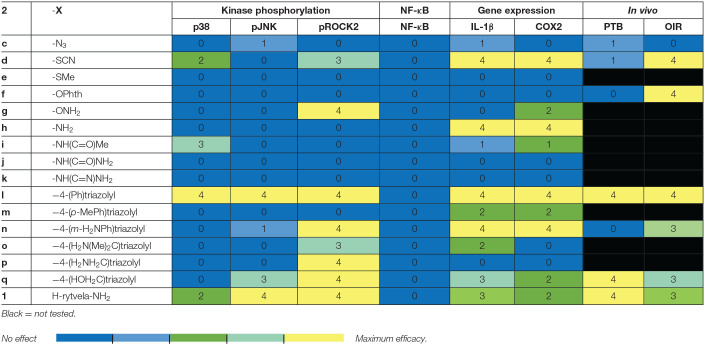
Heatmap summary of the *in vitro* and *in vivo* effects of peptides **1** and **2**.

The (3*R*,4*S*)-β-Substituted-Agl^3^ analogs **2c-q** provide means of studying the role of the β-substituent for mediating anti-inflammatory effects within a conformationally constrained variant of peptide **1**. Studying the conformation of the Thr^3^ residue in **1**, β-hydroxy-α-amino-γ-lactam [(3*R*,4*S*)-Hgl^3^]-**1** (**2b**) was shown to exhibit identical *in vitro* and *in vivo* activity as the parent peptide. The β-substituent was changed to other groups that could in principle interact in hydrogen bonds, salt bridges and π-cation interactions. Switching the alcohol to various groups which could serve as alternative hydrogen-bond donors and acceptors and engage in salt bridges (e.g., **2d**-**2k**) caused typically significant losses in activity in the three kinase pathways with notable exceptions of thiocyanate **2d** (some activity on p38 and ROCK2), amide **2i** (some activity on p38), and alkoxyamine (strong activity on ROCK2). Notably, thiocyanate **2d** and amine **2h** exhibited statistically better activity in reducing pro-inflammatory IL-1β and COX-2 gene expression in mouse macrophages compared to peptide **1**.

In the case of the triazole analogs (i.e., **2l-q**), except for 4-(*p*-tolyl)triazole **2m**, all demonstrated inhibitory activity on the ROCK2 pathway. Moreover, 4-(phenyl)triazole **2l** exhibited strong inhibitory activity on the p38 and JNK pathways. The 4-hydroxymethyl counterpart **2q** also exhibited inhibitory activity on the JNK pathway. 4-(Phenyl)triazole **2l** and 4-(*m*-aminophenyl)triazole **2n**, both reduced significantly pro-inflammatory gene expression for IL-1β and COX-2. Similar to alcohol **2b**, triazole peptides **2l-q** offer potential to participate in hydrogen bonds (Agalave et al., 2011). Moreover, the triazoles have a variety of 4-position substituents, such as aromatic, amine and alcohol functions, that may influence receptor interaction.

Previously, inhibitors of JNK and ROCK2 have been respectively, shown to delay labor and reduce neovascularization in models of PTB and OIR (Pirianov et al., 2015; Yamaguchi et al., 2016). Consistent with such findings, peptides **2** exhibiting inhibitory activities on the JNK (**2l** and **2q**) and ROCK2 (**2d**, **2l**, **2n** and **2q**) pathways demonstrated efficacy in the PTB and OIR models *in vivo*, respectively. The battery of *in vitro* examinations used in this study has further validated the importance of such pathways for *in vivo* activity; however, the capacity of *N-*oxyphthalimide **2f** to reduce vaso-obliteration and microglial activation in the OIR model without effects on kinase phosphorylation and gene expression demonstrates the probability that other IL-1R mediated pathways may be valid targets for the indication of ROP.

The potential influences of pharmacokinetic properties of peptides **2c-q** must be considered in the examination of structure-activity relationships of the *in vitro* and *in vivo* experiments, because biological distribution and metabolism may influence potency. Nevertheless, with relatively similar or better activity than parent peptide **1**, 4-(phenyl)triazole **2l** has exhibited notable inhibition of phosphorylation of the three kinases and of gene expression of IL-1β and COX-2, as well as potency in both *in vivo* models. In contrast, the absence of activity on kinase phosphorylation and the weak inhibitory effects on gene expression of 4-(*p*-tolyl)triazole **2m** indicate a detrimental steric effect of the *p*-methyl group. Alternative 4-position substituents on triazoles **2n-q** were similarly less effective as the phenyl group in **2l**, but maintained activity particularly against ROCK2 kinase indicating the importance of the triazole pharmacophore. The efficacy of alcohol **2b**, thiocyanate **2d**, and triazoles (e.g., **2l** and **2n-q**) indicates that a hydrogen-bond acceptor may be important for activity.

## Conclusion

This study has provided useful tools for elucidating the structure and conformation-activity relationships responsible for peptide biology. Effective solid-phase methods have been developed for the synthesis of (3*R*,4*S*)-β-substituted-α-amino-γ-lactam (Agl) peptides employing *N*-Fmoc protected Agl-dipeptide building blocks. Studying the IL-1R allosteric modulator peptide **1**, the D-threonine residue was replaced by 15 different (3*R*,4*S*)-β-substituted-Agl^3^ analogs in peptides **2d-q**. Employing [(3*R*, 4*S*)-4-(N_3_)Agl^3^]-**1** (**2c**) as an intermediate, diverse amine and triazole substituents were synthesized using solid-phase methods by means of reduction and CuAAC chemistry.

Peptides **2d-q** were studied by CD spectroscopy and typically exhibited curve shapes indicative of β-turn conformation in contrast to peptide **1** which exhibited a random coil spectrum. Subsequently, β-substituted-Agl analogs **2d-q** were examined in a battery of *in vitro* assays for inhibitory activity on NF-κB signaling, on kinase phosphorylation (e.g., p38, JNK and ROCK2), and on expression of cytokines (e.g., IL-1 and COX2). Finally, six analogs (e.g., **2c**, **2d**, **2f**, **2l**, **2n**, and **2q**) were tested in models of PTB and OIR. Although peptides **1** and **2** all lacked inhibitory activity on the NF-κB signaling pathway, a spectrum of *in vitro* activities contingent on the β-substituent were exhibited by peptides **2**. Among peptides **2**, thiocyanate **2d**, hydroxylamine **2g**, and triazole analogs (e.g., **2l** and **2n-q**) exhibited inhibitory activity on the ROCK2 pathway. Moreover, 4-(phenyl)triazole **2l** and 4-hydroxymethyltriazole **2q** exhibited inhibitory activity on the JNK pathway. Capacity to reduce vaso-obliteration and microglial activation, hallmarks of retinopathy of prematurity, was exhibited in the OIR model by peptides (e.g., **2d**, **2l**, **2n**, and **2q**), which had demonstrated ROCK2 inhibitory activity. In the PTB model, peptides (e.g., **2l** and **2q**) delayed LPS-induced labor. The structure-activity relationships of β-substituted-Agl peptides **2d-q** offer insight into the requirements for pharmacological selectivity of IL-1R modulators. Moreover, with equal and better activity relative to peptide **1** in the *in vitro* and *in vivo* assays, 4-(phenyl)triazole **2l** epitomizes a valuable lead for developing a selective anti-inflammatory intervention strategy to delay preterm birth and improve neonatal outcomes without impeding immune vigilance.

## Data Availability Statement

The raw data supporting the conclusions of this article will be made available by the authors, without undue reservation.

## Ethics Statement

Timed pregnant CD-1 mice were used according to a protocol approved by the Animal Care Committee of Hôpital Sainte-Justine in accordance with the principles of the Guide for the Care and Use of Experimental Animals of the Canadian Council on Animal Care. All procedures and protocols involving the use of the rats were approved by the Animal Care Committee of the research center of Hôpital Maisonneuve-Rosemont and are in accordance with the Statement for the Use of Animals in Ophthalmic and Vision Research approved by the Association for Research in Vision and Ophthalmology, and guidelines established by the Canadian Council on Animal Care.

## Author Contributions

AG wrote the manuscript, synthesized and purified compounds, and conducted circular dichroism analyses. CC wrote the manuscript and conducted *in vivo* and *in vitro* experiments. CQ edited the manuscript and conducted *in vitro* experiments. XH edited the manuscript and conducted *in vivo* experiments. AB, FC, and IL performed *in vitro* experiments. SC and WL supervised the progress of the project, edited, and proofread the manuscript. All authors have read the final manuscript and agree to be accountable for the content of this work.

## Conflict of Interest

The authors declare that the research was conducted in the absence of any commercial or financial relationships that could be construed as a potential conflict of interest.

## Abbreviations

Boc: *tert*-butyloxycarbonyl
Fmoc: fluorenylmethyloxycarbonyl
DMF: dimethylformamide
FA: formic acid
TFA: trifluoroacetic acid
TLC: thin-layer chromatography
CuAAC: copper-catalyzed azide alkyne cycloaddition
IL-1R: interleukin-1 receptor
Agl: α-amino-γ-lactam
Hgl: β-hydroxy-α-amino-γ-lactam
PTB: preterm birth
ROP: retinopathy of prematurity
TCEP: tris(2-carboxyethyl)phosphine hydrochloride
DIEA: *N, N*-Diisopropylethylamine.
