# Supplementary material for: Interleukin-1 Receptor Modulation Using β-Substituted α-Amino-γ-Lactam Peptides From Solid-Phase Synthesis and Diversification
Source: Front Chem. 2020 Dec 21;8:610431. doi: 10.3389/fchem.2020.610431 (PMC7783595; doi:10.3389/fchem.2020.610431)
Supplement: Supplementary file 1 [file Data_Sheet_1.PDF]

Interleukin-1 Receptor Modulation using β-Substituted α-Amino-γ-Lactam Peptides from Solid-Phase Synthesis and Diversification

Azade Geranurimi[1#], Colin W.H. Cheng[2,3,4], Christiane Quiniou[3], France Cote[2], Xin Hou[3], Isabelle Lahaie[3], Amarilys Boudreault[3], Sylvain Chemtob[2,3,4,5], William D. Lubell[1]

[1]Département de Chimie, Université de Montréal, Montréal, QC, Canada
[2]Department of Pharmacology & Therapeutics, McGill University, Montréal, QC, Canada
[3]Hôpital Sainte-Justine Research Centre, Montréal, QC, Canada
[4]Hôpital Maisonneuve-Rosemont Research Centre, Montréal, QC, Canada
[5]Departments of Pediatrics, Pharmacology and Physiology, and Ophthalmology, Université de Montréal, Montréal, QC, Canada
# These authors contributed equally.

*** Correspondence:** Prof. William D. Lubell, william.lubell@umontreal.ca

Table of Contents:

LCMS chromatograms of peptides **2** p.2 – p.16

HRMS spectra of peptides **2** and **5f** p.17 – p.30

NMR spectra of peptide **5f** p.31– p.32

Supplementary Figure 1. Representative Western Blots of the JNK, ROCK2 and p38 proteins. p.33

[(3*R*, 4*S*)-β-N_3-Agl[3]]-101.10 (2c)

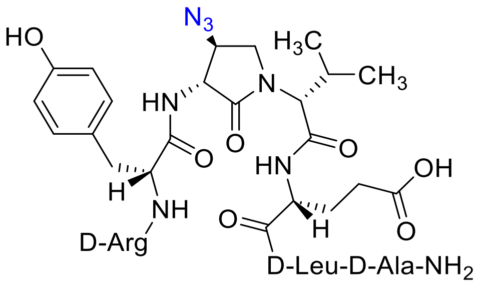

LCMS chromatogram [10-90% MeOH (0.1% FA)/water (0.1% FA), 14 min]; RT = 8.3 on a CE-C18, 3 x 50 mm, 2.7 um with a flow rate of 0.4 mL/min.

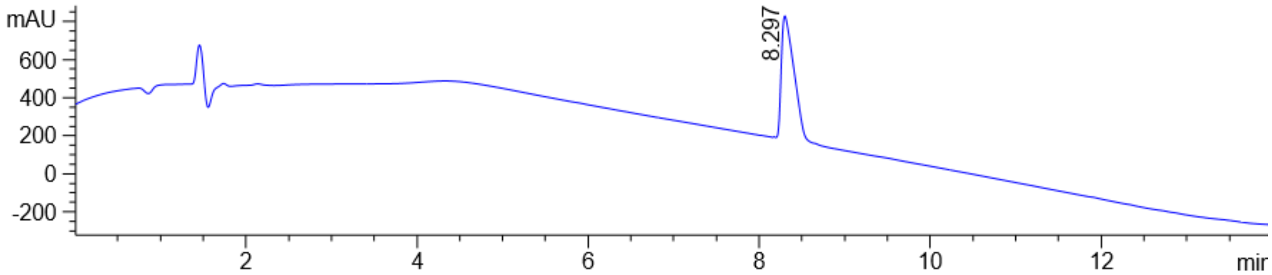

LCMS chromatogram [10-90% MeCN (0.1% FA)/water (0.1% FA), 14 min]; RT = 5.9 on a CE-C18, 3 x 50 mm, 2.7 um with a flow rate of 0.4 mL/min.

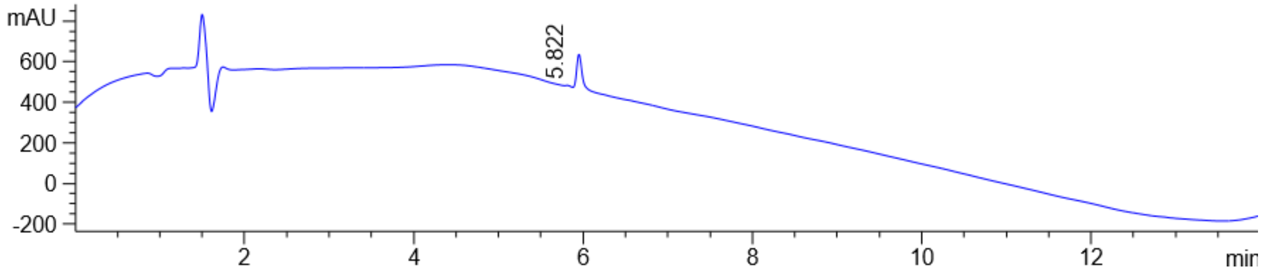

[(3*S*, 4*S*)-β-SCN-Agl[3]]-101.10 (2d)

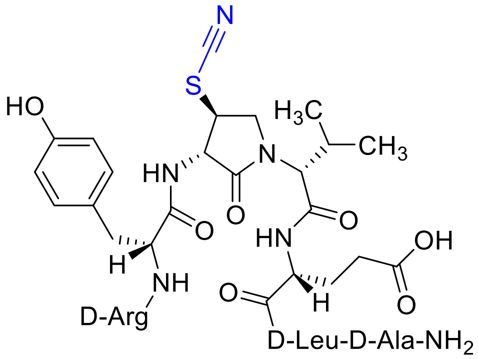

LCMS chromatogram [10-90% MeOH (0.1% FA)/water (0.1% FA), 14 min]; RT = 8.3 on a Sunfire C18 analytical column (100Å, 3.5 µm, 4.6 mm X 100 mm).

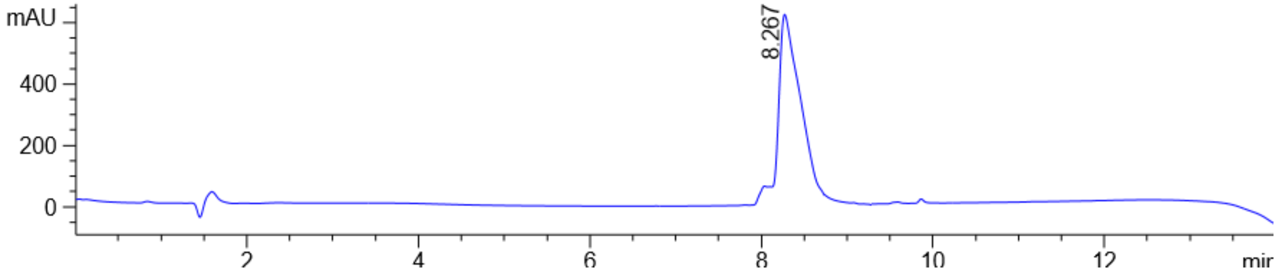

LCMS chromatogram [10-90% MeCN (0.1% FA)/water (0.1% FA), 14 min]; RT = 5.7 on a CE-C18, 3 x 50 mm, 2.7 um with a flow rate of 0.4 mL/min.

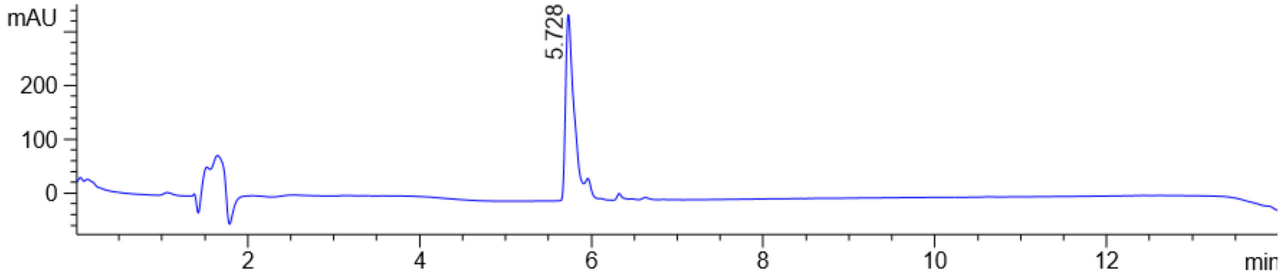

[(3*S*, 4*S*)-β-SMe-Agl[3]]-101.10 (2e)

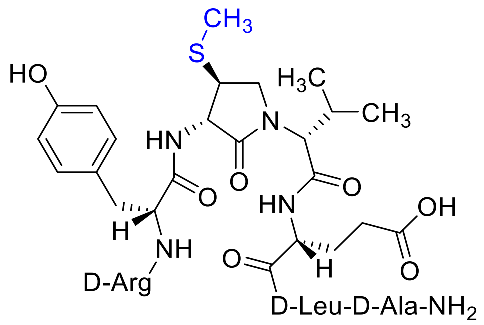

LCMS chromatogram [10-90% MeOH (0.1% FA)/water (0.1% FA), 14 min]; RT = 8.7 on a CE-C18, 3 x 50 mm, 2.7 um with a flow rate of 0.4 mL/min.

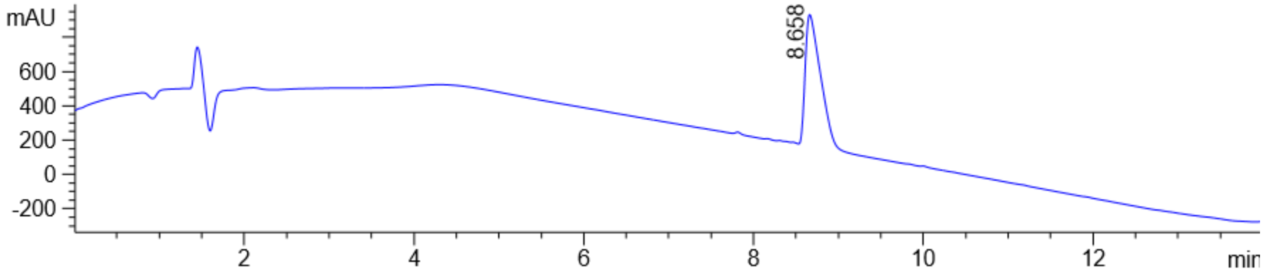

LCMS chromatogram [10-90% MeCN (0.1% FA)/water (0.1% FA), 14 min]; RT = 5.9 on a CE-C18, 3 x 50 mm, 2.7 um with a flow rate of 0.4 mL/min.

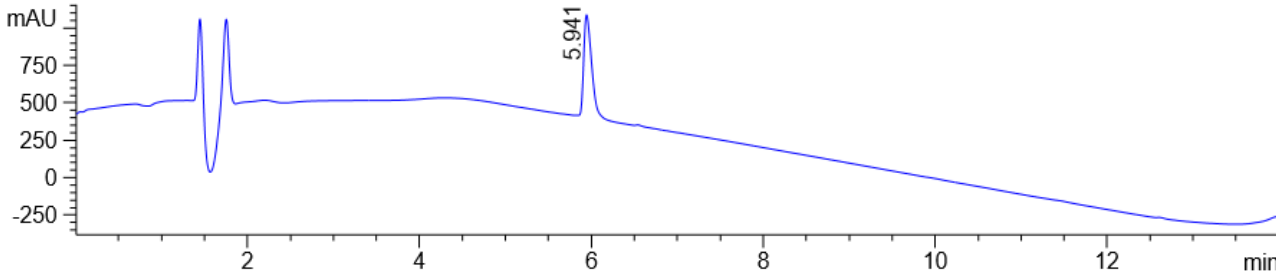

[(3*R*, 4*S*)-β-ONPhth-Agl[3]]-101.10 (2f)

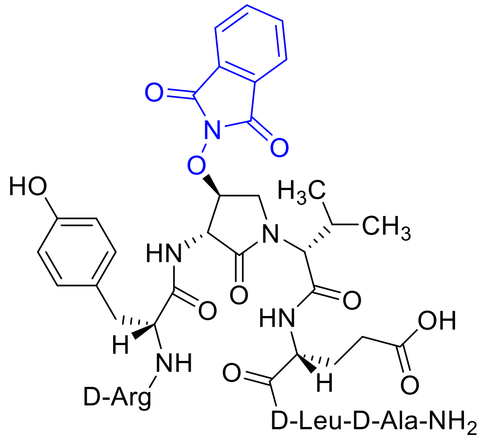

LCMS chromatogram [30-60-90% MeOH (0.1% FA)/water (0.1% FA), 15 min]; RT = 8.8 on a CSH-C18, 4.6 X100 mm, 5 um, with a flow rate of 0.8 mL/min.

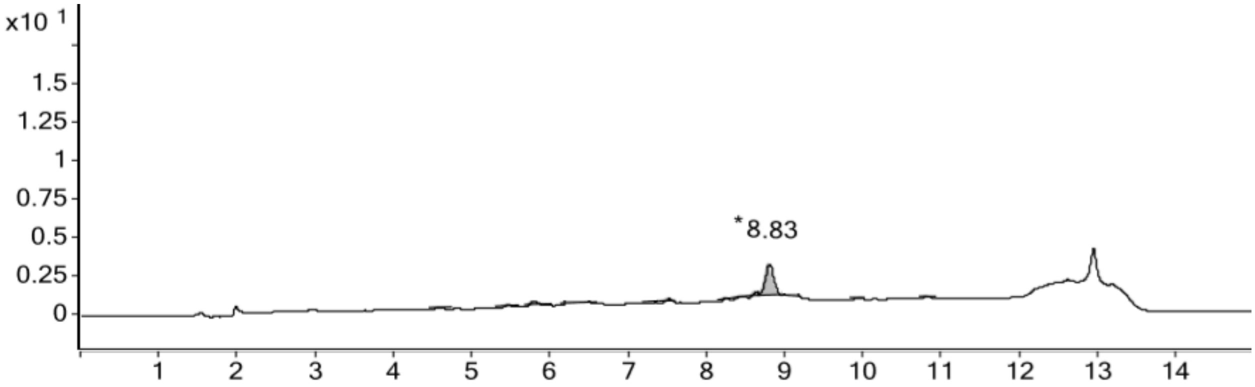

LCMS chromatogram [5-60-90% ACN (0.1% FA)/water (0.1% FA), 15 min]; RT = 8.8 on a CSH-C18, 4.6 X100 mm, 5 um, with a flow rate of 0.8 mL/min.

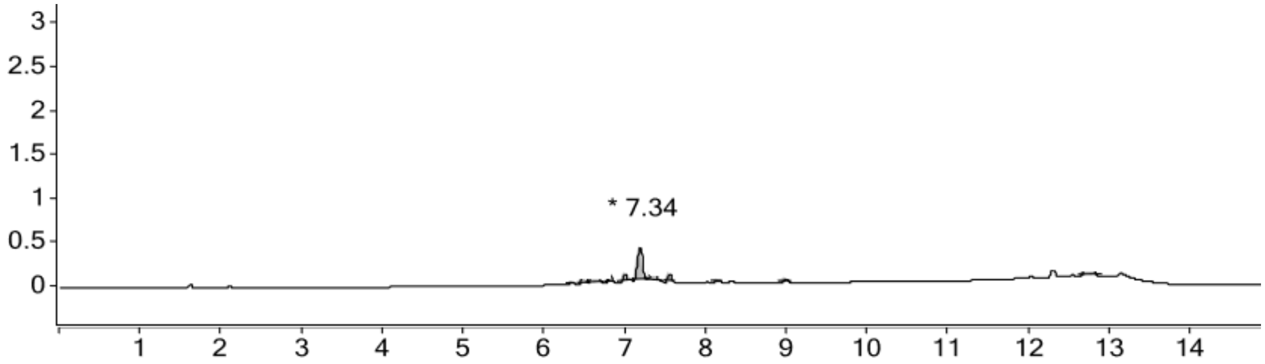

[(3*R*, 4*S*)-β-ONH$_2$-Agl^3]-101.10 (2g)

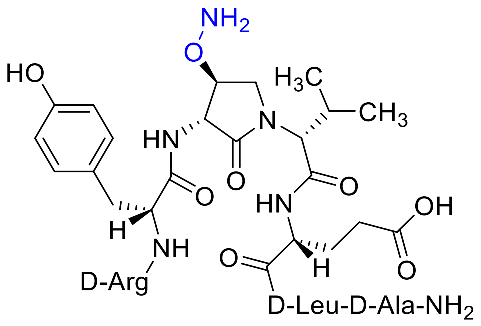

LCMS chromatogram [10-90% MeOH (0.1% FA)/water (0.1% FA), 14 min]; RT = 6.6 on a CE-C18, 3 x 50 mm, 2.7 um with a flow rate of 0.4 mL/min.

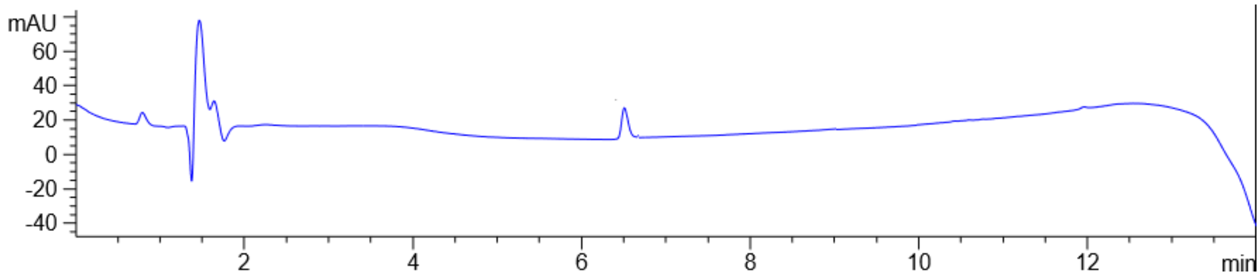

LCMS chromatogram [10-90% MeCN (0.1% FA)/water (0.1% FA), 14 min]; RT = 5.2 on a CE-C18, 3 x 50 mm, 2.7 um with a flow rate of 0.4 mL/min.

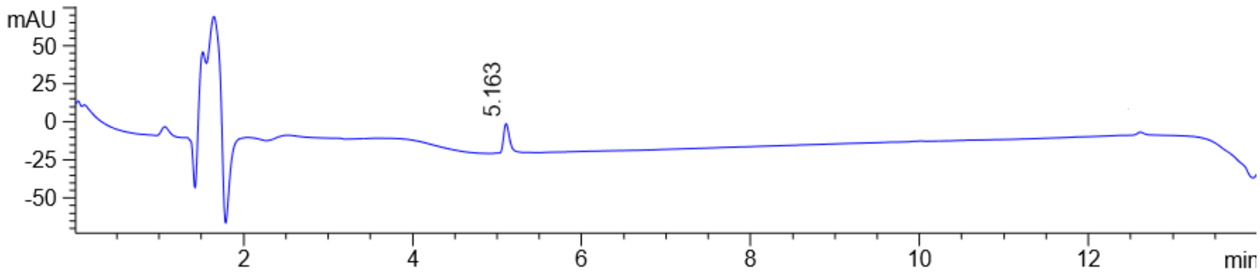

[(3*R*, 4*S*)-β-NH_2-Agl[3]]-101.10 (2h)

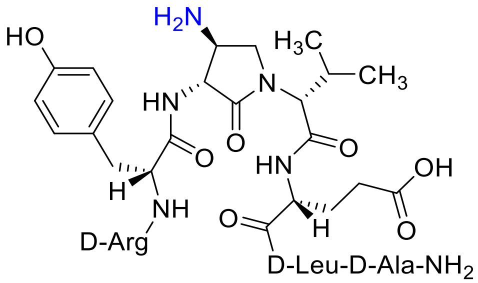

LCMS chromatogram [10-90% MeOH (0.1% FA)/water (0.1% FA), 14 min]; RT = 5.8 on a CE-C18, 3 x 50 mm, 2.7 um with a flow rate of 0.4 mL/min.

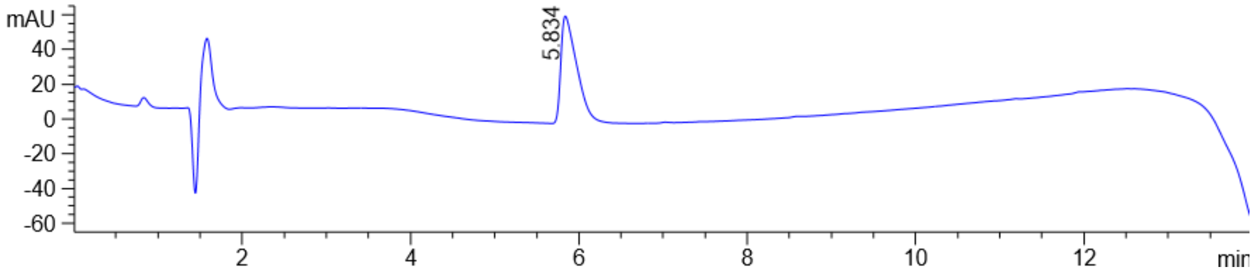

LCMS chromatogram [50-90% MeCN (0.1% FA)/water (0.1% FA), 14 min]; RT = 1.0 on a CE-C18, 3 x 50 mm, 2.7 um with a flow rate of 0.4 mL/min.

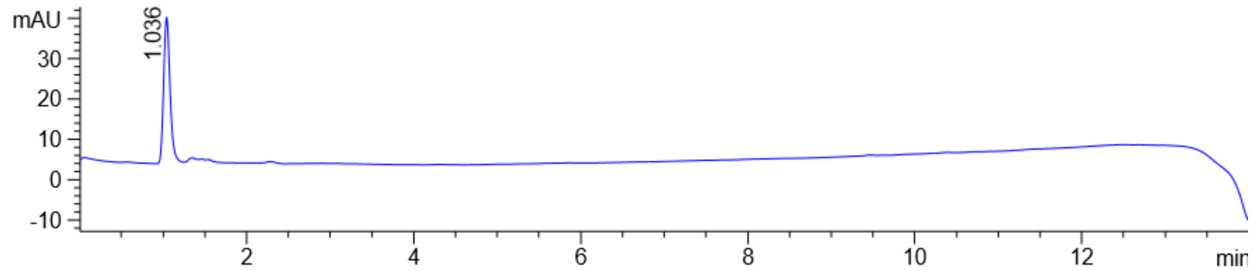

[(3*R*, 4*S*)-β-NH(C=O)Me-Agl[3]]-101.10 (2i)

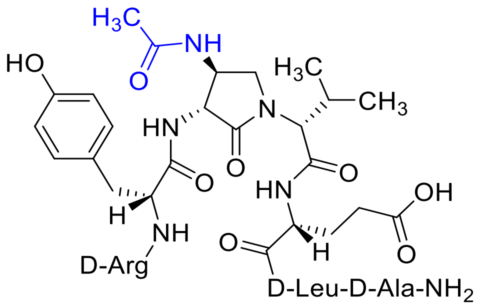

LCMS chromatogram [10-90% MeOH (0.1% FA)/water (0.1% FA), 14 min]; RT = 7.5 on a CE-C18, 3 x 50 mm, 2.7 um with a flow rate of 0.4 mL/min.

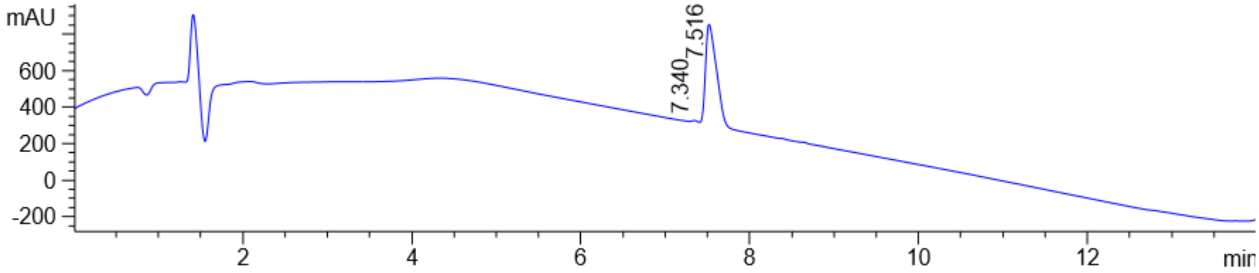

LCMS chromatogram [10-90% MeCN (0.1% FA)/water (0.1% FA), 14 min]; RT = 5.9 on a CE-C18, 3 x 50 mm, 2.7 um with a flow rate of 0.4 mL/min.

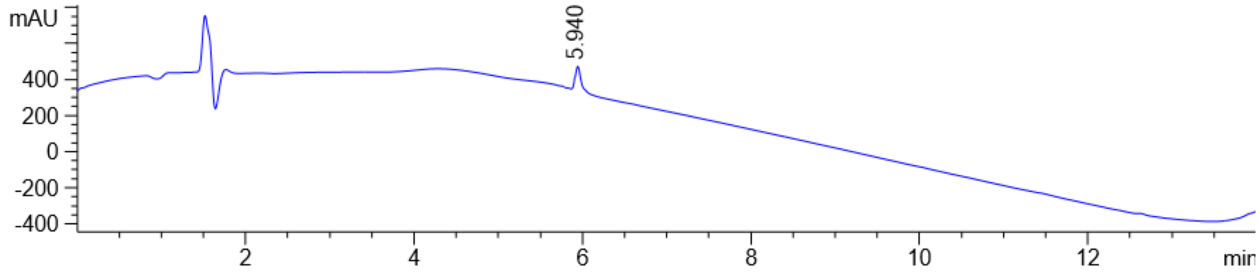

[(3*R*, 4*S*)-β-NH(C=O)NH_2-Agl[3]]-101.10 (2j)

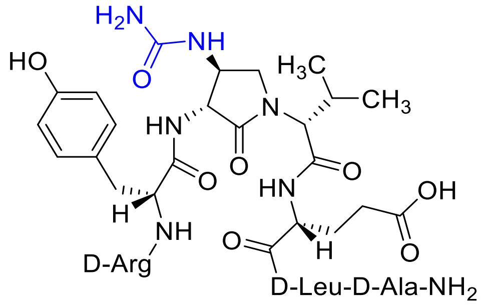

LCMS chromatogram [10-90% MeOH (0.1% FA)/water (0.1% FA), 14 min]; RT = 7.3 on a CE-C18, 3 x 50 mm, 2.7 um with a flow rate of 0.4 mL/min.

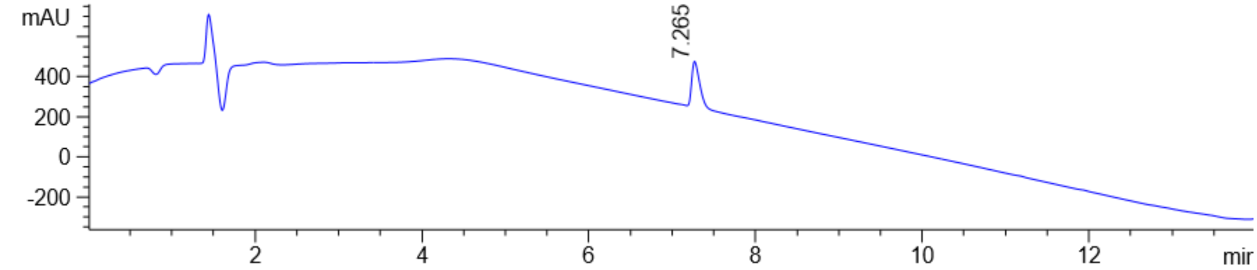

LCMS chromatogram [10-90% MeCN (0.1% FA)/water (0.1% FA), 14 min]; RT = 5.1 on a CE-C18, 3 x 50 mm, 2.7 um with a flow rate of 0.4 mL/min.

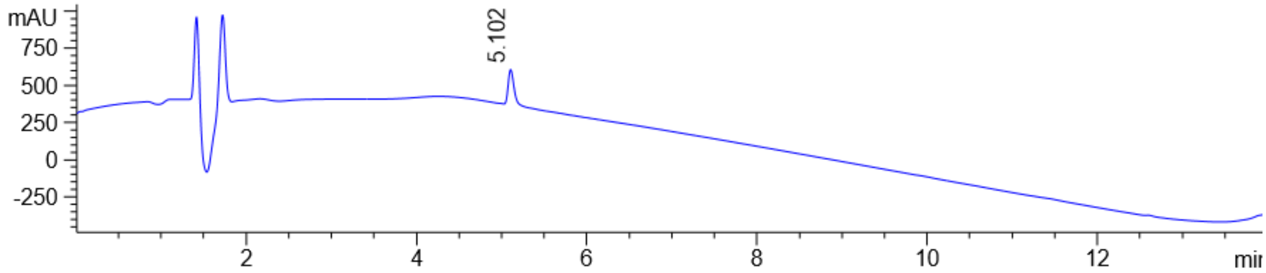

[(3*R*, 4*S*)-β-NH(C=N)NH_2-Agl[3]]-101.10 (2k)

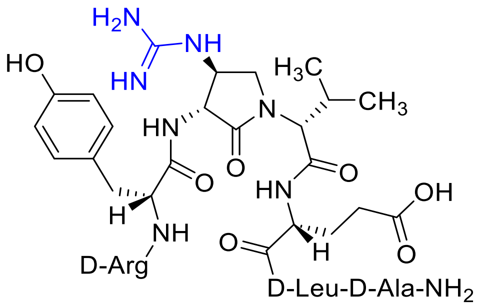

LCMS chromatogram [10-90% MeOH (0.1% FA)/water (0.1% FA), 14 min]; RT = 5.9 on a CE-C18, 3 x 50 mm, 2.7 um with a flow rate of 0.4 mL/min.

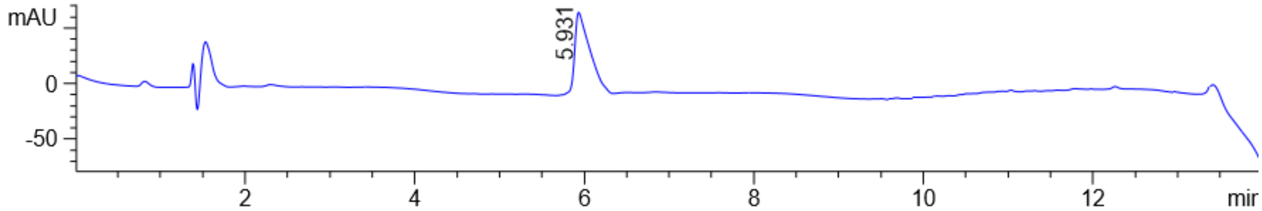

LCMS chromatogram [5-60-90% MeOH (0.1% FA)/water (0.1% FA), 15 min]; RT = 5.4 on a CSH C18, 4.6x100mm,5um, H_2O+0.1FA: MeOH

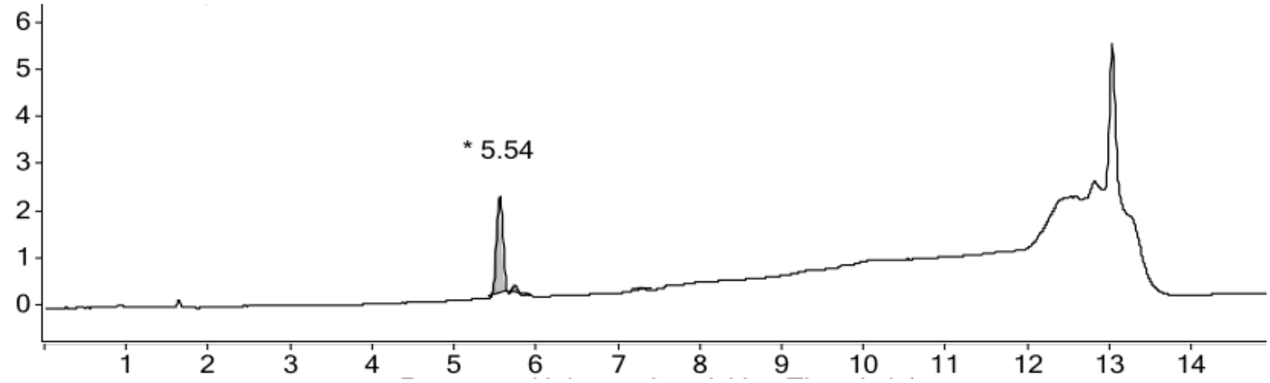

LCMS chromatogram [5-60-90% ACN (0.1% FA)/water (0.1% FA), 15 min]; RT = 4.6 on a CSH-C18, 4.6 X100 mm, 5 um, with a flow rate of 0.8 mL/min.

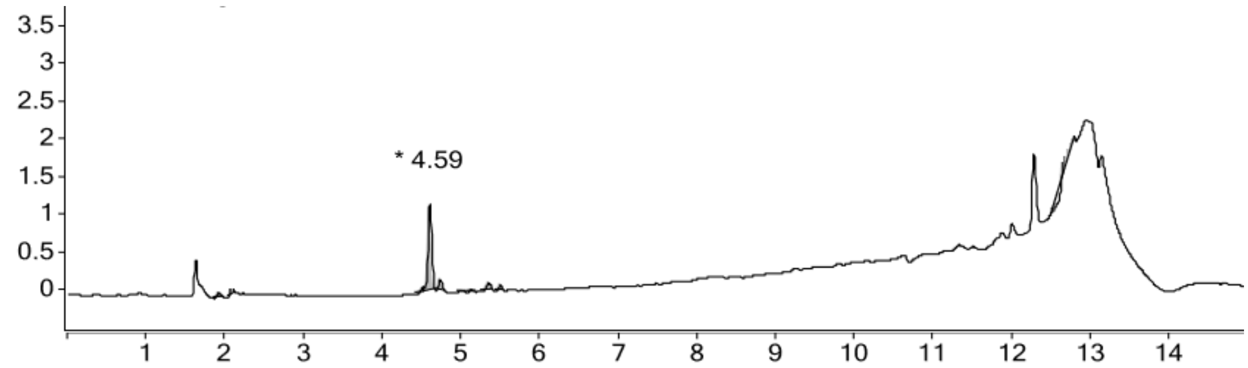

[(3*R*, 4*S*)-β-4-(Ph)triazolyl-Agl[3]]-101.10 (2l)

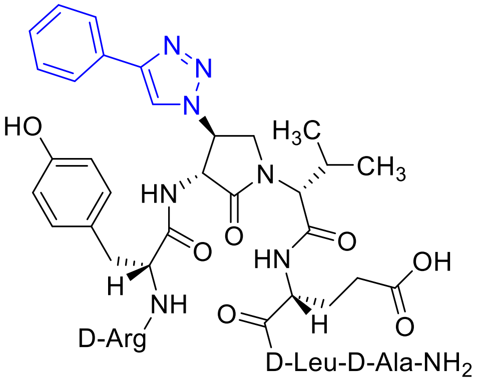

LCMS chromatogram [10-90% MeOH (0.1% FA)/water (0.1% FA), 14 min]; RT = 9.6 on a CE-C18, 3 x 50 mm, 2.7 um with a flow rate of 0.4 mL/min.

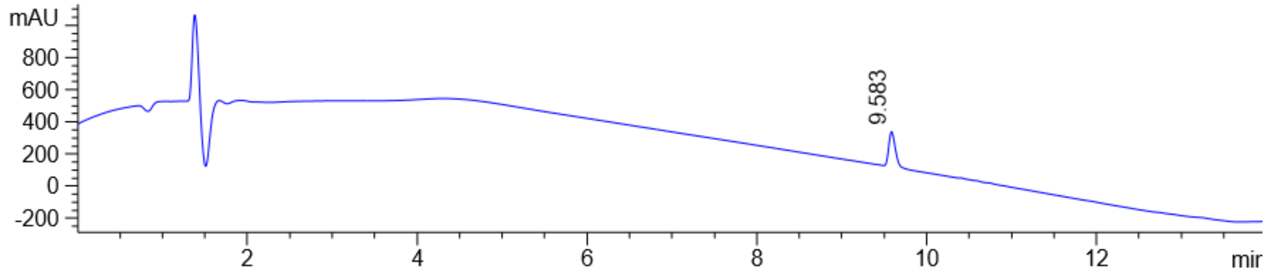

LCMS chromatogram [10-90% MeCN (0.1% FA)/water (0.1% FA), 14 min]; RT = 6.4 on a CE-C18, 3 x 50 mm, 2.7 um with a flow rate of 0.4 mL/min.

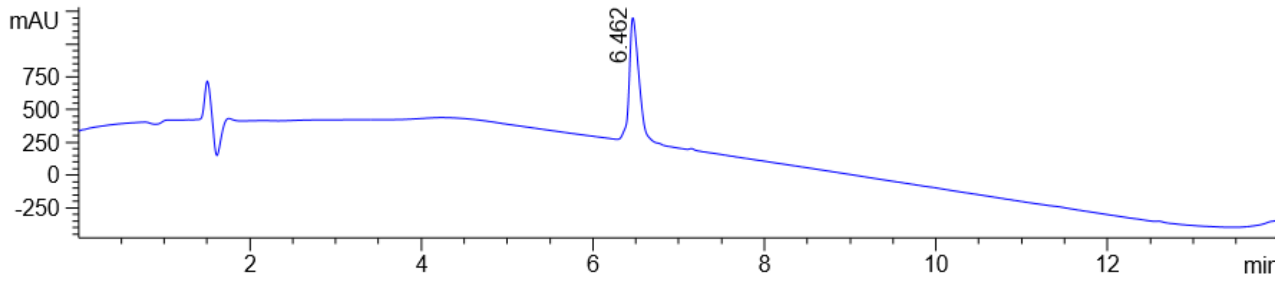

[(3*R*, 4*S*)-β-4-(*p*-MeC$_6$H$_4$)-triazolyl-Agl^3]-101.10 (2m)

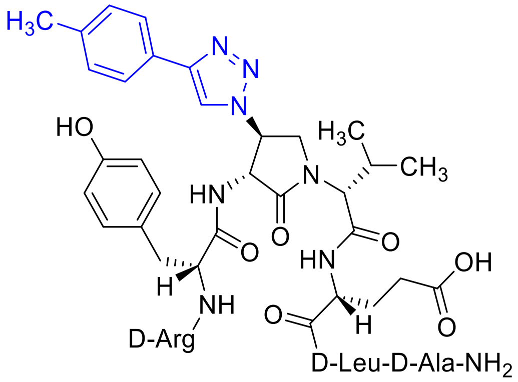

LCMS chromatogram [40-70-90% MeOH (0.1% FA)/water (0.1% FA), 15 min]; RT = 6.8 on a CSH-C18, 4.6 X100 mm, 5 um, with a flow rate of 0.8 mL/min.

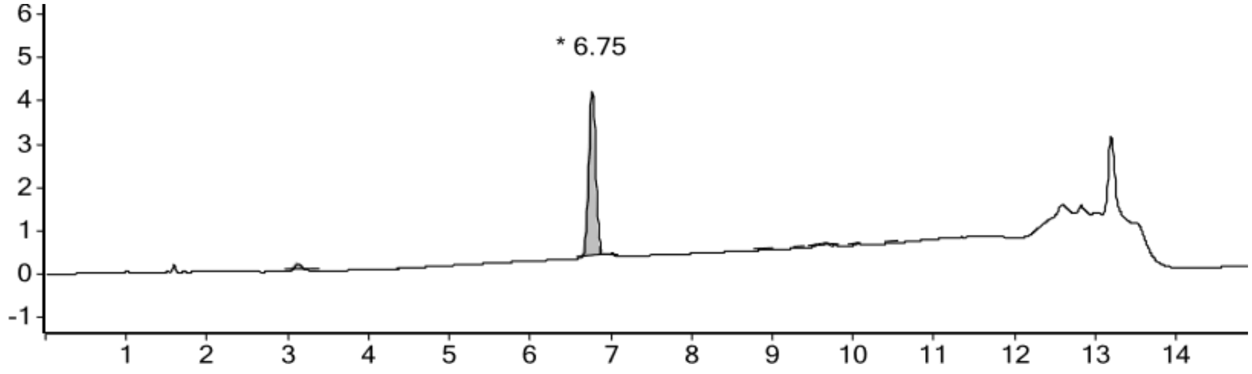

LCMS chromatogram [20-40-90% ACN (0.1% FA)/water (0.1% FA), 15 min]; RT = 6.5 on a CSH-C18, 4.6 X100 mm, 5 um, with a flow rate of 0.8 mL/min.

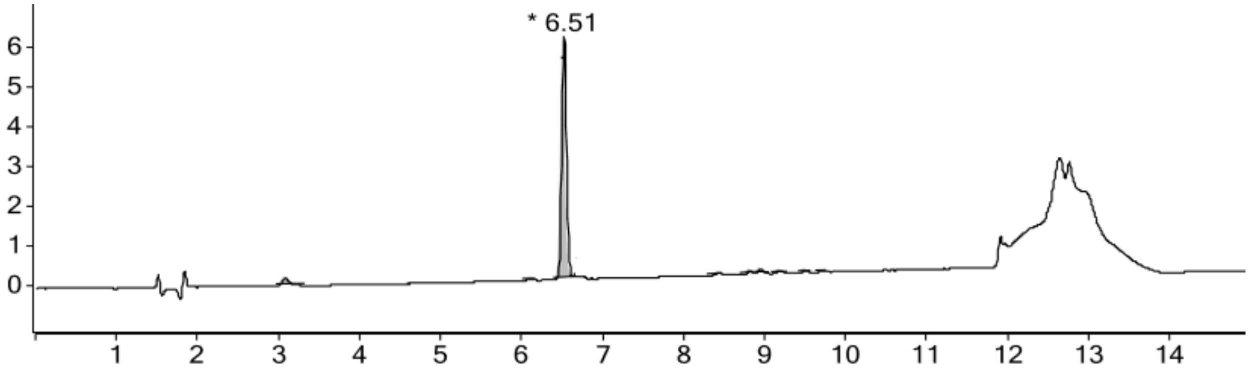

[(3*R*, 4*S*)-β-4-(*m*-$H_2NC_6H_4$)-triazolyl-Agl[3]]-101.10 (2n)

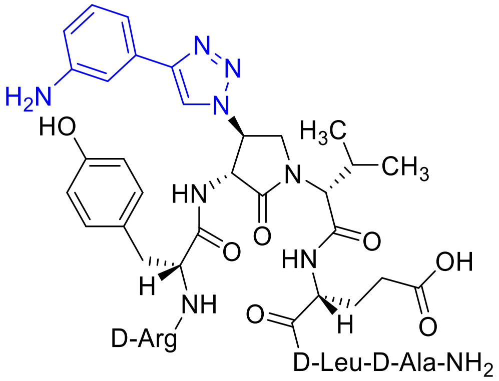

LCMS chromatogram [5-60-90% MeOH (0.1% FA)/water (0.1% FA), 15 min]; RT = 8.8 on a CSH-C18, 4.6 X100 mm, 5 um, with a flow rate of 0.8 mL/min.

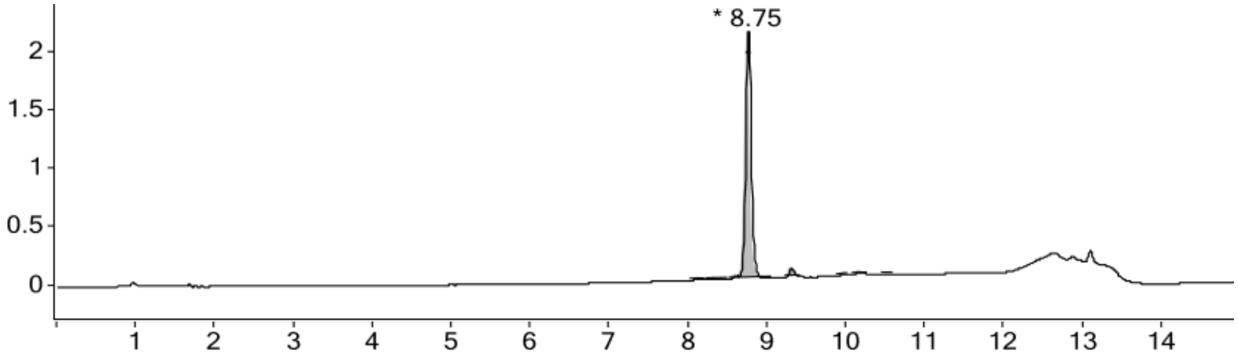

LCMS chromatogram [5-60-90% ACN (0.1% FA)/water (0.1% FA), 15 min]; RT = 6.1 on a CSH-C18, 4.6 X100 mm, 5 um, with a flow rate of 0.8 mL/min.

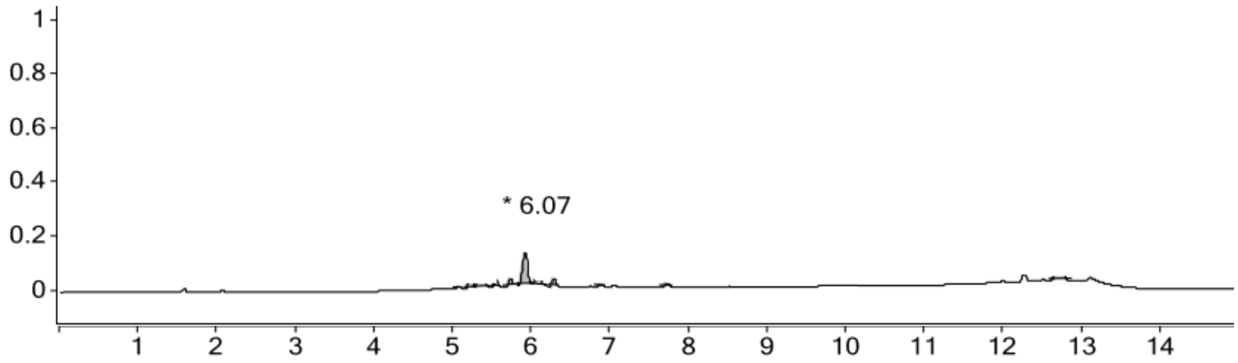

[(3*R*, 4*S*)-β-4-($H_2N(H_3C)_2C$)-triazolyl-Agl^3]-101.10 (2o)

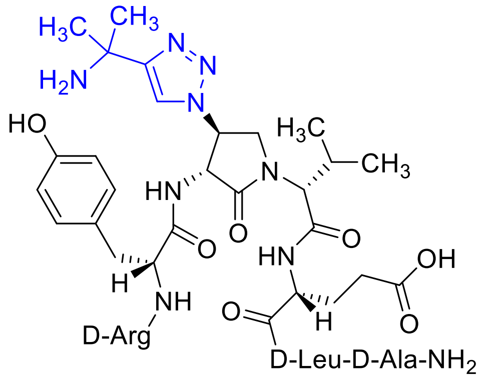

LCMS chromatogram [5-60-90% MeOH (0.1% FA)/water (0.1% FA), 15 min]; RT = 6.3 on a CSH-C18, 4.6 X100 mm, 5 um, with a flow rate of 0.8 mL/min.

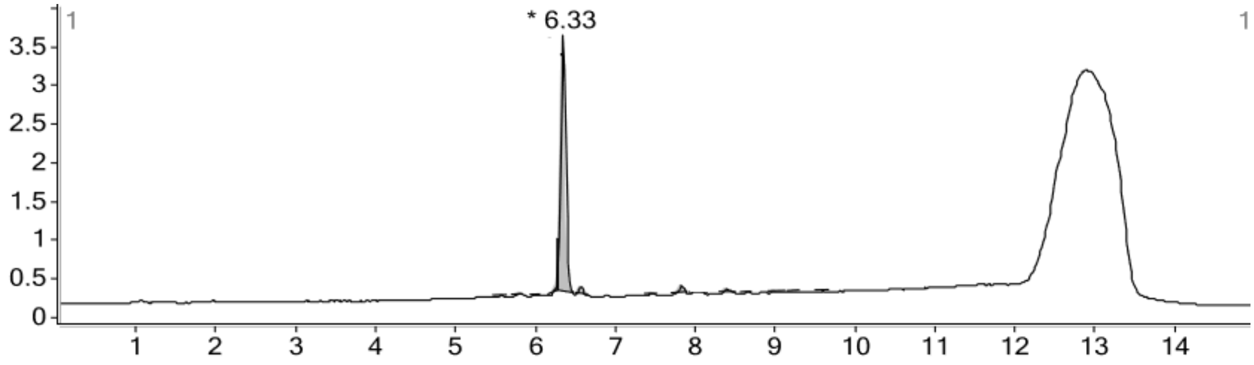

LCMS chromatogram [5-60-90% ACN (0.1% FA)/water (0.1% FA), 15 min]; RT = 4.9 on a CSH-C18, 4.6 X100 mm, 5 um, with a flow rate of 0.8 mL/min.

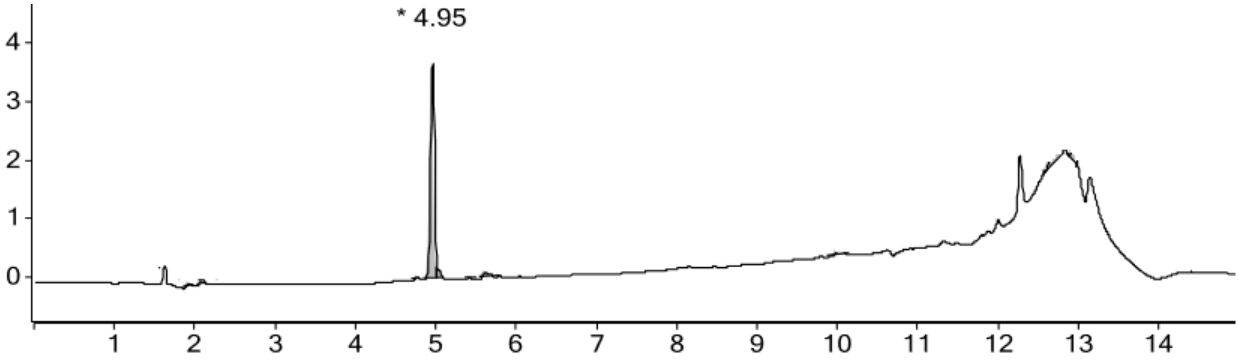

[(3*R*, 4*S*)-β-4-(H_2NH_2C)-triazolyl-Agl^3]-101.10 (2p)

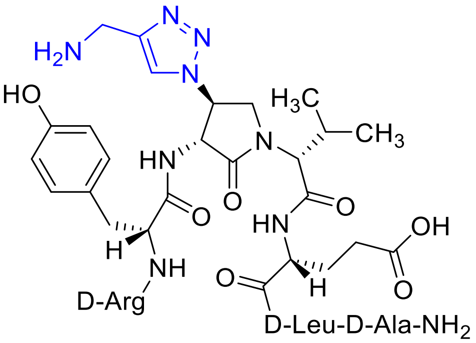

LCMS chromatogram [5-60-90% MeOH (0.1% FA)/water (0.1% FA), 15 min]; RT = 7.9 on a CSH-C18, 4.6 X100 mm, 5 um, with a flow rate of 0.8 mL/min.

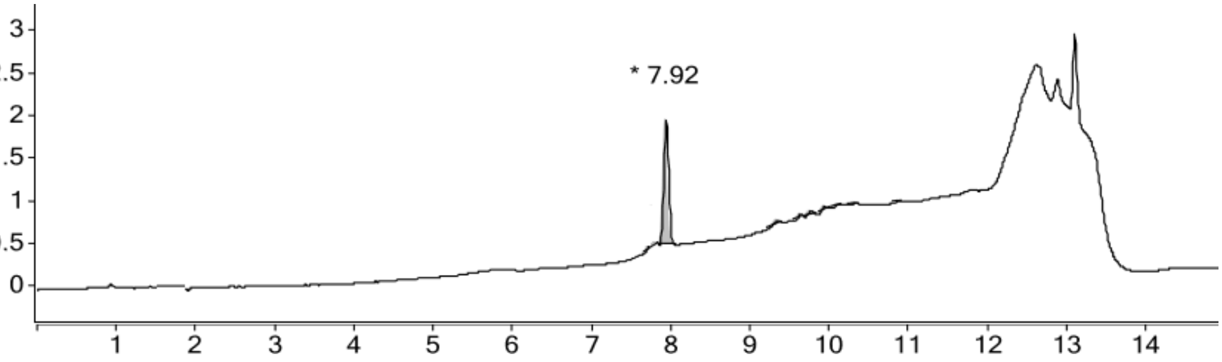

LCMS chromatogram [5-60-90% ACN (0.1% FA)/water (0.1% FA), 15 min]; RT = 6.1 on a

CSH-C18, 4.6 X100 mm, 5 um, with a flow rate of 0.8 mL/min.

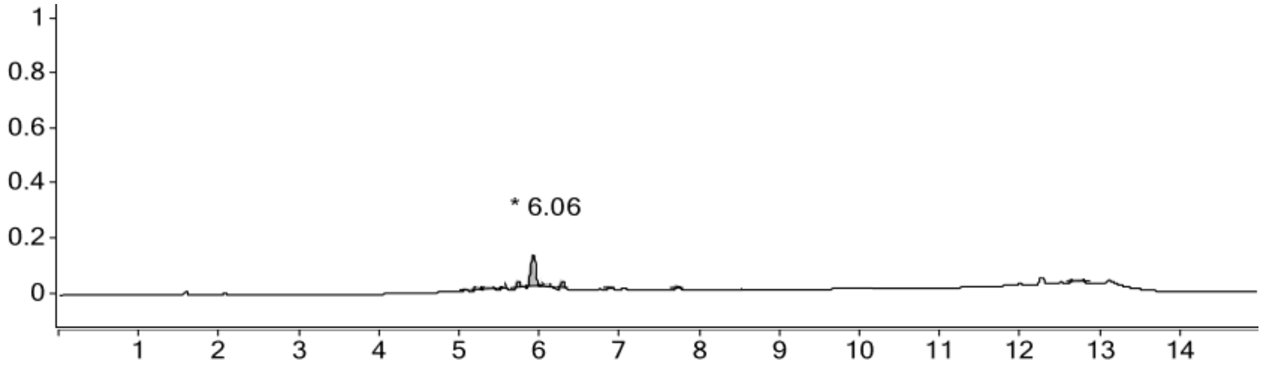

[(3*R*, 4*S*)-β-4-(HOH$_2$C)-triazolyl-Agl^{3}]-101.10 (2q)

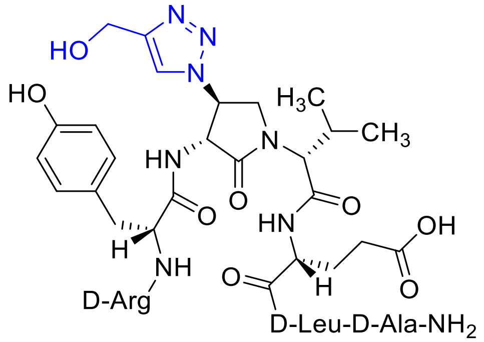

LCMS chromatogram [5-60-90% MeOH (0.1% FA)/water (0.1% FA), 15 min]; RT = 7.2 on a CSH-C18, 4.6 X100 mm, 5 um, with a flow rate of 0.8 mL/min.

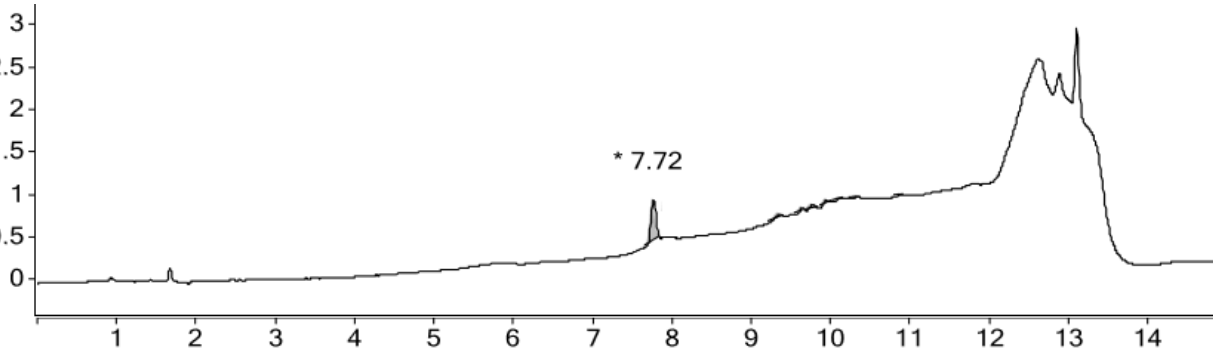

LCMS chromatogram [5-60-90% ACN (0.1% FA)/water (0.1% FA), 15 min]; RT = 5.7 on a CSH-C18, 4.6 X100 mm, 5 um, with a flow rate of 0.8 mL/min.

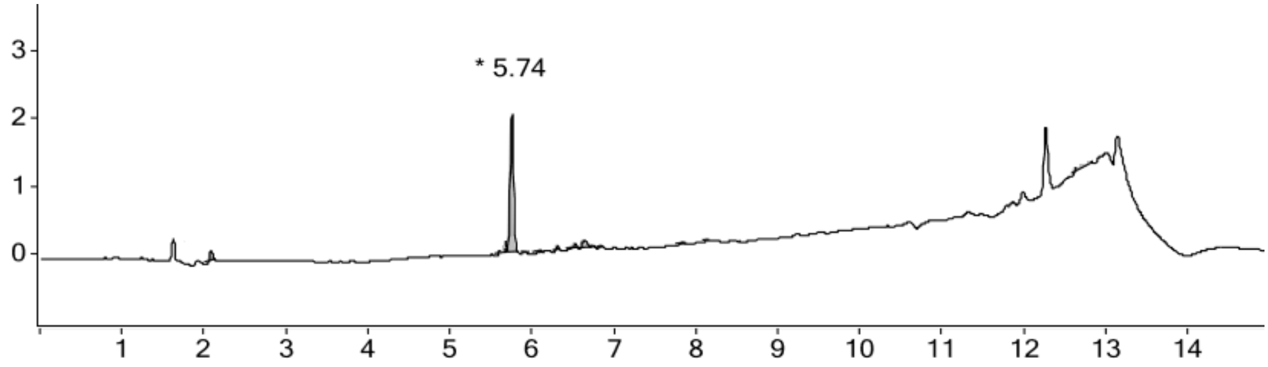

[(3*R*, 4*S*)-β-N_3-Agl[3]]-101.10 (2c)

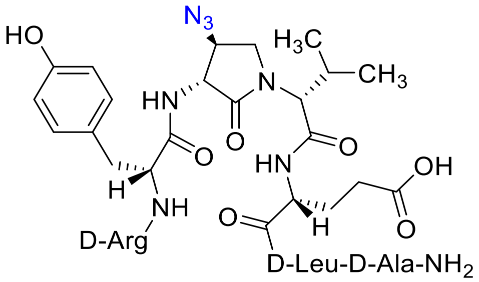

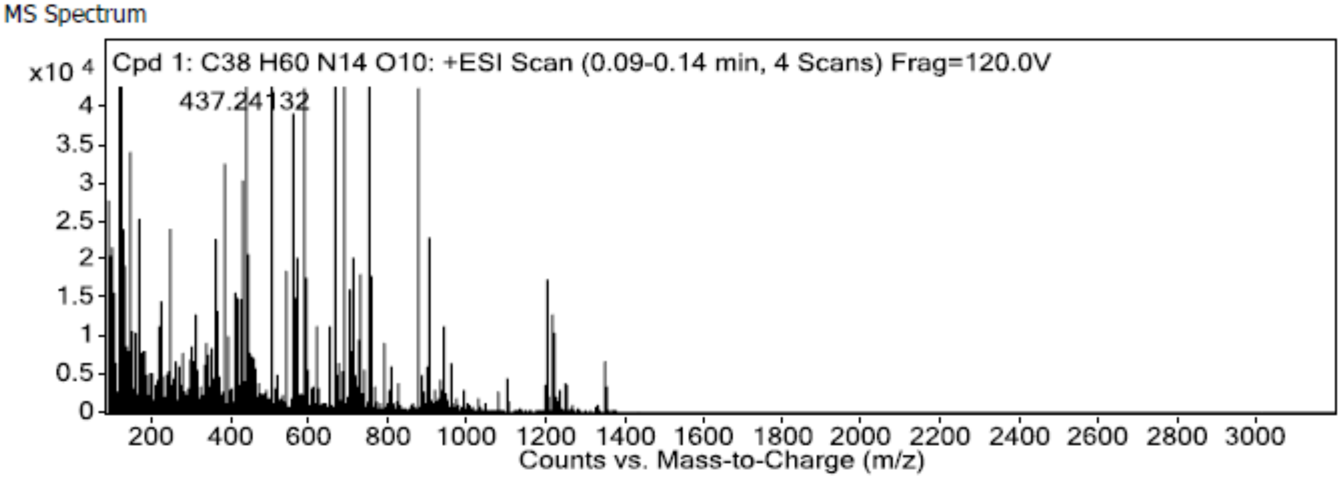

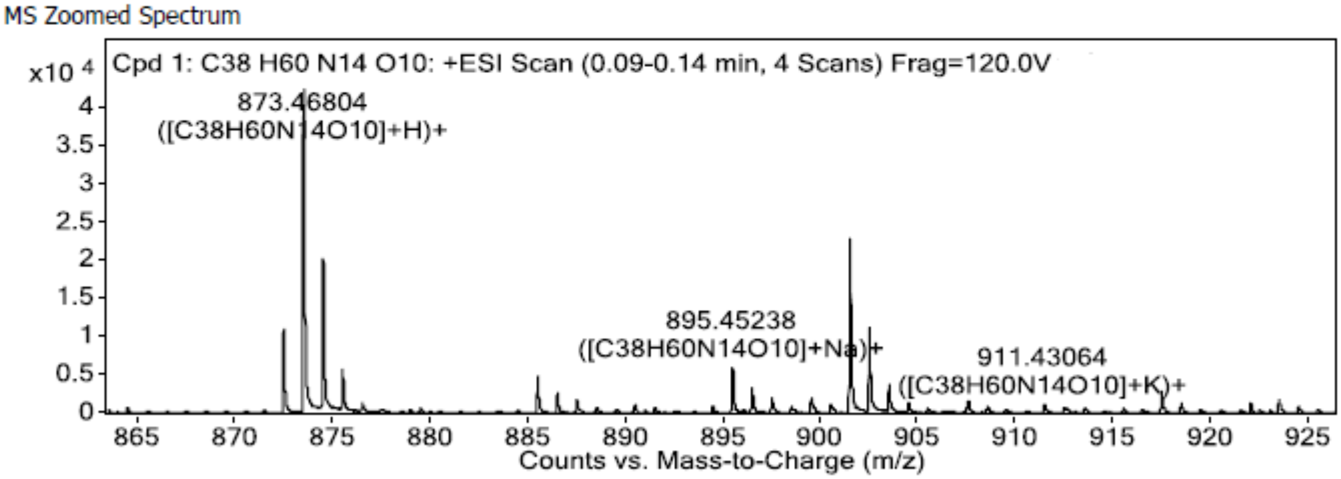

MS Spectrum Peak List

Ion	Formula	Abund	*Expe. m/z*	*Calc. m/z*	Diff(ppm)
(M+H)+	C38H60N14O10	42373.1	873.46804	873.46896	1.05
(M+Na)+	C38H60N14O10	6176.34	895.45238	895.45091	-1.65

[(3*S*, 4*S*)-β-SCN-Agl[3]]-101.10 (2d)

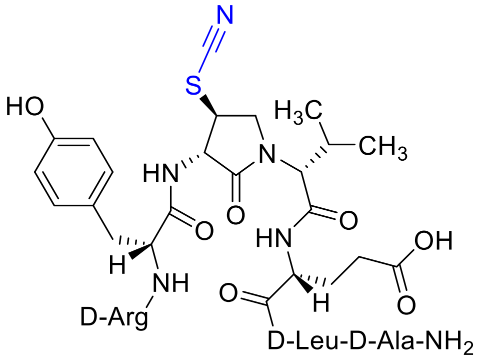

MS Spectrum

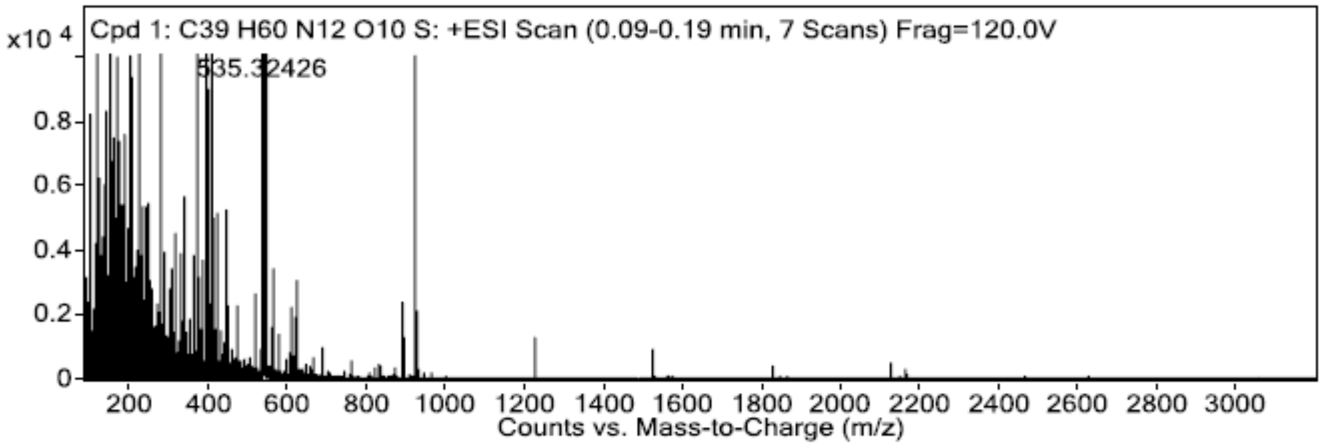

MS Zoomed Spectrum

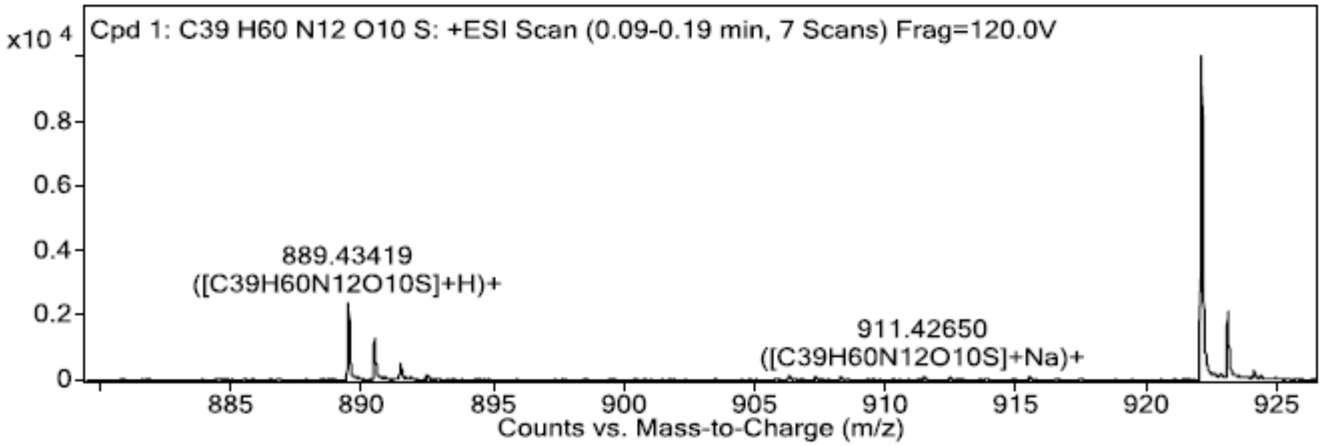

MS Spectrum Peak List

Ion	Formula	Abund	*Expe. m/z*	*Calc. m/z*	Diff(ppm)
(M+H)+	C39H60N12O10S	2457.46	889.43419	889.43488	0.78

[(3*S*, 4*S*)-β-SMe-Agl[3]]-101.10 (2e)

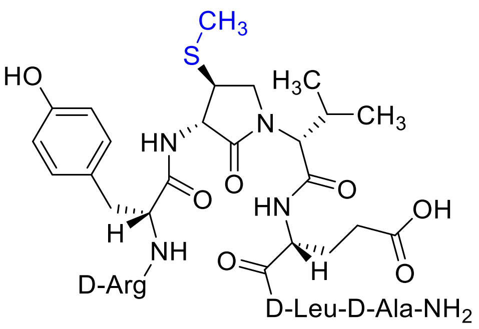

MS Spectrum

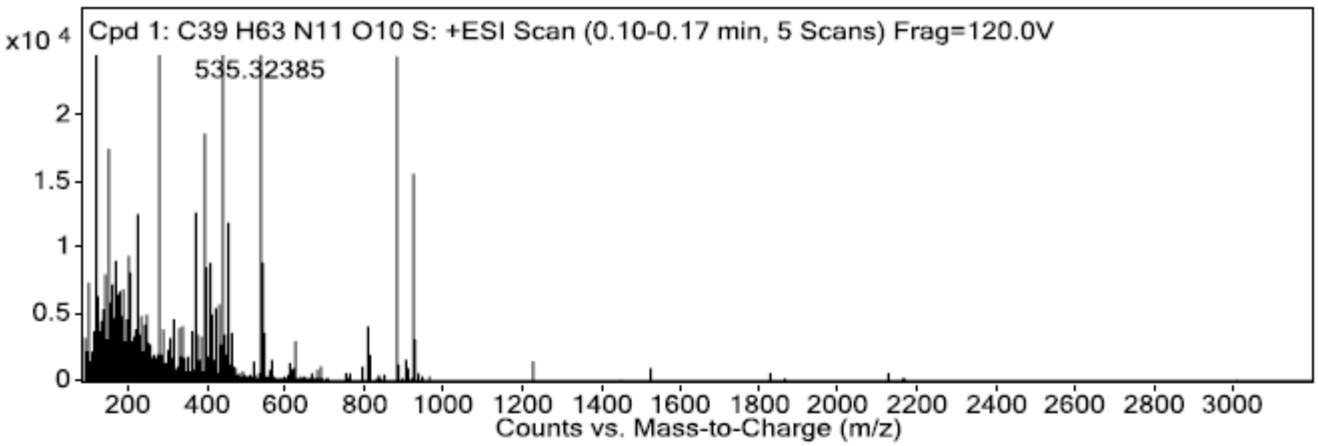

MS Zoomed Spectrum

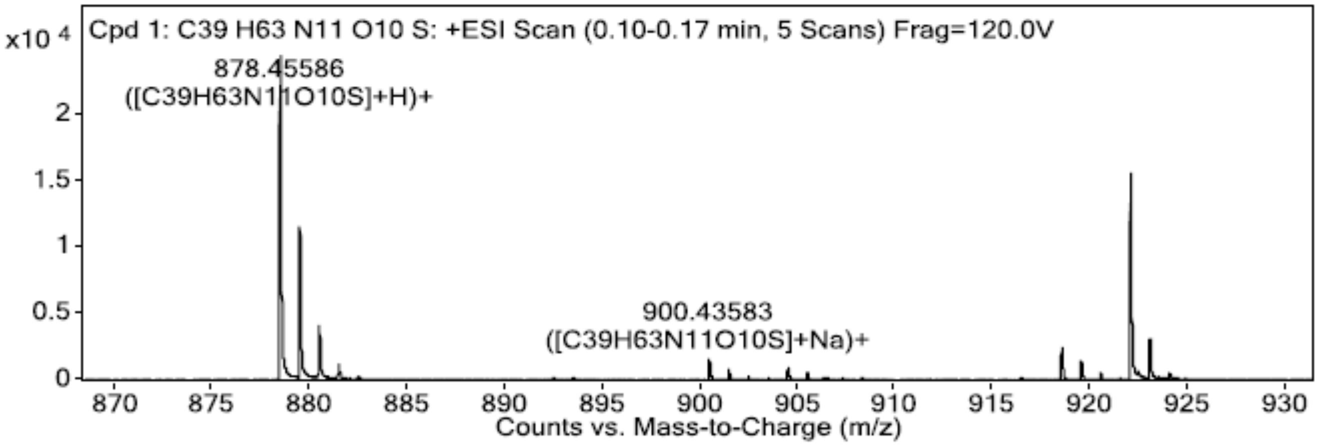

MS Spectrum Peak List

Ion	Formula	Abund	*Expe. m/z*	*Calc. m/z*	Diff(ppm)
(M+H)+	C39H63N11O10S	24464.72	878.45586	878.45528	-0.65
(M+Na)+	C39H63N11O10S	1690.57	900.43583	900.43723	1.55

[(3*R*, 4*S*)-β-ONPhth-Agl[3]]-101.10 (2f)

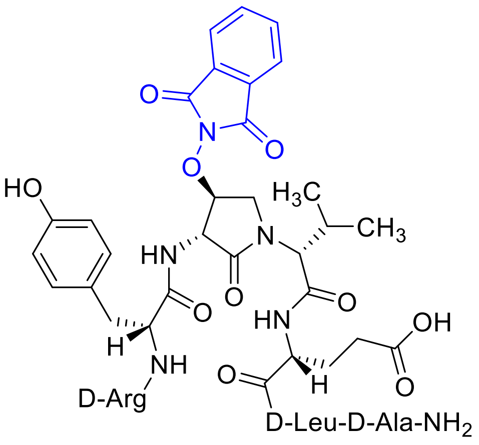

MS Spectrum

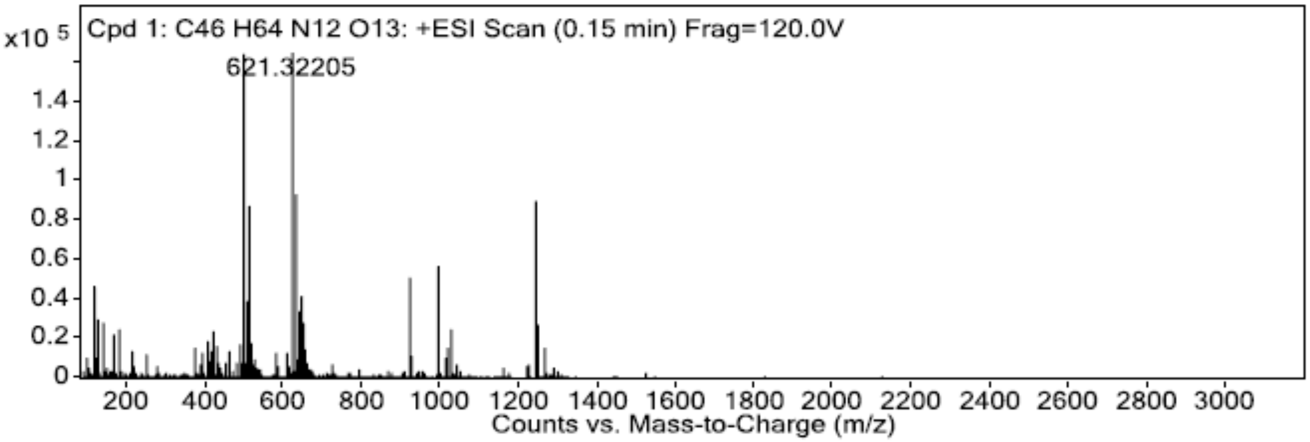

MS Zoomed Spectrum

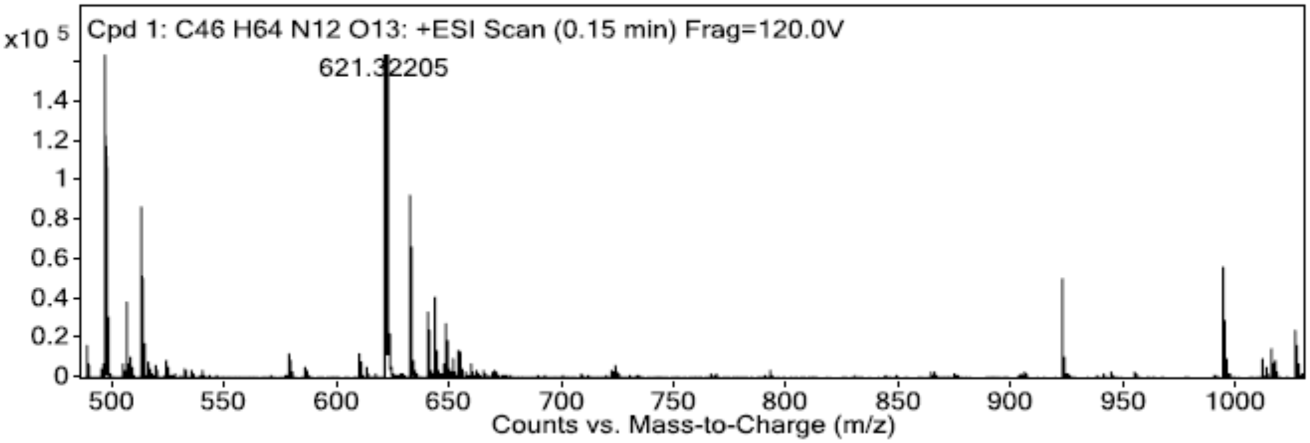

MS Spectrum Peak List

Ion	Formula	Abund	*Expe. m/z*	*Calc. m/z*	Diff(ppm)
(M+2H)+2	C46H64N12O13	163957.45	497.24508	497.24307	-4.05
(M+H)+	C46H64N12O13	56461.7	993.47861	993.47886	0.25
(M+Na)+	C46H64N12O13	15575.33	1015.45905	1015.4608	1.73

[(3*R*, 4*S*)-β-NH_2-Agl[3]]-101.10 (2h)

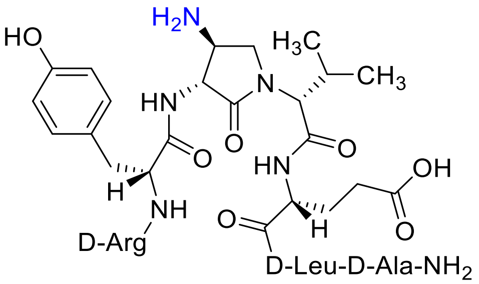

MS Spectrum

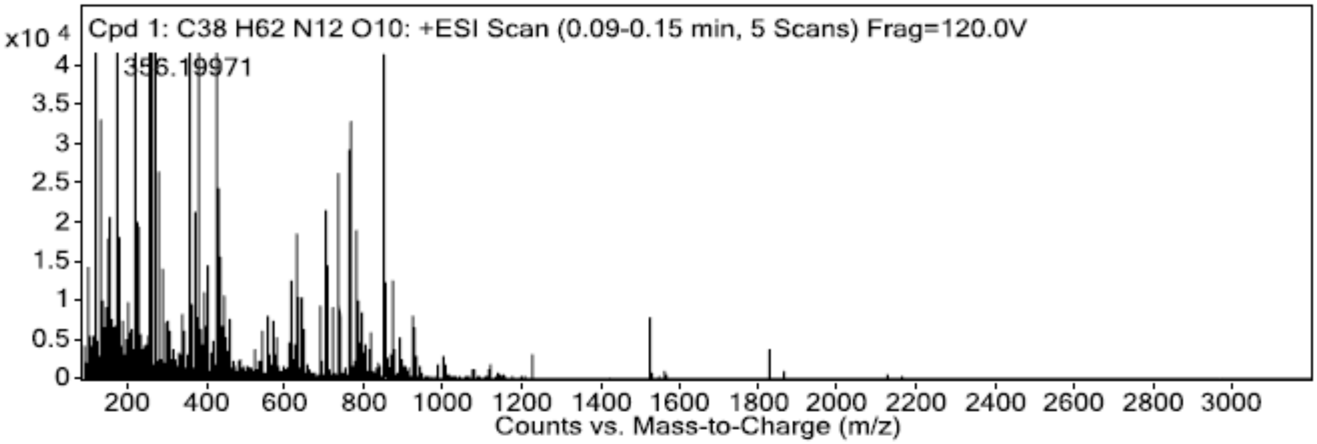

MS Zoomed Spectrum

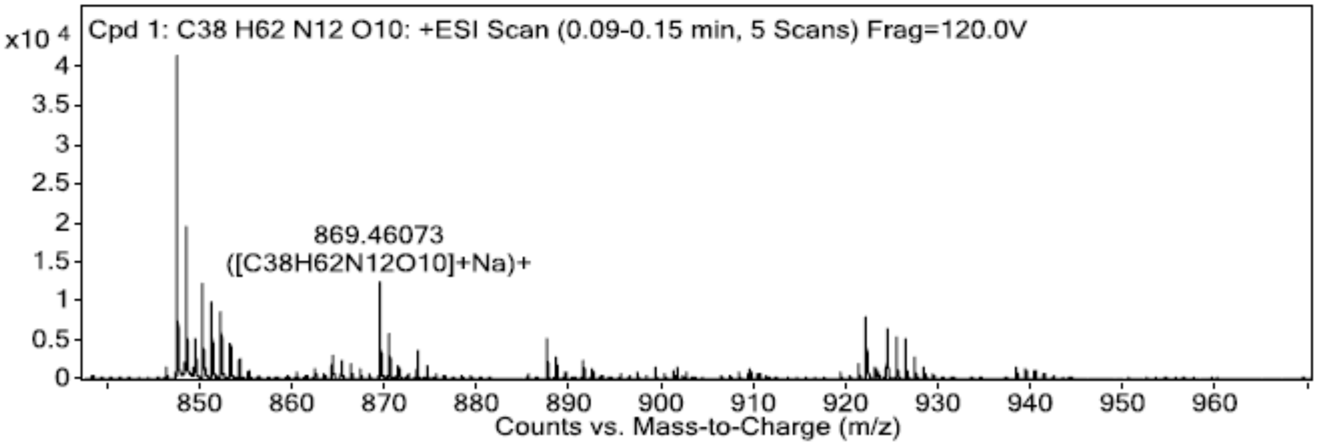

MS Spectrum Peak List

Ion	Formula	Abund	*Expe. m/z*	*Calc. m/z*	Diff(ppm)
(M+H)+	C38H62N12O10	41829.08	847.47796	847.47846	0.59
(M+Na)+	C38H62N12O10	12666.06	869.46073	869.46041	-0.38

[(3*R*, 4*S*)-β-NH(C=O)Me-Agl[3]]-101.10 (2i)

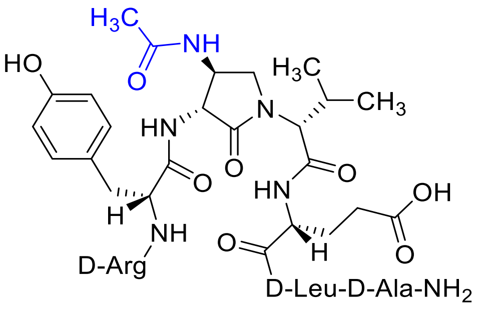

MS Spectrum

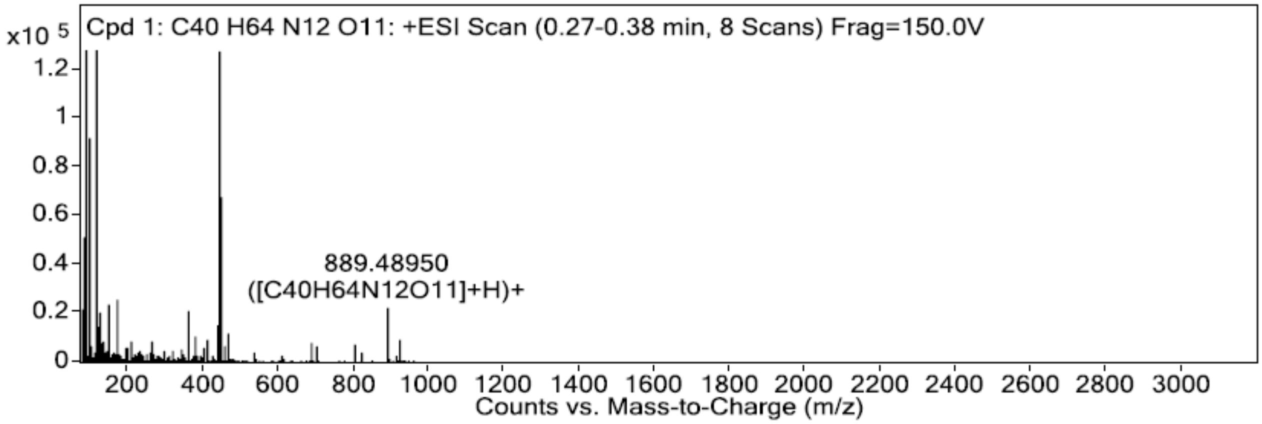

MS Zoomed Spectrum

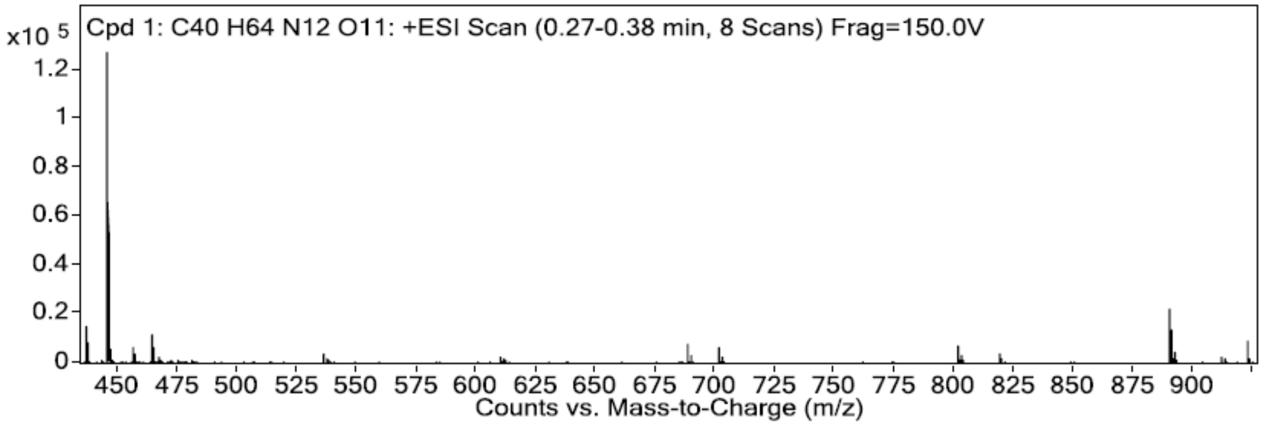

MS Spectrum Peak List

Ion	Formula	Abund	*Expe. m/z*	*Calc. m/z*	Diff(ppm)
(M+H)+	C40H64N12O11	22492.48	889.4895	889.48903	-0.53

[(3*R*, 4*S*)-β-NH(C=O)NH_2-Agl^3]-101.10 (2j)

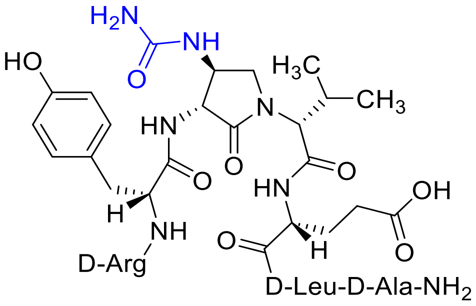

MS Spectrum

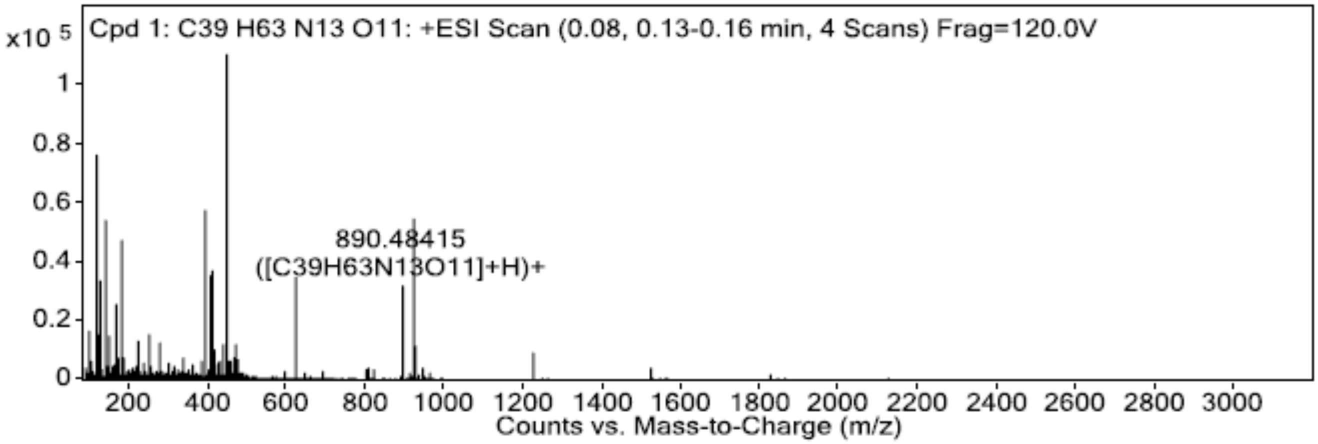

MS Zoomed Spectrum

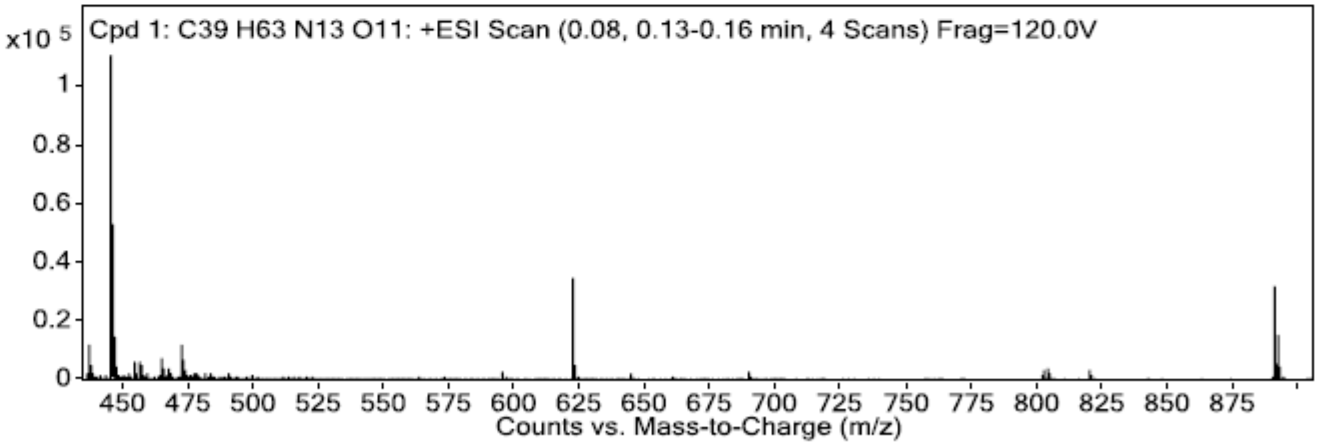

MS Spectrum Peak List

Ion	Formula	Abund	*Expe. m/z*	*Calc. m/z*	Diff(ppm)
(M+2H)+2	C39H63N13O11	110564.11	445.74771	445.74578	-4.34
(M+H)+	C39H63N13O11	32071.88	890.48415	890.48428	0.14

[(3*R*, 4*S*)-β-NH(C=N)NH$_2$-Agl^3]-101.10 (2k)

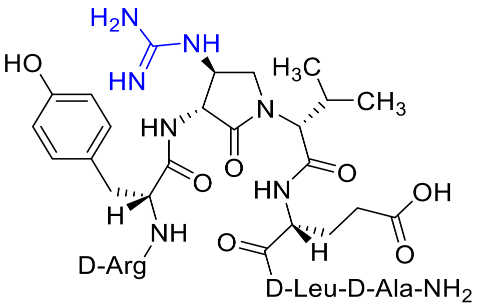

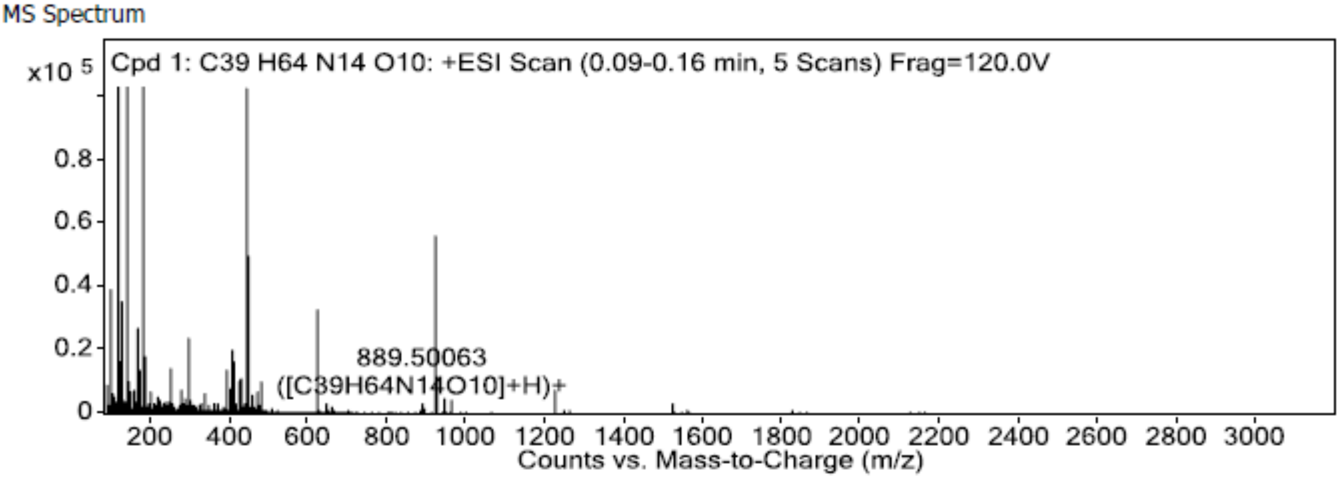

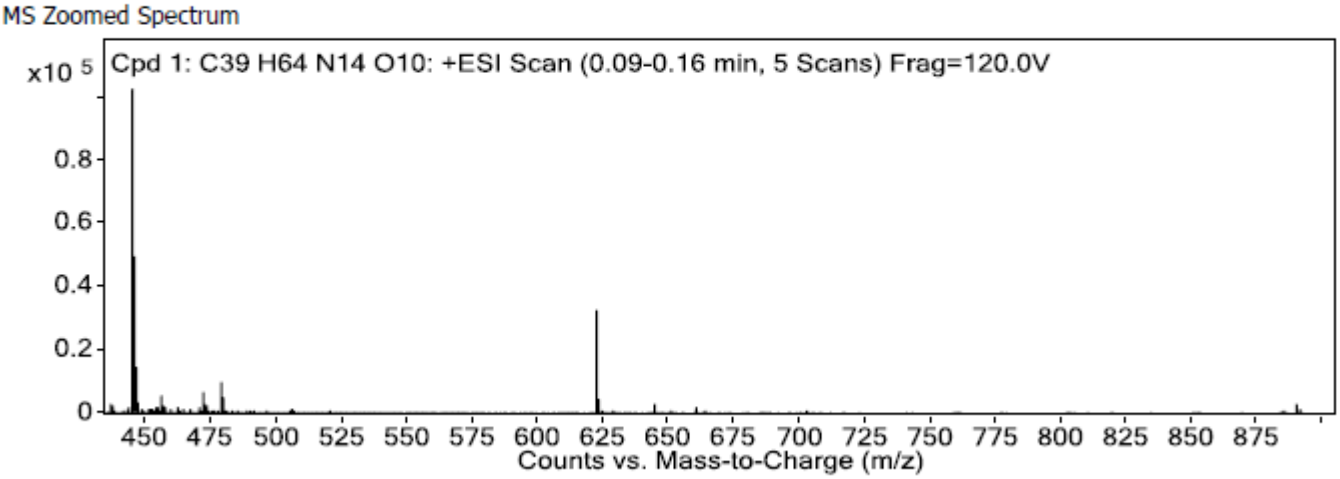

MS Spectrum Peak List

Ion	Formula	Abund	*Expe. m/z*	*Calc. m/z*	Diff(ppm)
(M+2H)+2	C39H64N14O10	102551.3	445.25535	445.25377	-3.55
(M+H)+	C39H64N14O10	3032.94	889.50063	889.50026	-0.42

[(3*R*, 4*S*)-β-4-(Ph)triazolyl-Agl[3]]-101.10 (2l)

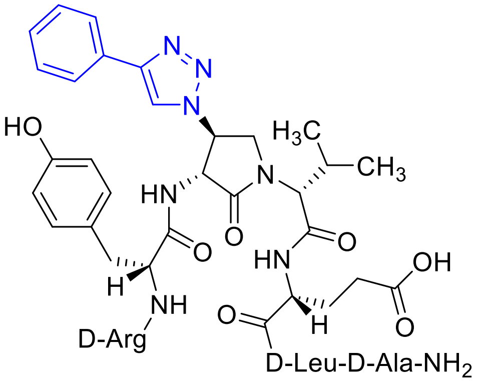

MS Spectrum

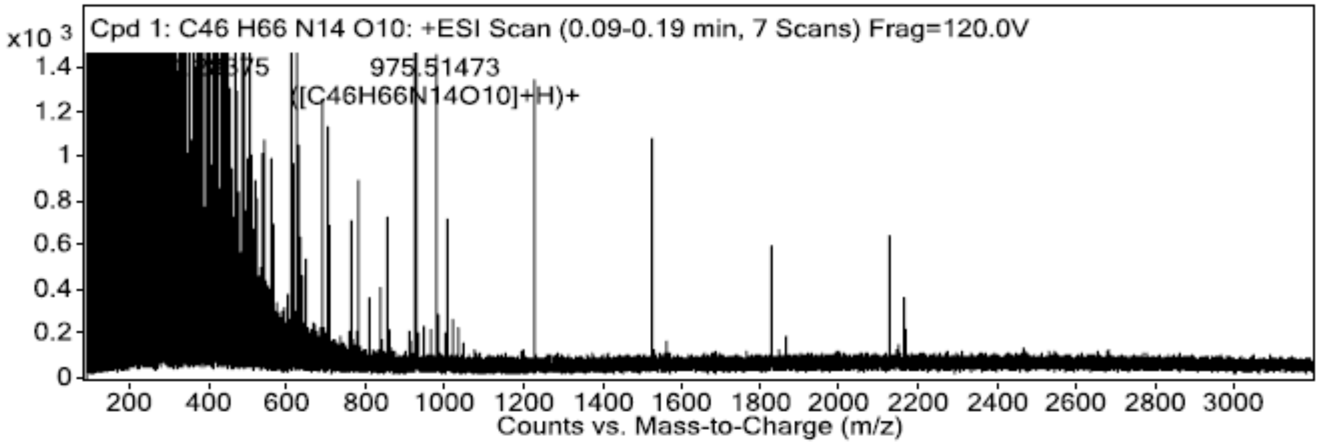

MS Zoomed Spectrum

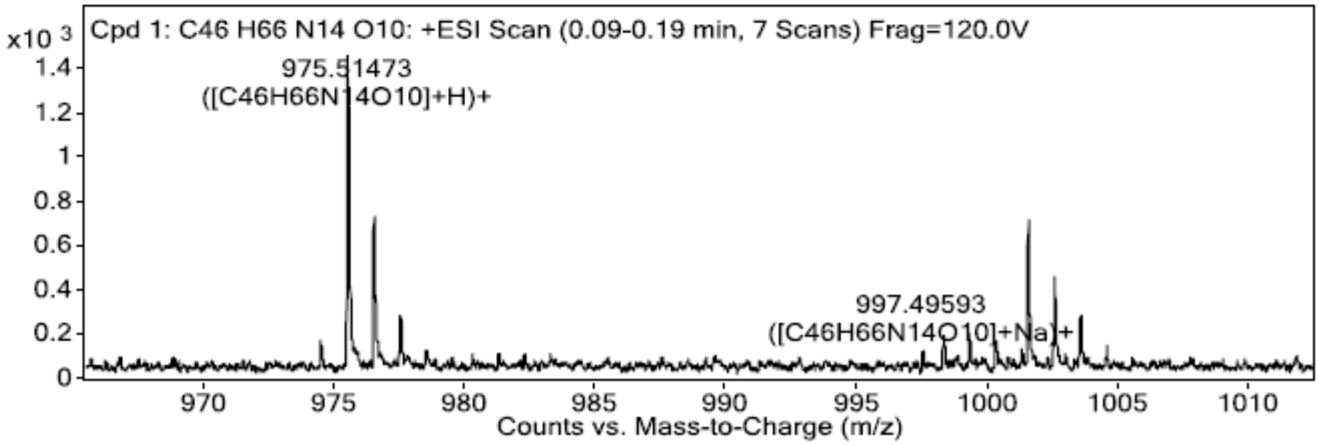

MS Spectrum Peak List

Ion	Formula	Abund	*Expe. m/z*	*Calc. m/z*	Diff(ppm)
(M+H)+	C46H66N14O10	1471.9	975.51473	975.51591	1.21
(M+Na)+	C46H66N14O10	132.72	997.49593	997.49786	1.93

[(3*R*, 4*S*)-β-4-(*p*-MeC$_6$H$_4$)-triazolyl-Agl[3]]-101.10 (2m)

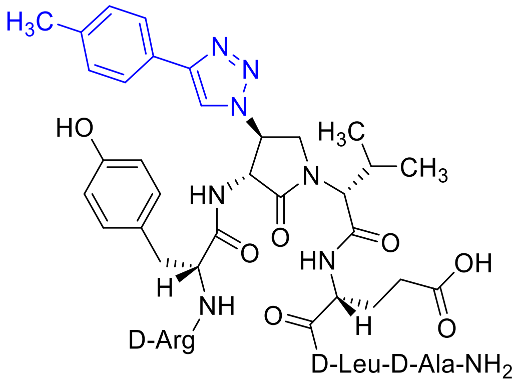

MS Spectrum

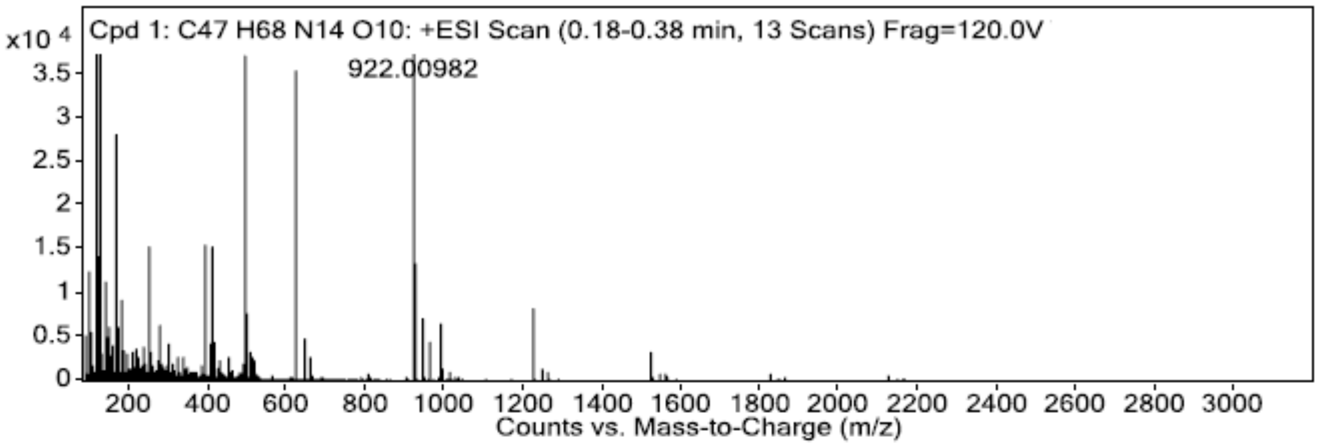

MS Zoomed Spectrum

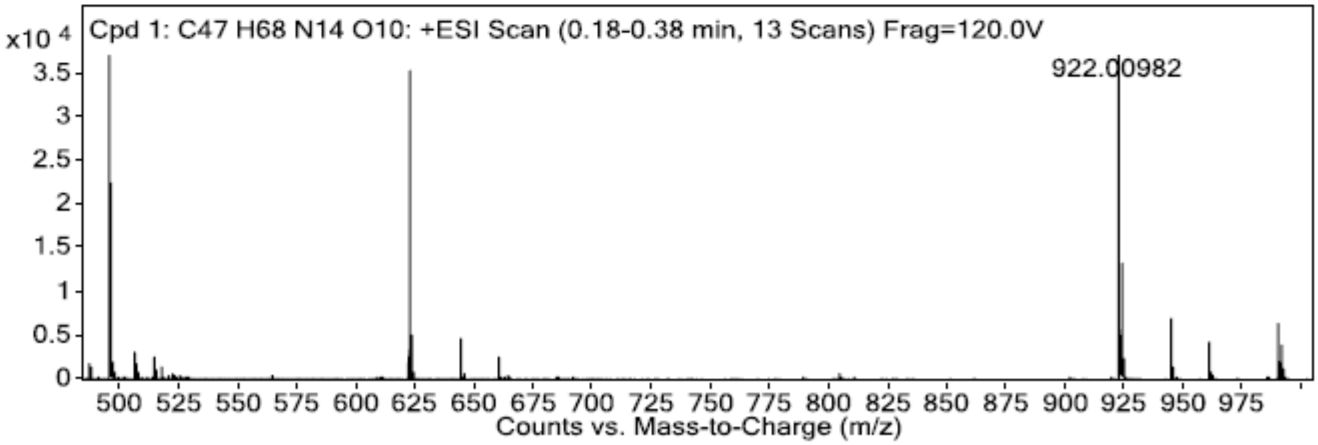

MS Spectrum Peak List

Ion	Formula	Abund	*Expe. m/z*	*Calc. m/z*	Diff(ppm)
(M+2H)+2	C47H68N14O10	37215.08	495.27112	495.26942	-3.43
(M+H)+	C47H68N14O10	6479.84	989.53073	989.53156	0.84

[(3*R*, 4*S*)-β-4-(*m*-$H_2NC_6H_4$)-triazolyl-Agl[3]]-101.10 (2n)

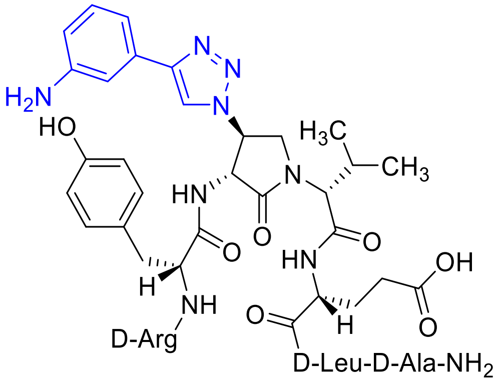

MS Spectrum

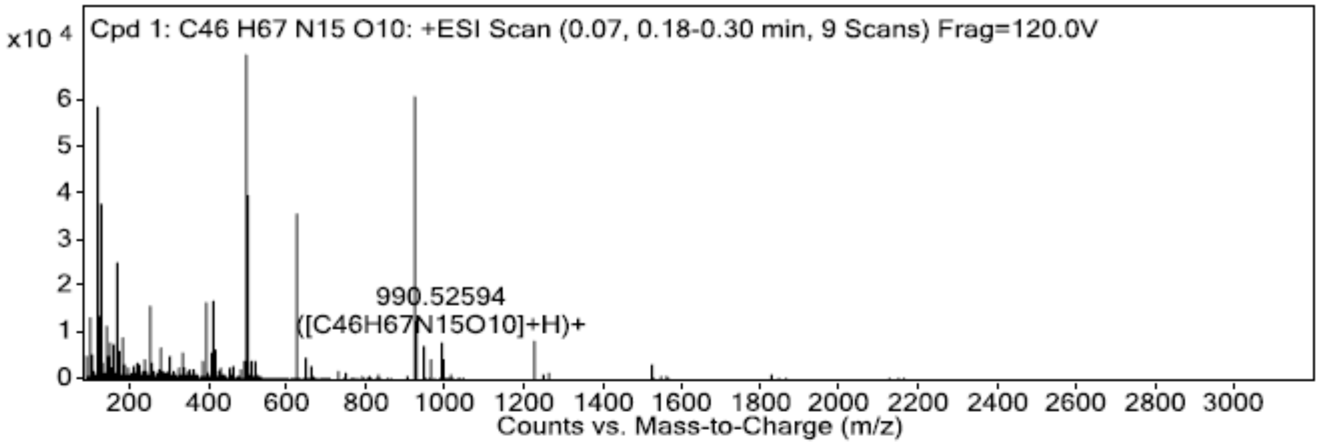

MS Zoomed Spectrum

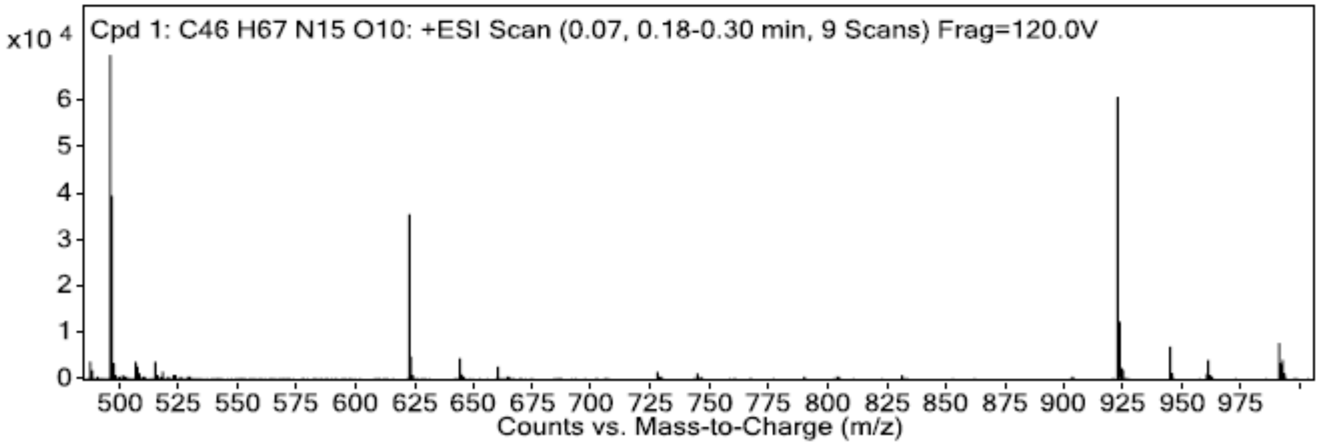

MS Spectrum Peak List

Ion	Formula	Abund	*Expe. m/z*	*Calc. m/z*	Diff(ppm)
(M+2H)+2	C46H67N15O10	70042.22	495.7689	495.76704	-3.74
(M+H)+	C46H67N15O10	7926.13	990.52594	990.52681	0.88

[(3*R*, 4*S*)-β-4-($H_2N(H_3C)_2C$)-triazolyl-Agl[3]]-101.10 (2o)

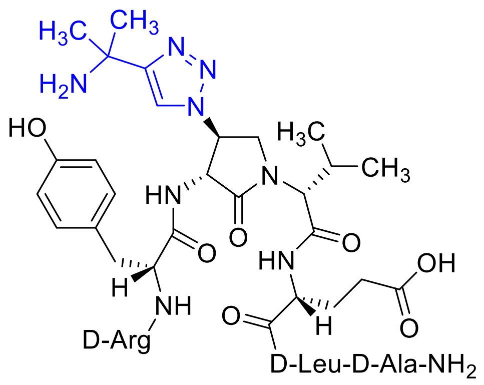

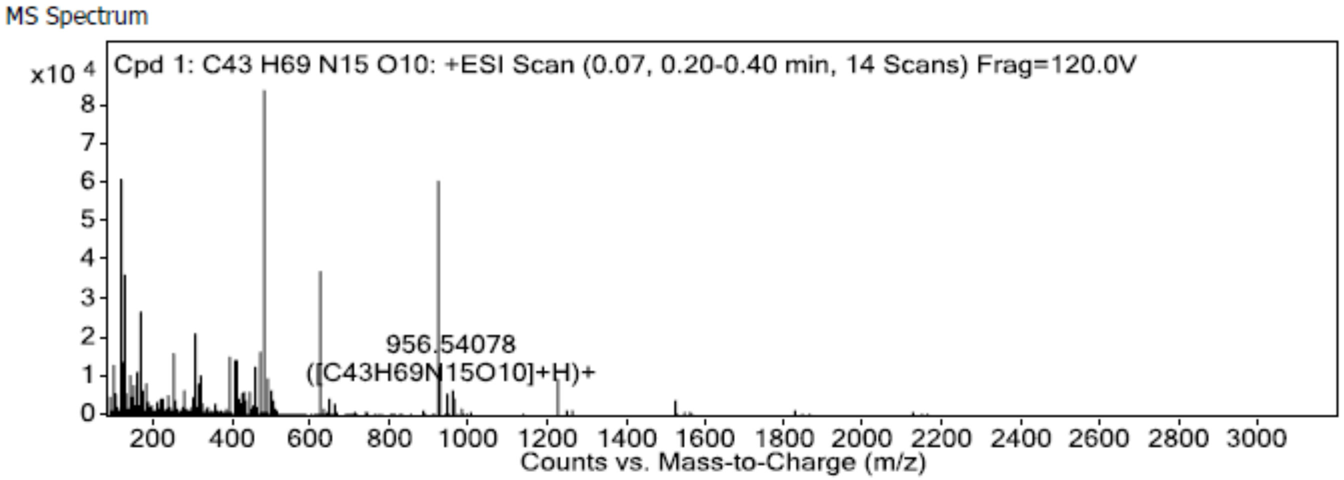

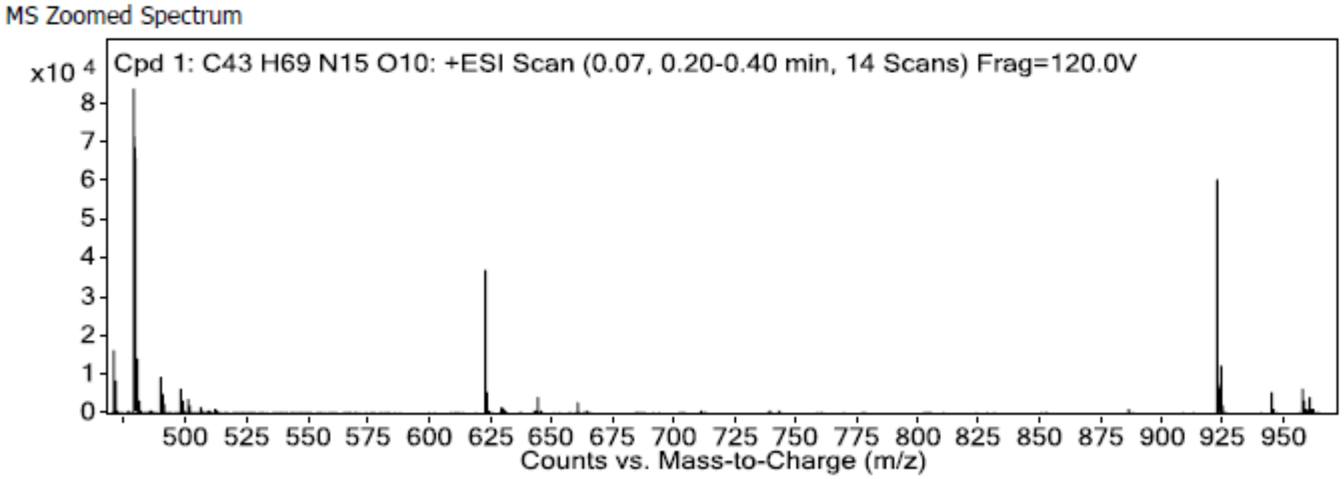

MS Spectrum Peak List

Ion	Formula	Abund	*Expe. m/z*	*Calc. m/z*	Diff(ppm)
(M+2H)+2	C43H69N15O10	84040.01	478.77693	478.77487	-4.3
(M+H)+	C43H69N15O10	6404.33	956.54078	956.54246	1.76

[(3*R*, 4*S*)-β-4-(HOH$_2$C)-triazolyl-Agl[3]]-101.10 (2q)

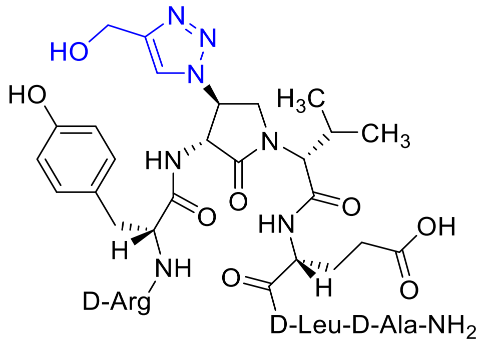

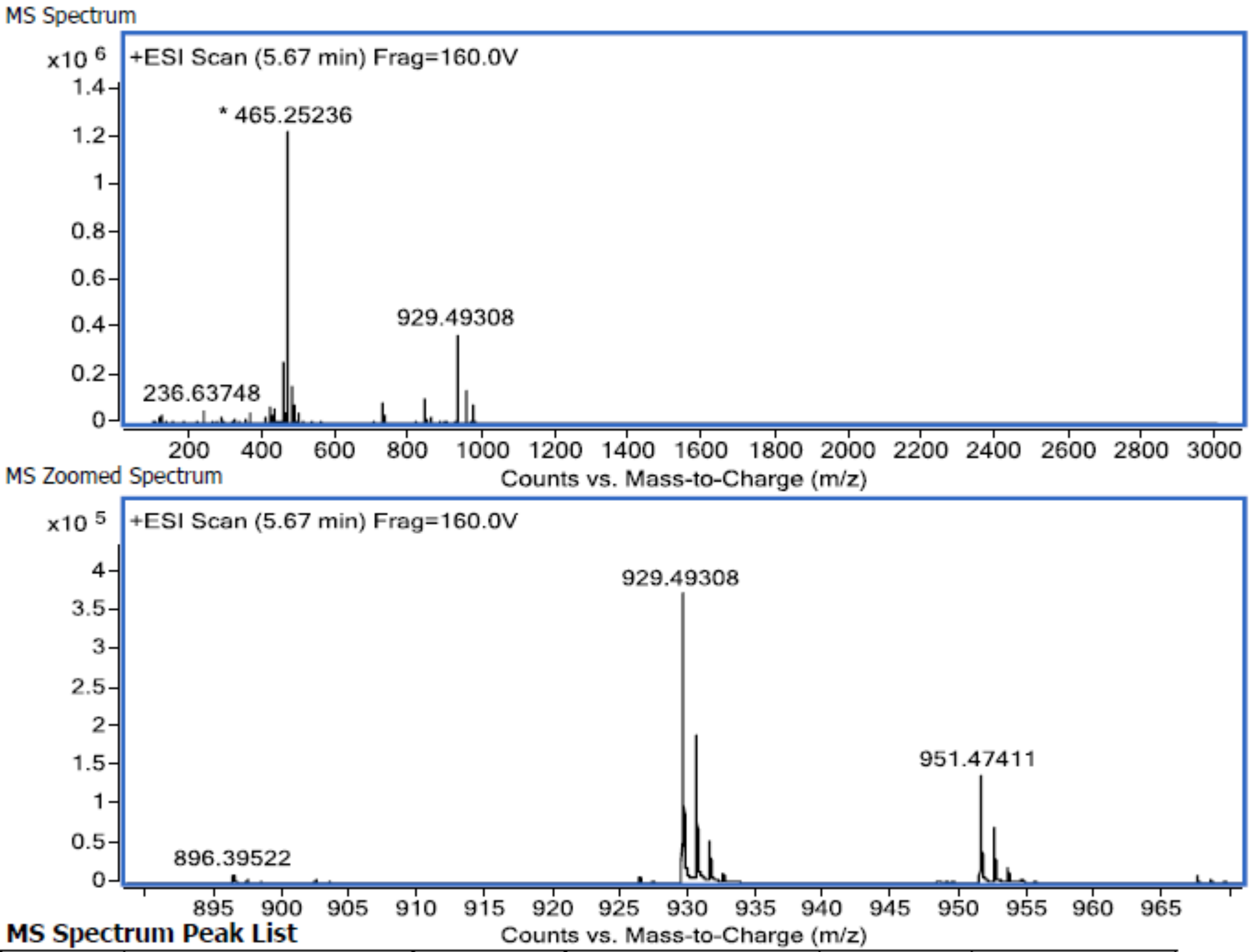

MS Spectrum Peak List

Ion	Formula	Abund	Expe. m/z	Calc m/z	Diff (ppm)
(M+H)+	C41 H64 N14 O11		929.49305	929.49518	2.29

***tert*-Butyl (3*R*, 4*S*, 2'*R*)-2-[3-(Fmoc)amino-4-(1,3-dioxoisoindolin-2-yl)oxy)-2-oxopyrrolidin1-yl]-3-methylbutanoate (5f)**

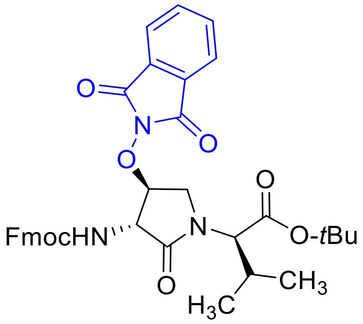

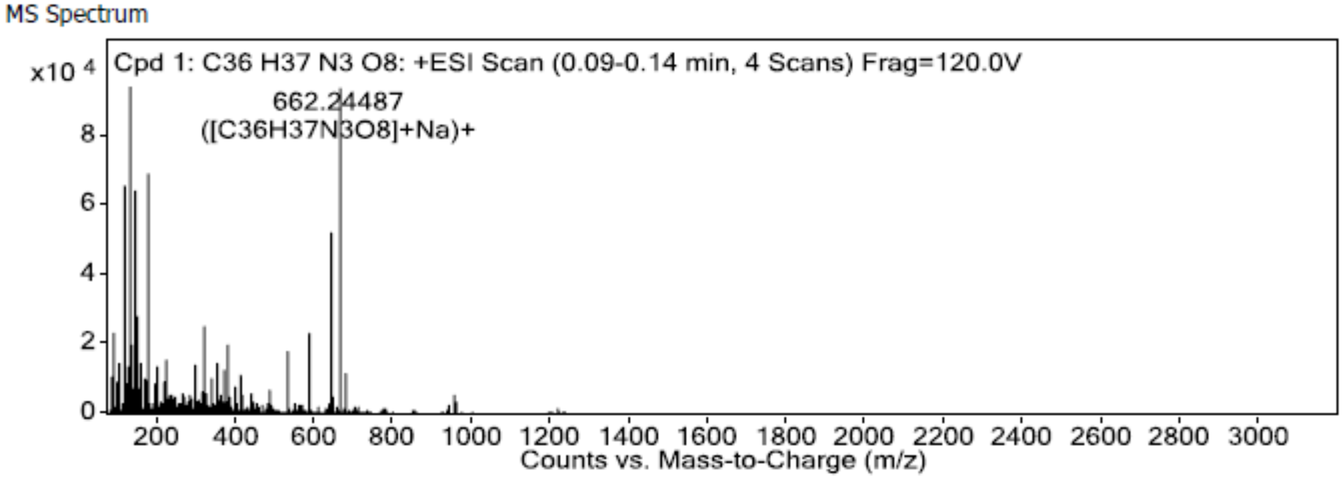

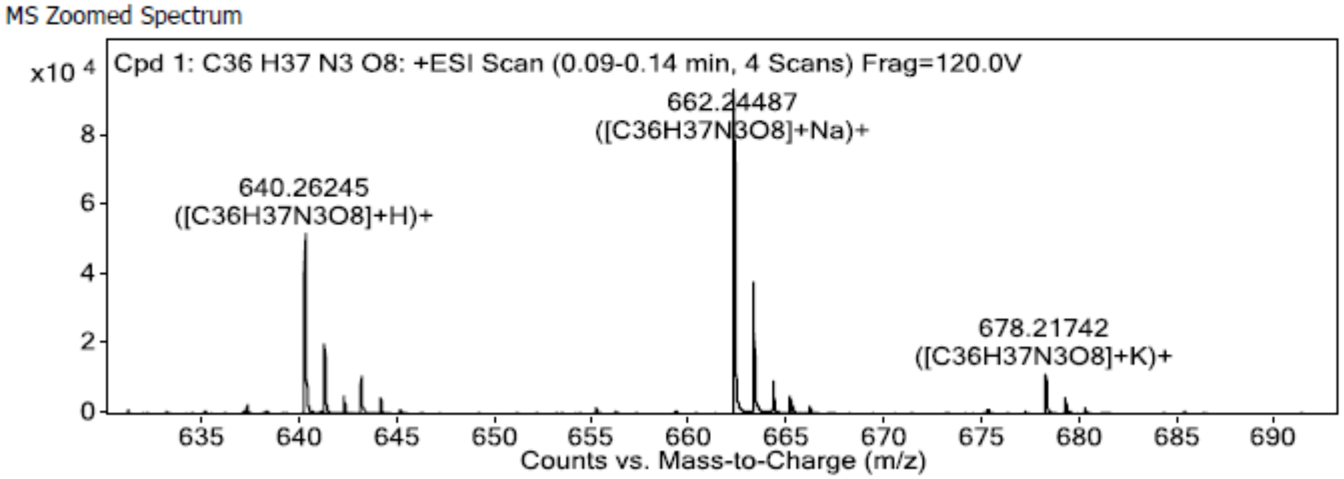

MS Spectrum Peak List

Ion	Formula	Abund	*Expe. m/z*	*Calc. m/z*	Diff(ppm)
(M+H)+	C36H37N3O8	51888.14	640.26245	640.26534	4.51
(M+Na)+	C36H37N3O8	95376.44	662.24487	662.24729	3.65

NMR spectra:

***tert*-Butyl (3*R*, 4*S*, 2'*R*)-2-[3-(Fmoc)amino-4-(1,3-dioxoisoindolin-2-yl)oxy)-2-oxopyrrolidin1-yl]-3-methylbutanoate (5f)**

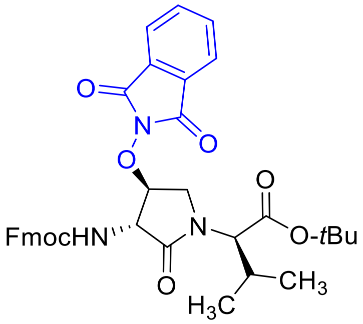

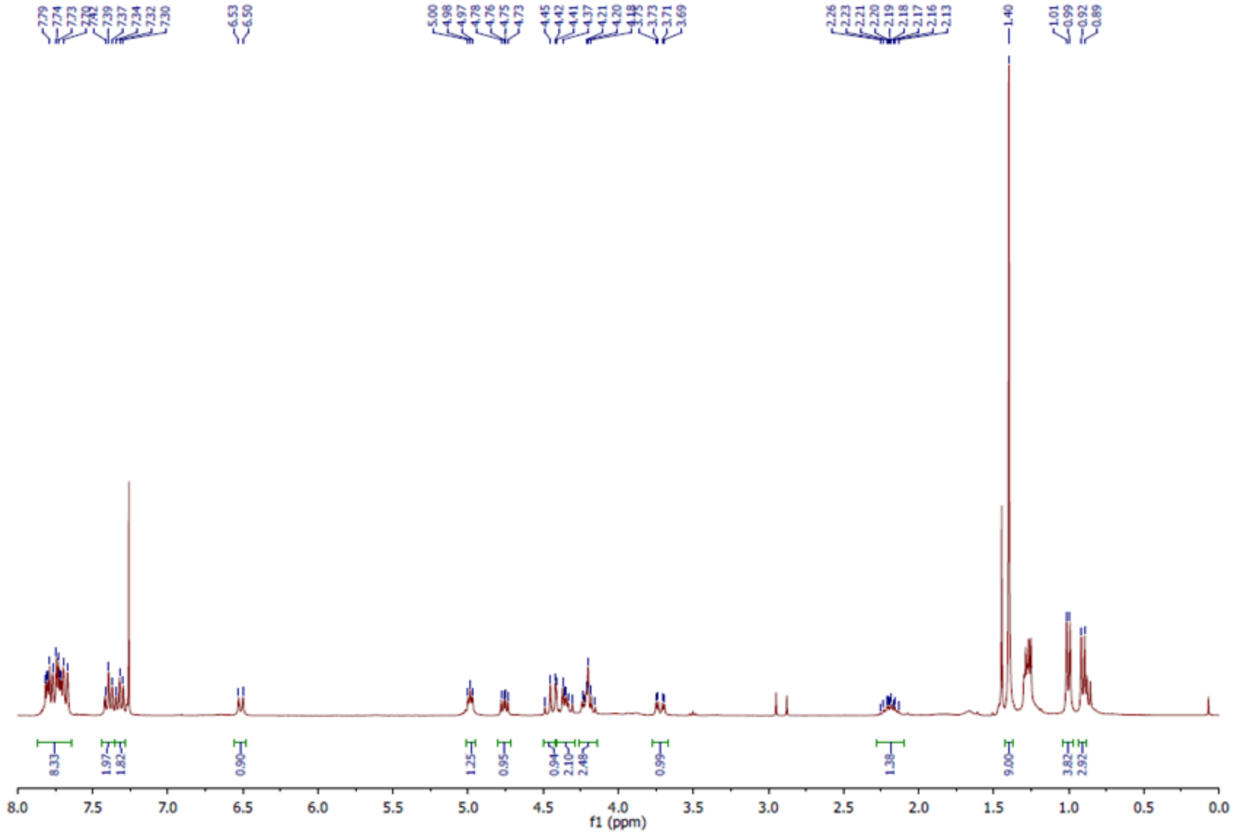

***tert*-Butyl (3*R*, 4*S*, 2’*R*)-2-[3-(Fmoc)amino-4-(1,3-dioxoisoindolin-2-yl)oxy)-2-oxopyrrolidin1-yl]-3-methylbutanoate (5f)**

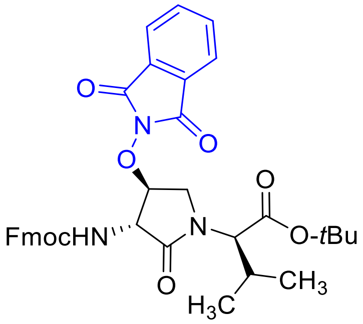

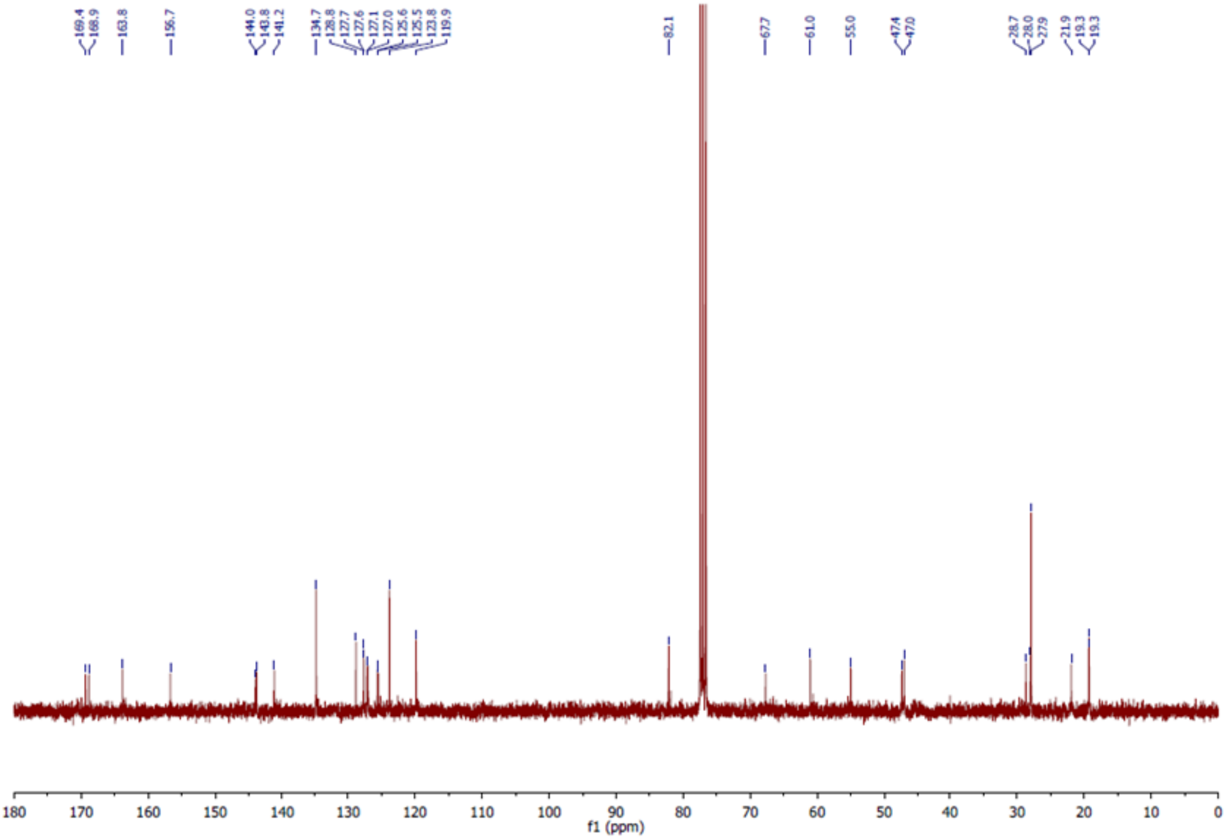

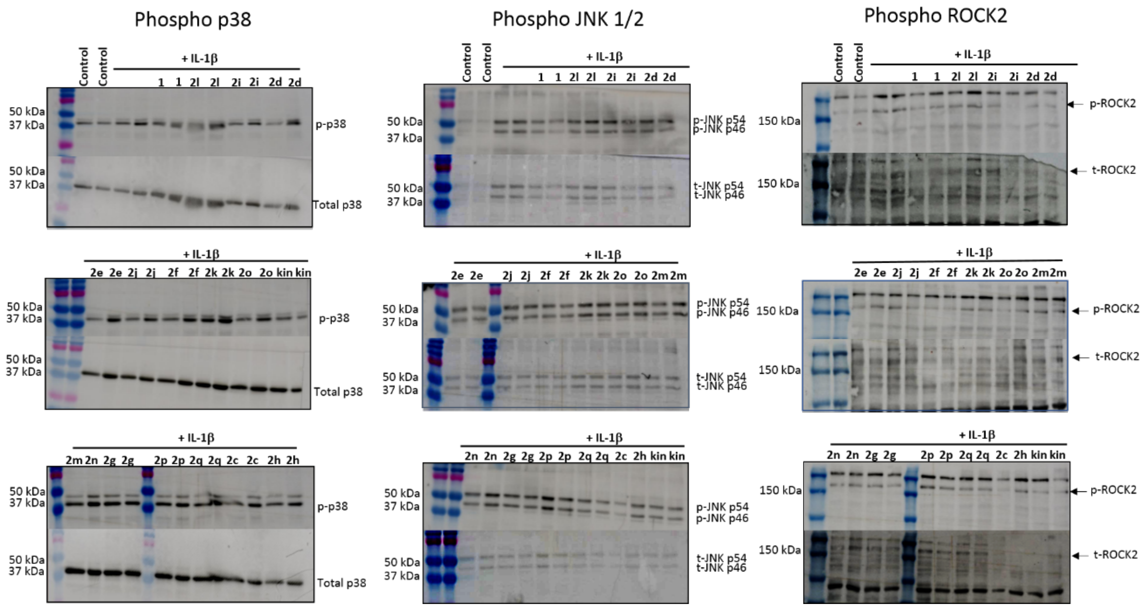

Supplementary Figure 1. Representative Western Blots of the JNK, ROCK2 and p38 proteins. RAW-blue cells were pre-treated with peptides 1 or 2 (1 µM) for 30 min, then stimulated with IL-1β (100 ng/ml) for 15 min. Cell lysates were prepared and lysates (30µg protein per well) were electrophoresed on 10 or 12% resolving gels, transferred to PVDF membranes and incubated with antibodies for these proteins and their phosphorylated forms.
